# New York State Climate Impacts Assessment Chapter 02: New York State's Changing Climate

**DOI:** 10.1111/nyas.15240

**Published:** 2024-12-09

**Authors:** Christopher Lamie, Daniel Bader, Kathryn Graziano, Radley Horton, Kecil John, Natalie O'Hern, Sophia Spungin, Amanda Stevens

**Affiliations:** ^1^ Eastern Research Group, Inc. Concord Massachusetts USA; ^2^ Columbia Climate School Columbia University New York New York USA; ^3^ Eastern Research Group, Inc. New York New York USA; ^4^ Columbia Climate School Columbia University Palisades New York USA; ^5^ Eastern Research Group, Inc. Rochester New York USA; ^6^ New York State Energy Research and Development Authority Albany New York USA

**Keywords:** climate change, future projections, historical observations, precipitation, temperature, New York State, extreme events, sea level rise

## Abstract

Many fundamental aspects of New York State's climate have already begun to change, and the changes are projected to continue—and in some cases, accelerate—throughout the 21st century. This chapter explores observed and projected changes in a variety of physical variables that relate directly to weather and climate, starting with average and extreme air temperature and proceeding to the associated effects on precipitation, extreme events, and core properties of New York's coastal and inland waters. These climate attributes and hazards lead to impacts throughout the eight sectors of this assessment.

## 1 TECHNICAL WORKGROUP KEY FINDINGS

Many fundamental aspects of New York State's climate have already begun to change, and the changes are projected to continue—and in some cases, accelerate—throughout the 21st century. This chapter explores observed and projected changes in a variety of physical variables that relate directly to weather and climate, starting with average and extreme air temperature and proceeding to the associated effects on precipitation, extreme events, and core properties of New York's coastal and inland waters. These climate attributes and hazards lead to impacts throughout the eight sectors of this assessment.

**Key Finding 1: Average and maximum temperatures have increased in New York State since the early 20th century and are projected to continue to rise throughout the 21st century**. The state has warmed more rapidly than the national average, and winter is warming more rapidly than other seasons. Heat waves are expected to occur more often and become more intense, posing greater risks for human health, built infrastructure, ecosystems, and other sectors. New York City is projected to remain the warmest part of the state; northern regions will continue to be relatively cooler while still experiencing large increases in temperature and extreme heat.

**Key Finding 2: New York State has experienced increases in total precipitation and heavy precipitation events, and these trends will continue through the end of this century**. Heavy rainstorms that lead to flooding are projected to become more frequent across the state. Precipitation is expected to increase the most in winter. Lake‐effect snowfall is projected to increase over the next few decades, but as temperatures continue to rise, more winter precipitation near the Great Lakes will fall as rain. Elsewhere in the state, snowfall and snowpack are likely to decrease with warmer winter temperatures.

**Key Finding 3: Climate change is creating conditions that will increase the frequency and severity of many types of extreme events**. Several types of storms are expected to become more intense, with heavier rainfall, stronger winds, and higher storm surge along the coast driven by sea level rise. Short‐term summer droughts could increase due to changing precipitation patterns and increased temperatures. Wildfires are unlikely to become much more common within New York State due to climate change, but air quality impacts from large fires elsewhere in North America could increase in the future.

**Key Finding 4: Sea surface temperature, sea level, and coastal flooding are increasing along New York State's coast**. Sea surface temperatures are rising more rapidly in the state than the global average. Sea level along New York's coastline has risen almost 1 foot in the past century and is projected to increase by another 1–2 feet by mid‐century, making chronic flooding more common in low‐lying coastal neighborhoods. Ocean water is also becoming more acidic as it absorbs excess carbon dioxide from the atmosphere, although stormwater runoff currently has a larger effect on acidity in New York's coastal waters.

**Key Finding 5: New York State's lakes and rivers have experienced increased water temperature, fluctuating water levels, and decreased ice cover, and these changes are expected to intensify in a warmer, wetter future**. Lakes are projected to experience more severe summer heat waves and decreased winter ice cover as temperatures rise in the coming decades. The Great Lakes could experience greater year‐to‐year variability in water levels, driven by periods of drought and extreme precipitation. Flood intensity and damages are expected to increase with extreme rainfall and broader changes in streamflow.

## 2 INTRODUCTION AND BACKGROUND

Authoritative assessments of climate change by the Intergovernmental Panel on Climate Change (IPCC) and the U.S. Global Change Research Program (USGCRP) cite observed evidence of global and national‐scale changes in a wide range of physical variables, including weather and climate (e.g., air temperature and precipitation averages and extremes), conditions in the Earth's oceans (e.g., temperature, sea level), and conditions of the Earth's cryosphere (snow and ice features).^1^, ^2^ Many of the same changes have been observed in New York State, and projections from dozens of climate models agree that these changes will continue through the decades ahead.

This chapter summarizes observed and projected changes in key physical variables within New York State. It is organized as follows:

- This introduction briefly reviews the causes of global climate change; the sources of historical observation data incorporated into this assessment; and the sources, methods, and scenarios that form the basis for the projections of future change that were developed or otherwise selected for this assessment.
- Subsequent sections describe observed and projected changes through figures, tables, and summary text. The sections are organized into major thematic groupings (temperature, precipitation, extreme events, ocean conditions, and lakes and rivers), each containing information about several specific variables. Each variable's discussion presents both observations and projections to the extent they are available, to provide continuity and context.
- A final section discusses compounding events.
- The Traceable Accounts appendix examines each key finding in depth. It provides citations that support each assertion and it presents the authors’ assessment of confidence in each finding.

Readers can find more detailed results in the full data package^3^ and accompanying methodology report^4^ for the future climate projections that were developed for this assessment. These materials are available on the assessment website.

Historical observations and future projections for additional variables appear within this assessment's eight sector‐based technical chapters. The sector chapters examine physical climate or impact variables that are uniquely associated with their specific sectors, whereas “New York State's Changing Climate” presents information on fundamental weather and climate variables and other topics likely to be relevant to multiple sectors. For example, this chapter covers temperature averages and extremes in general, while the agriculture chapter discusses the length of the growing season, winter chill requirements for certain crops, and exceedance of specific temperature thresholds relevant to livestock health.

### 2.1 The causes of global climate change

Climate change is a global challenge driven predominantly by the buildup of heat‐trapping greenhouse gases in the Earth's atmosphere due to human activities. A vast body of scientific work has established the fundamental science of climate change and its causation, as summarized in three decades of comprehensive global and national assessment reports. This statewide assessment, therefore, does not attempt to replicate or reexamine the physical drivers or attribution of climate change, choosing instead to refer to the clear and authoritative work that has already been published by the IPCC and the USGCRP. The most recent assessments published by these bodies include the following conclusions:

- “It is unequivocal that human influence has warmed the atmosphere, ocean, and land. Widespread and rapid changes in the atmosphere, ocean, cryosphere, and biosphere have occurred.”^5^
- Human activities are changing the climate. The evidence for warming across multiple aspects of the Earth system is incontrovertible, and the science is unequivocal that increases in atmospheric greenhouse gases are driving many observed trends and changes. There are more greenhouse gases in the atmosphere primarily because humans have burned and continue to burn fossil fuels for transportation and energy generation. Industrial processes, deforestation, and agricultural practices also increase greenhouse gases in the atmosphere. As a result of increases in the atmospheric concentrations of these heat‐trapping gases, the planet is on average about 2°F (1.1°C) warmer than it was in the late 1800s. No natural processes known to science could have caused this long‐term temperature trend. The only credible explanation for the observed warming is human activities.^6^
- “Observed increases in well‐mixed greenhouse gas concentrations since around 1750 are unequivocally caused by human activities.”^5^
- “Human influence has warmed the climate at a rate that is unprecedented in at least the last 2000 years.”^5^
- “Global surface temperature will continue to increase until at least mid‐century under all emissions scenarios considered. Global warming of 1.5°C and 2°C will be exceeded during the 21st century unless deep reductions in CO_2_ and other greenhouse gas emissions occur in the coming decades.”^5^

The IPCC's Sixth Assessment Report (2021) notes that “Climate change is already affecting every inhabited region across the globe.”^5^ The New York State Climate Impacts Assessment explores the physical, societal, and ecological impacts of the specific changes that have been observed or projected to occur in New York.

### 2.2 Historical observations

Historical observations presented in this chapter come from high‐quality data collection programs, typically operated or coordinated by government agencies such as the National Oceanic and Atmospheric Administration's (NOAA's) National Weather Service. In selecting sources for inclusion, the assessment team has attempted to find the longest available temporal record that also provides strong spatial coverage of New York and an assurance that data have been collected and processed consistently over time and space. Where possible, this chapter uses analyses that have already been vetted against data quality criteria for inclusion in collections such as the USGCRP's indicator platform, NOAA's Climate at a Glance tool, and the U.S. Environmental Protection Agency's (EPA's) climate change indicator suite.

Some climate‐related variables lack reliable long‐term historical records. In such cases, this chapter presents whatever credible information is available but acknowledges its limitations.

This chapter does not claim that all changes observed to date are unequivocally attributed to human‐caused climate change. Nonetheless, other literature has established causal links for many of the observations presented here.

### 2.3 Projections of future change

Many of the projections presented here come from analyses performed specifically for this assessment by a team at Columbia University. The Columbia team developed projections using the most recent suite of global climate models (GCMs) from the Coupled Model Intercomparison Project version 6 (CMIP6), representing the state of the science in climate modeling and consistent with the most recent IPCC assessment (AR6) and the Fifth National Climate Assessment. Columbia statistically downscaled the global model outputs to provide regional resolution within New York by aligning modeled hindcast results with observational data from 27 long‐term weather stations across the state, using a baseline period of 1981–2010 to represent recent conditions. At the time the assessment process began, this baseline was the most recent three‐decade period that had complete observed data as well as hindcast data from all the GCMs that the assessment team used. All 27 stations had sufficient data to project average temperature and precipitation conditions into the future, while 19 of them had sufficient data to project extreme temperature and precipitation events. These data were used to present projections for 12 regions of New York. The modeling team projected future sea level change for three locations that have many decades of historical water level data. Figure 2‐1 shows the assessment regions along with the locations of all stations described above.

**FIGURE 2‐1:**
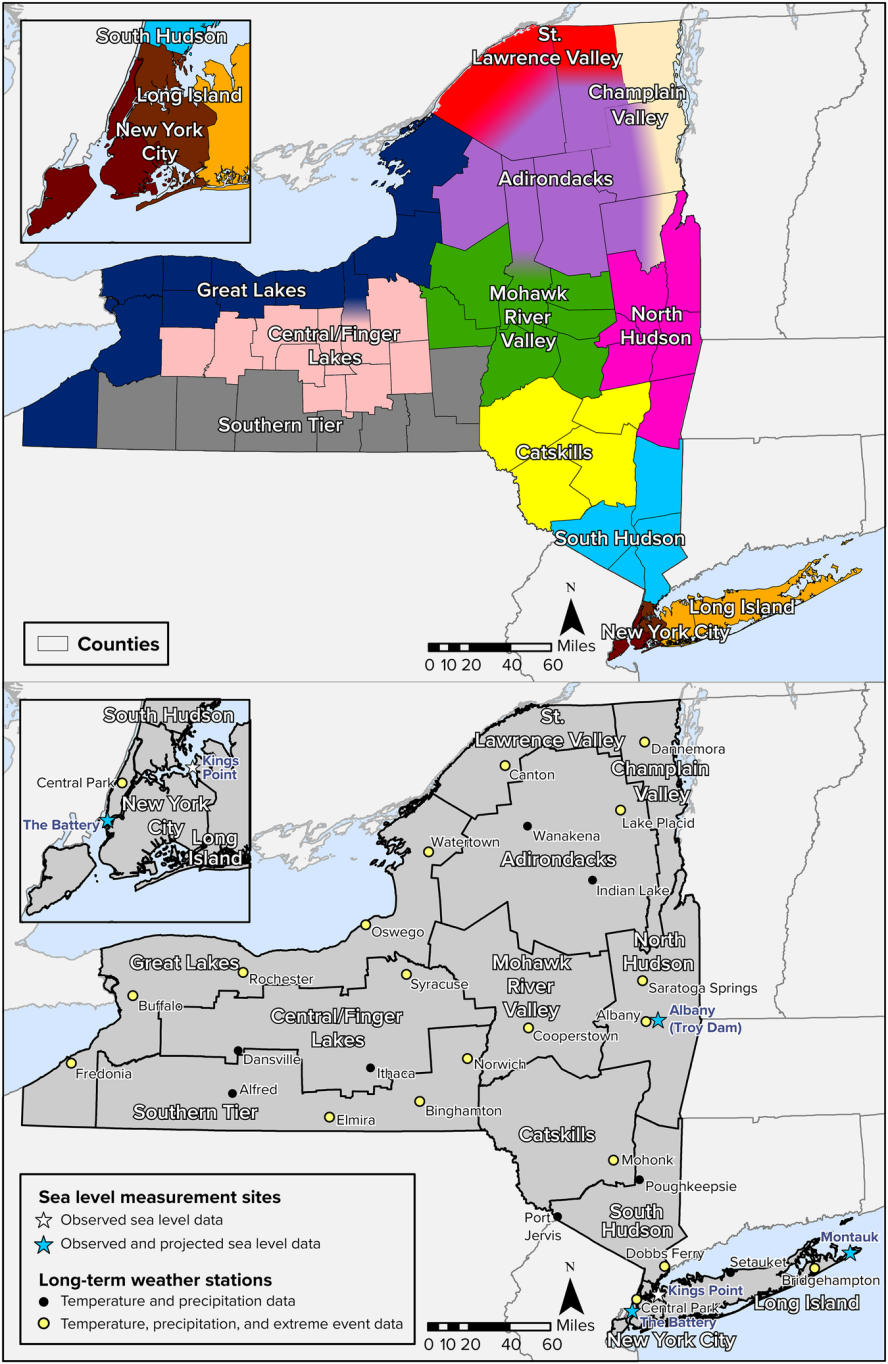
Regions and stations used for this assessment's physical climate observations and projections. Assessment region boundaries were developed for this assessment.^3^ Locations of sea level monitoring stations from NOAA (2023).^7^ Locations of weather stations from NOAA, National Centers for Environmental Information (2012).^8^

The Columbia team's projections include many commonly examined measures of climate change and its direct effects—for example, average and extreme temperature, average/total and extreme precipitation, and sea level change. However, their efforts do not cover every possible variable of interest. This chapter presents projections of selected additional variables based on what the assessment team judged to be the best available published sources. These sources include CMIP6 and CMIP5 results published in other recent climate assessments or scientific literature, as well as a regional model based on CMIP5 GCMs but optimized with additional knowledge of the hydrodynamics and land–water–atmosphere interactions of the Great Lakes and their surrounding region.

BOX 1 **How this chapter presents projections**

Unless noted otherwise, projections presented in this chapter represent an unweighted combination of the results from SSP2‐4.5 and SSP5‐8.5. When a range of two numbers is displayed, it represents the 25th−75th percentiles of the distribution of results from individual climate models, unless noted otherwise. This means 25% of model results were below the given range, 50% were within it, and 25% were above it. Most variables with decades listed actually represent a 30‐year average centered on that decade. For example, “2080s” is the average for 2070–2099. The use of 30‐year averaging is a standard practice for projecting future climate conditions, as such conditions have natural year‐to‐year variability. Sea level projections are based on different methods and use 10‐year averages.

All projections identify a corresponding climate scenario or trajectory. Most, including the Columbia team's projections for this assessment, reference the commonly used Shared Socioeconomic Pathways (SSPs) scenario framework. SSPs are defined by a set of socioeconomic development narratives and delineate future greenhouse gas emissions trajectories.^9^, ^10^ Table 2‐1 provides a reference to five of the most commonly used scenarios, including descriptive terminology that this assessment will use for consistency with other authoritative sources. For context, the table also identifies the projected global average surface temperature change associated with each scenario, according to the IPCC AR6. To provide a single range of possibilities that might be relatively more useful to many of this assessment's users, most of the projections in this chapter present an unweighted combination of the results from two SSPs: middle of the road/intermediate emissions (SSP2‐4.5) and fossil fueled development/very high emissions (SSP5‐8.5).

**TABLE 2‐1:** Commonly used future climate scenarios.

Scenario	Relative level of greenhouse gas emissions	Corresponding SSP narrative	Projected 2021–2040 global avg. temperature change^a^	Projected 2041–2060 global avg. temperature change^a^	Projected 2081–2100 global avg. temperature change^a^
SSP1‐1.9	Very low	Sustainable development: Sustainable development proceeds at a reasonably high pace.	2.7°F (1.5°C) (range: 2.16–3.06°F)	2.88°F (1.6°C) (range: 2.16–3.6°F)	2.52°F (1.4°C) (range: 1.8–3.24°F)
SSP1‐2.6	Low	Sustainable development: Sustainable development proceeds at a reasonably high pace.	2.7°F (1.5°C) (range: 2.16–3.24°F)	3.06°F (1.7°C) (range: 2.34–3.96°F)	3.24°F (1.8°C) (range: 2.34–4.32°F)
SSP2‐4.5	Intermediate	Middle of the road: An intermediate case between SSP1 and SSP3.	2.7°F (1.5°C) (range: 2.16–3.24°F)	3.6°F (2.0°C) (range: 2.88–4.5°F)	4.86°F (2.7°C) (range: 3.78–6.3°F)
SSP3‐7.0	High	Regional rivalry: Unmitigated emissions are high due to moderate economic growth, a rapidly growing population, and slow technological change in the energy sector.	2.7°F (1.5°C) (range: 2.16–3.24°F)	3.78°F (2.1°C) (range: 3.06–4.68°F)	6.48°F (3.6°C) (range: 5.04–8.28°F)
SSP5‐8.5	Very high	Fossil fueled development: Energy demand is high and most of this demand is met with carbon‐based fuels.	2.88°F (1.6°C) (range: 2.34–3.42°F)	4.32°F (2.4°C) (range: 3.42–5.4°F)	7.92°F (4.4°C) (range: 5.94–10.26°F)

*Note*: The two scenarios used most frequently in this assessment are SSP2‐4.5 and SSP5‐8.5, which are highlighted in the table. Corresponding greenhouse gas emissions terminology from IPCC AR6.^5^ SSP narratives adapted from O'Neill et al.^11^ Projected global average surface temperatures from IPCC AR6 Table SPM.1, converted from Celsius in the original source.^5^

^a^ Global average surface temperature change relative to 1850–1900; best estimate and “very likely” range are listed.

The IPCC has not assigned likelihoods to the scenarios in Table 2‐1, and scientists and policymakers continue to debate which of these trajectories seems most plausible. The projections methodology report^4^ provides a detailed rationale for focusing on SSP2‐4.5 and SSP5‐8.5 in the projections developed for this assessment. Among other factors, the methodology report notes that even if greenhouse gas emissions are likely to be lower than SSP5‐8.5's trajectory, retaining SSP5‐8.5 can help to capture the broad range of plausible climate impacts, including high‐consequence impacts that are critical for risk management. The full data set allows users to explore scenario‐specific results and differences between scenarios for average temperature and precipitation.

BOX 2 **Comparing scenarios**

The modeled results from two scenarios have been combined in many places to provide a single range of potential outcomes, which some users may find more useful than multiple sets of numbers. However, it is still important to recognize that no one emissions scenario has been “locked in.” The very reason for modeling multiple scenarios is that there are several paths the world could take. If the world takes serious action to reduce greenhouse gas emissions and control future warming, the resulting impacts could be closer to the low end of the projected range, or perhaps even lower.

Figure 2‐2 provides an example of how the two main scenarios analyzed for this assessment diverge over time. This example shows ensemble averages (i.e., averages of all climate models) for the intermediate and very high emissions scenarios for one assessment region—North Hudson—and one variable, annual average temperature. Other variables show a similar divergence, as most are fundamentally based on temperature.

**FIGURE 2‐2:**
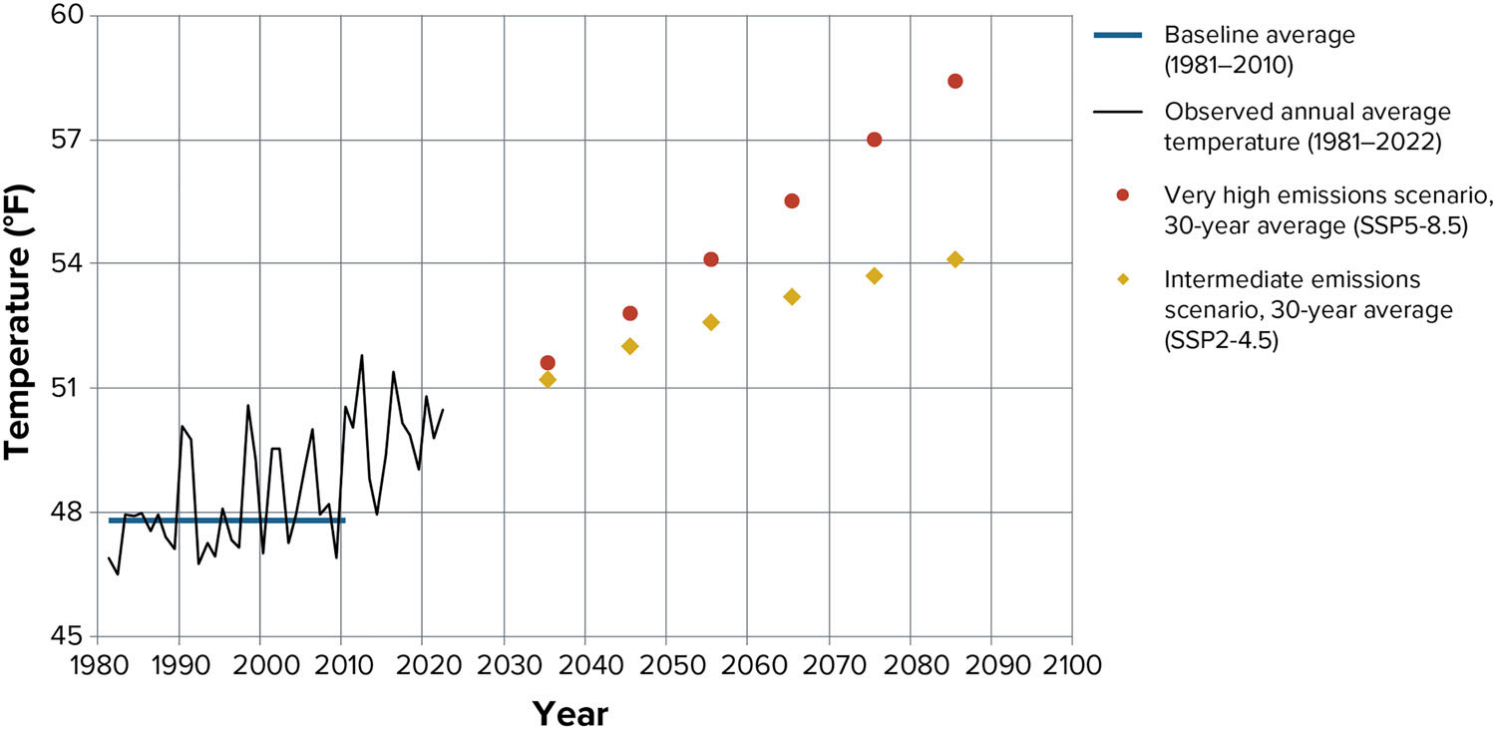
Observed annual average temperature, 1981–2022, and projected annual average temperature during the 21st century in the North Hudson assessment region. The graph shows all‐model averages for the SSP2‐4.5 and SSP5‐8.5 greenhouse gas emissions scenarios. Historical data from NOAA, NRCC (2023).^12^ Future data from projections developed for this assessment.^3^

More information about the SSPs from a New York State perspective will be found in “Population Projections for New York State,” an analysis that will be published at a later date. It presents detailed projections of the future population in New York, derived from the SSPs and the plausible future changes in population and development that they represent. This exploration of the state's future demographics and settlement patterns (e.g., population growth near the coast) offers context for assessing the impacts of concurrent changes in climate hazards.

Additional information about the climate projections developed for this assessment, including sources of baseline data and a comparison with projections used in New York's 2011 and 2014 ClimAID assessments, can be found in the projections methodology report.^4^

Some weather and climate phenomena and resulting impacts are too complex or too dependent on other influencing factors to model reliably with current technologies. In such cases, the assessment presents the best available qualitative information and acknowledges the lack of robust modeling.

## 3 TEMPERATURE

### 3.1 Average temperature

Average temperature is a fundamental attribute of climatology. Here, the concept refers to daily average air temperatures near ground level, averaged over an entire year. This measure naturally obscures day‐to‐day and seasonal variations, and it does not characterize the extreme ends of the temperature distribution, which are responsible for many of the most severe impacts discussed throughout this assessment's sector chapters. Nonetheless, changes in average temperature provide a simple indication of how the temperature distribution is shifting over time, as represented by the center of the distribution.

#### 3.1.1 Historical observations

Based on data from 1981 to 2010, annual average temperatures range from 40.7°F in the Adirondacks to 55.2°F in New York City.^3^ Although the southern part of the state is the warmest on average, summer temperatures along the immediate shoreline are moderated by sea breezes.^4^

From 1901 to 2022, average temperatures in New York State increased by almost 2.6°F, and the warmest 10‐year periods in recorded history have occurred since 2000 (Figure 2‐3).^13^ Among the 27 long‐term weather stations that this assessment used for downscaling projections, all experienced warming, and 24 had warming trends that were statistically significant to a 99% confidence level over the 1901–2020 time period.^4^ As a whole, New York State warmed at an average rate of approximately 0.21°F per decade from 1901 to 2022, significant at the 99% level.^14^ This rate of warming is higher than the contiguous 48 states’ average rate of 0.17°F per decade over the same time frame.^14^ These long‐term average rates of change are based on ordinary least‐squares linear regression from 1901 to 2022. Additional statistical testing of the data in Figure 2‐3 shows that the warming in New York State has occurred at a faster rate over the last 40 years (1983–2022) than over the full time series (1901–2022).^4^

**FIGURE 2‐3:**
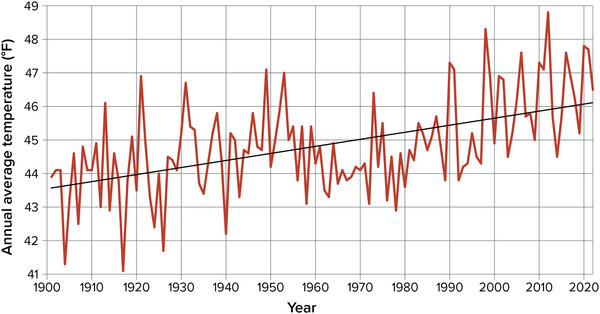
Annual average temperature in New York State, 1901–2022. Daily mean air temperatures are averaged over the entire year, calculated for each NOAA climate division as part of NOAA's nClimDiv data set, and then averaged across all of New York's climate divisions, weighted by each division's area. The black line shows the ordinary least‐squares linear trend (+0.21°F per decade), which is significant to a 99% confidence level (*p* < 0.001). Data from NOAA (2023).^14^

#### 3.1.2 Projections of future change

Annual average temperatures are projected to increase in all regions of New York progressively throughout the 21st century (Figure 2‐4). Across the state, annual average temperatures are projected to increase by 2.5–4.4°F by the 2030s, 3.8–6.7°F by the 2050s, and 5.1–10.9°F by the 2080s, depending on global greenhouse gas emission rates.^3^

**FIGURE 2‐4:**
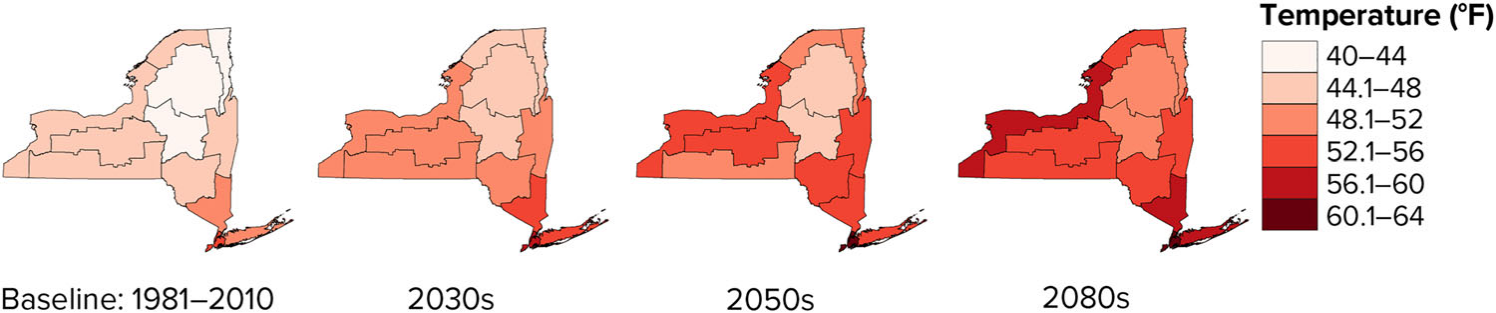
Projected annual average temperature in New York State during the 21st century. The maps show median (50th percentile) modeled results from a blend of the SSP2‐4.5 and SSP5‐8.5 greenhouse gas emissions scenarios. Data from projections developed for this assessment.^3^

Regionally, the largest changes are projected for the St. Lawrence Valley, Champlain Valley, Adirondacks, and Great Lakes assessment regions, with annual average temperatures projected to increase by 4.6–6.7°F by the 2050s and 6.1–10.9°F by the 2080s, compared with the 1981–2010 baselines. New York City is projected to remain the warmest part of the state, reaching an annual average temperature of 59.2–61.2°F by the 2050s and 60.8–65.0°F by the 2080s—about 6–10 degrees warmer than it was from 1981 to 2010.^3^ This means New York City's average temperature by the 2080s is projected to be on par with the 20th‐century average for Birmingham, Alabama (about 63°F).^14^ (This comparison applies to average annual temperature only, and does not imply that other climate metrics, such as seasonal average temperature, daily temperature extremes, or precipitation, would change to the same degree.) The Adirondacks are projected to remain New York's coldest region, although the annual average temperature is projected to increase from 40.7°F during the 1981–2010 baseline period to 45.3–47.3°F by the 2050s and 46.8–51.3°F by the 2080s.^3^

### 3.2 Seasonal temperature

New York experiences drastic seasonal changes in temperature. This seasonality is central to many of the rhythms of life across the state, from agricultural production to energy use to traditional seasonal recreational activities and the local economies that depend on them. It also creates the conditions in which New York's many varied ecosystems thrive. Thus, seasonal average temperature—daily mean temperatures averaged over an entire season—can provide a useful indication of how climate change might affect processes that depend on the temperature at a particular time of year, such as frozen conditions in winter. Analyzing seasonal average temperatures can also reveal whether certain seasons are changing more rapidly than others.

#### 3.2.1 Historical observations

Winter is New York's coldest season, with an average daily temperature from 1901 to 2022 of 22.2°F (Figure 2‐5). Summer (June–August) is the warmest season, with an average temperature of 66.1°F over the same period. Fall (September–November) and spring (March–May) lie between these extremes, with average temperatures of 48.1°F and 42.9°F, respectively, over the period 1901–2022.

**FIGURE 2‐5:**
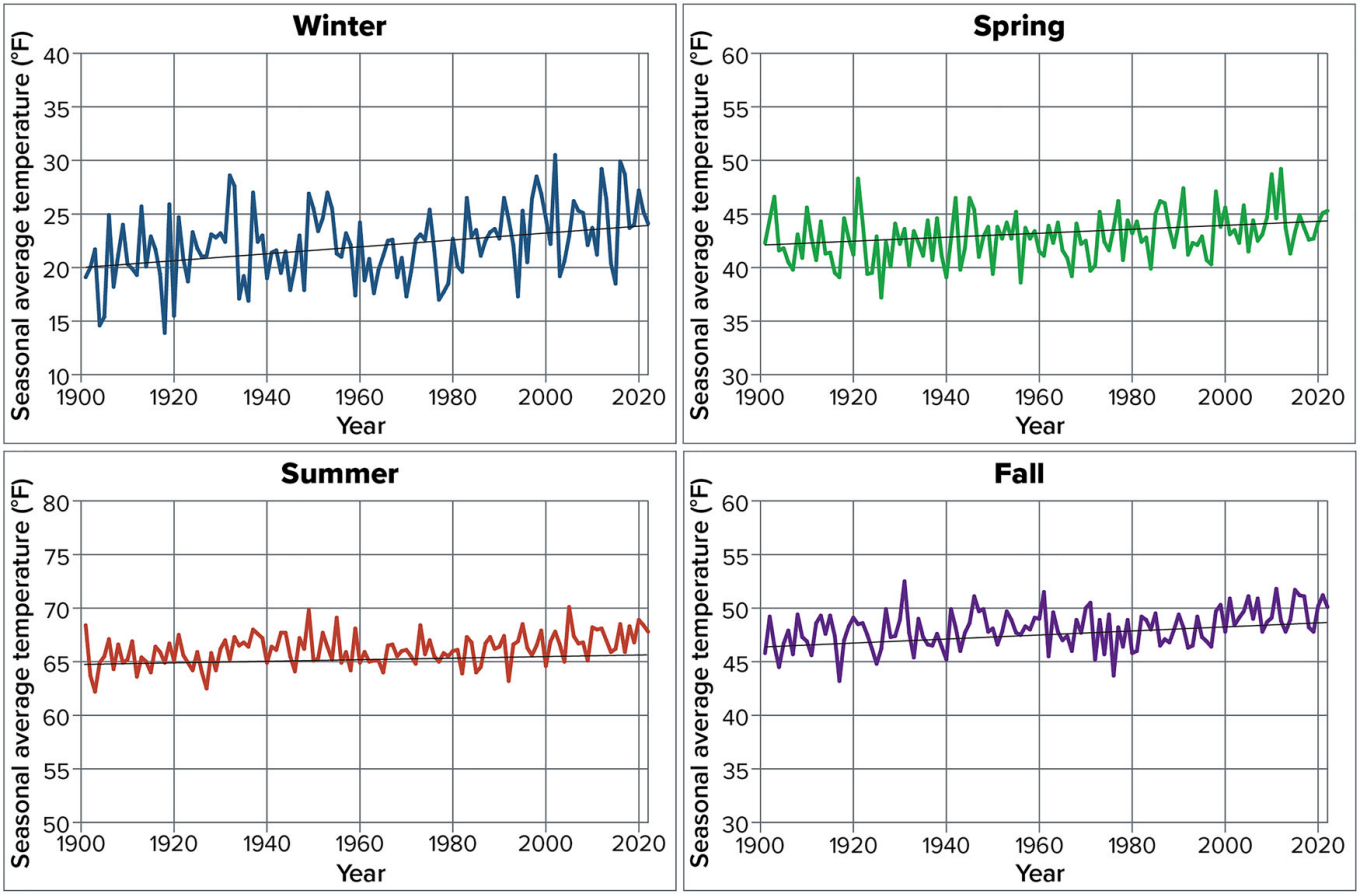
Seasonal average temperatures in New York State, 1901–2022. Daily mean air temperatures are averaged over each season, calculated for each NOAA climate division as part of NOAA's nClimDiv data set, and then averaged across all of New York's climate divisions, weighted by each division's area. Seasons are defined meteorologically: spring is March–May, summer is June–August, fall is September–November, and winter is December–February (plotted here at the year when the winter season ended; e.g., December 2021–February 2022 is plotted as 2022). Ordinary least‐squares linear trends are shown as black lines for reference; winter (+0.34°F per decade, *p* < 0.001), spring (+0.28°F per decade, *p* = 0.001), summer (+0.15°F per decade, *p* < 0.001), and fall (+0.18°F per decade, *p* < 0.001) changes are statistically significant to a 99% confidence level. Data from NOAA (2023).^14^

National and regional analyses consistently show that winter is warming more rapidly than any other season across the contiguous 48 states,^15^ particularly in the Northeast,^16^ and New York follows suit. The state's winters have warmed at a rate almost double that of spring and fall, and more than double that of summer (Figure 2‐5). Over the period 1901–2022, winter average temperatures increased at an average rate of 0.34°F per decade, compared with 0.18°F in spring, 0.15°F in summer, and 0.18°F in fall (Figure 2‐5). As with annual average temperatures in Section 3.1.1, the seasonal trends reported here are based on linear regression slopes over the full period of record.

#### 3.2.2 Projections of future change

Average seasonal temperatures are projected to increase across all regions of New York, compared with the 1981–2010 baseline (Table 2‐2). Winter temperatures are projected to increase the most.

**TABLE 2‐2:** Projected seasonal temperatures in New York State during the 21st century.

Season	2050s change compared with baseline	2080s change compared with baseline
Winter	3.9–8.2°F	5.5–12.5°F
Spring	3.2–6.2°F	4.7–9.7°F
Summer	3.8–6.5°F	5.3–11.1°F
Fall	3.6–6.5°F	5.3–10.7°F

*Note*: Modeled change in degrees relative to 1981–2010 baseline, based on a blend of the SSP2‐4.5 and SSP5‐8.5 greenhouse gas emissions scenarios. The low end of each range represents the 25th percentile of the assessment region with the smallest change. The high end is the 75th percentile of the region with the largest change. Data from projections developed for this assessment.^3^

The more northerly parts of the state, such as the Champlain Valley, North Hudson, St. Lawrence Valley, Adirondacks, and Great Lakes assessment regions, are projected to experience the largest seasonal changes, particularly in winter. Climate modeling suggests that Long Island will have the least change in all seasons. Figure 2‐6 shows detailed projections for three assessment regions selected to provide geographic and climatological diversity.

**FIGURE 2‐6:**
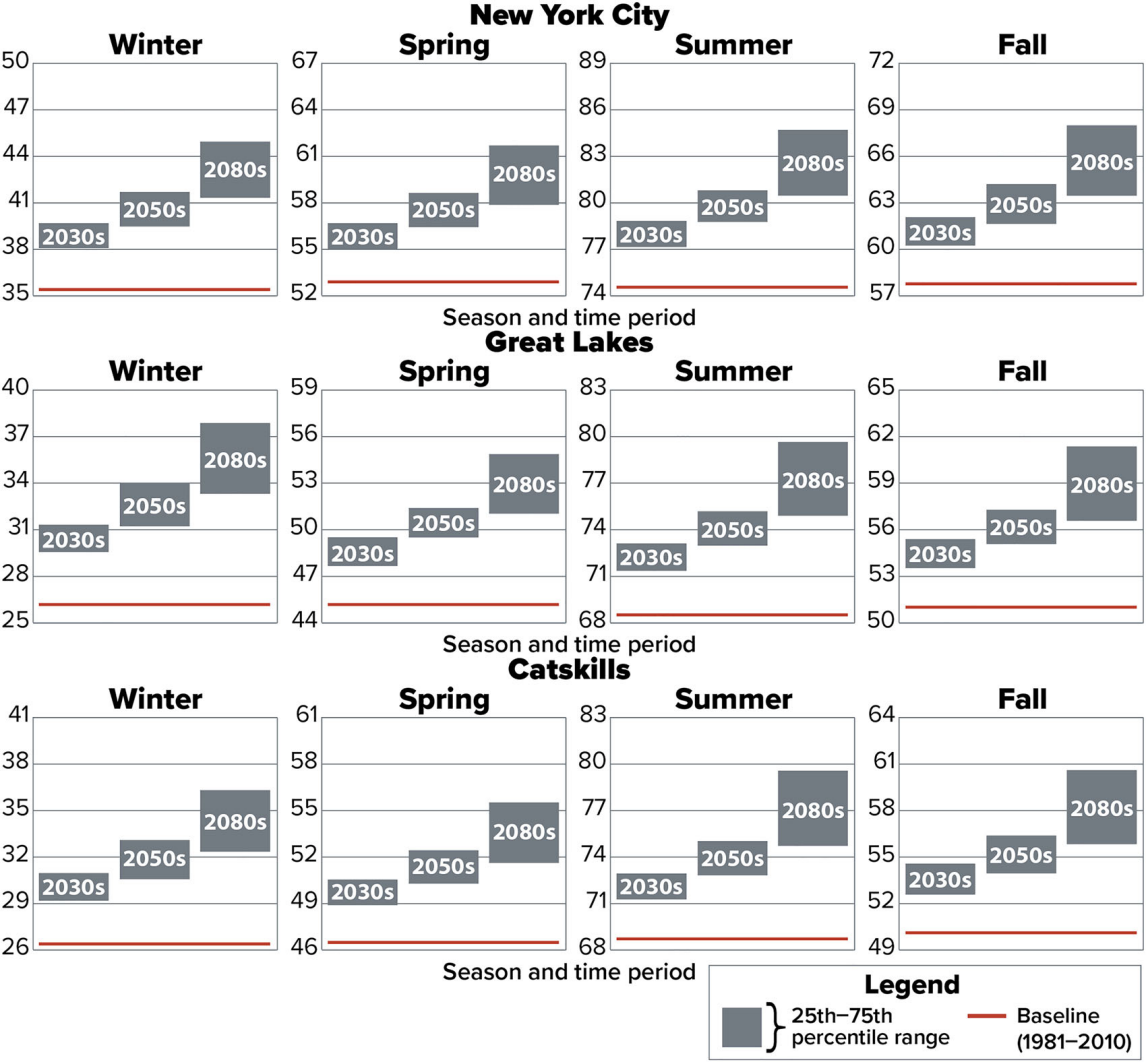
Projected seasonal average temperatures in the New York City, Great Lakes, and Catskills assessment regions during the 21st century. The gray bars represent the 25th−75th percentile range of projected seasonal temperatures from a blend of the SSP2‐4.5 and SSP5‐8.5 greenhouse gas emissions scenarios, while the red line represents the 1981–2010 baseline for each season. When making visual comparisons across rows or down columns, readers should note that while every panel shows a 14‐degree range marked at 2‐degree intervals, each panel does not show the exact same 14‐degree range, as ranges were selected to encompass the baseline and projections for each specific region and season. Data from projections developed for this assessment.^3^

### 3.3 Extreme heat and heat waves

Very hot days occur as a natural part of day‐to‐day variation in weather. However, evidence worldwide shows that temperatures are more frequently exceeding thresholds historically considered to be “unusual” or “extreme.”^1^ This phenomenon occurs as warming shifts the entire distribution of temperatures, pushing more daily temperatures beyond thresholds once considered rare. Extreme heat can pose risks for human health, built infrastructure, agriculture, ecosystems, and more, as this assessment's sector chapters explore in detail. Extremes can pose particular danger when they persist for several days—a phenomenon known as a heat wave or extreme heat event.

Meteorologists and climate scientists use a variety of definitions and thresholds to identify and characterize unusually hot days and heat waves. These definitions often refer to the historical occurrence of such events at a particular location as a baseline, which means that the temperature threshold for “extreme” can vary from one location to another, even within the same state. For this assessment, unless noted otherwise, a “heat wave” is defined as 3 or more consecutive days with maximum temperatures at or above 90°F.

#### 3.3.1 Historical observations

Urban heat islands are urbanized areas that experience higher temperatures than outlying areas because the built environment (buildings, roads, and other infrastructure) absorbs and re‐emits the sun's heat more than natural landscapes such as forests and water bodies.^17^

Days over 90°F are not uncommon in New York City, which is located in the southern part of the state and where the urban heat island effect (defined in the box above) exacerbates high temperatures. From 1981 to 2010, the weather station at Central Park recorded an average of 17 days per year over 90°F, an average of 4 days per year over 95°F, and an average of two heat waves per year. In contrast, days hotter than 90°F are fairly uncommon in the northern part of the state. For example, from 1981 to 2010, Lake Placid experienced an average of only 1 day a year above 90°F, no days above 95°F, and an average of one heat wave every 10 years.^3^

In some parts of New York State, temperatures occasionally exceed 100°F. In Albany, the highest daily temperature ever recorded was 104°F in 1911.^18^ The hottest daily temperatures recorded in Central Park and in Rochester occurred on the same day in 1936, when temperatures reached 106°F and 102°F, respectively.^19^, ^20^ Other cities around the state have yet to exceed 100 degrees: The hottest temperature ever recorded in Buffalo was 99°F in 1948.^21^

Most of the 19 weather stations that were the focus of this assessment have records dating back to at least the first half of the 20th century. Considering the full period of record available for each station and using the definition of a heat wave as 3 or more consecutive days with maximum temperatures at or above 90°F, most of the stations have not yet experienced a statistically significant change in the number of heat waves per year (based on first‐order screening via ordinary least‐squares linear regression and looking for significance to a 95% confidence level). However, Central Park—the station with the longest record, dating back to 1869—has experienced a significant increase. There, the number of heat waves per year averaged 1.3 from 1869 to 1900, 1.8 from 1901 to 1950, 2.3 from 1951 to 2000, and 2.4 from 2001 to 2022.^22^

An EPA analysis of heat waves in 50 large metropolitan areas nationwide over the 1961–2021 period using a different definition of “heat wave” found that heat wave frequency increased significantly (to at least a 95% level) in both Albany and Buffalo.^23^ Heat wave duration and the length of heat wave season also increased significantly in Albany over the same period.^23^ This analysis found no significant change in heat wave characteristics in Rochester over the 1961–2021 period. It excluded New York City because of inconsistency in available data. This particular analysis defined a heat wave as 2 or more consecutive days when the daily minimum apparent temperature (the actual temperature adjusted for humidity) in a particular city exceeded the 85th percentile of historical July and August temperatures. The emphasis on daily minimum temperature reflects the importance of nighttime warming, which poses a particular danger to people without access to cooling, as hot nights prevent the body from recovering from hot daytime conditions.^13^, ^24^ Additional analysis of data across New York State indicates that summer nights are becoming warmer, with more very warm nights (those with a minimum temperature of 70°F or higher) in 2010–2014 than in any prior 5‐year period dating back to 1900.^13^ In Central Park, five of the hottest 10 minimum temperatures ever recorded have occurred during the 21st century.^25^ These findings are consistent with analyses of single‐day minimum and maximum temperatures over the entire contiguous United States, which have shown that lows (typically nighttime temperatures) have warmed much more rapidly than daytime highs, especially since around 2000.^26^

#### 3.3.2 Projections of future change

The number of days above 90°F is projected to increase across New York State during the 21st century (Figure 2‐7). At the 19 weather stations with enough data to calculate changes in extreme events for this assessment, the average number of days per year over 90°F ranged from 1 (Lake Placid) to 18 (Dobbs Ferry) during the 1981–2010 baseline period. These two stations are projected to continue to have the least and the most days above 90°F, respectively, in the future. Lake Placid is projected to experience 6–16 days per year above 90°F by the 2050s and 12–45 days by the 2080s. Dobbs Ferry is projected to have 41–64 days per year above 90°F by the 2050s and 48–87 days by the 2080s. Dobbs Ferry and Saratoga Springs are expected to experience the largest increases.^3^

**FIGURE 2‐7:**
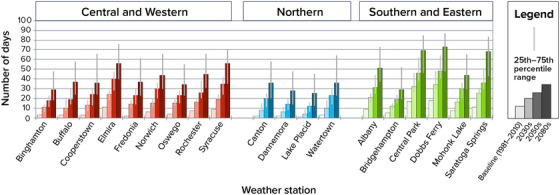
Historical and projected future number of days per year over 90°F in New York State. Columns show the median (50th percentile) modeled results from a blend of the SSP2‐4.5 and SSP5‐8.5 greenhouse gas emissions scenarios, while the thinner lines show the 25th–75th percentile range from this same two‐scenario blend. Data from projections developed for this assessment.^3^

The number of days over 95°F is also projected to increase across New York State. Dobbs Ferry and Central Park are projected to experience the most 95°F days: 13–29 days per year by the 2050s and 18–57 by the 2080s for Dobbs Ferry, and 14–32 days by the 2050s and 17–54 days by the 2080s for Central Park. Lake Placid, the coldest station considered in this analysis, had no days over 95°F during the baseline period but can expect 1–3 days per year by the 2050s and 2–16 days per year by the 2080s.^3^

Multiday heat waves are expected to occur more frequently across New York State in the decades ahead (Figure 2‐8). By the 2080s, all stations are projected to have at least three heat waves each year. Central Park and Dobbs Ferry, which had the most heat waves per year over the 1981–2010 baseline period at an average of 2 per year, are anticipated to experience 5–9 heat waves per year by the 2050s and 6–10 by the 2080s. Saratoga Springs is projected to experience the largest increase in number of heat waves.^3^

**FIGURE 2‐8:**
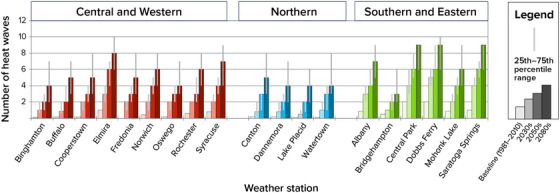
Historical and projected future annual number of heat waves in New York State. Heat waves are defined as 3 or more consecutive days with maximum temperatures at or above 90°F. Columns show the median (50th percentile) modeled results from a blend of the SSP2‐4.5 and SSP5‐8.5 greenhouse gas emissions scenarios, while the thinner lines show the 25th–75th percentile range from this same two‐scenario blend. Instances with no thin gray line protruding above and/or below the top of the shaded column indicate that the 25th and/or 75th percentile values are equal to the median value. Data from projections developed for this assessment.^3^

In addition to becoming more frequent, heat waves are also projected to last longer. From 1981 to 2010, heat waves in Central Park, Dobbs Ferry, and Saratoga Springs lasted an average of 4 days. All three locations are projected to experience heat waves lasting 5−8 days, on average, by the 2080s. Other locations throughout the state are also projected to experience longer heat waves.^3^

### 3.4 Heat index

The human health risks of extreme heat increase when combined with other weather conditions such as high humidity. When humidity is high, the body's natural cooling mechanism—sweating—becomes less effective as sweat evaporates more slowly. New York has a relatively humid climate, like the Eastern United States in general. Thus, it is particularly important for an assessment of climate impacts in New York to consider the additive effect of humidity when examining the danger posed by extreme heat.

Meteorologists characterize the combined stress of temperature and other factors in a few different ways. Two commonly used approaches are:

- **Heat index**: A calculation that combines temperature and relative humidity to report an “apparent temperature.” Heat index values assume shady locations, which means the apparent temperature could feel hotter in direct sunlight. The National Weather Service urges “extreme caution” with heat index values above 90°F, and it classifies values above 103°F as “dangerous” and values above 125°F as “extremely dangerous.” A heat index below the “extreme caution” level can still be dangerous, as studies have found statistically significant increases in adverse health effects when the daily maximum heat index is between 76°F and 90°F, including excess cardiovascular hospitalizations, heat stress, dehydration, and kidney failure.^27^, ^28^
- **Wet‐bulb globe temperature**: A measure of heat stress in direct sunlight that considers temperature, humidity, wind speed, and sun angle and cloud cover (solar radiation). One component of this measurement is the wet‐bulb temperature, which is the temperature read by a thermometer covered in a wet cloth.

Wet‐bulb globe temperature has gained increasing use as an arguably better representation of the heat stress felt by the human body. However, it requires more information than heat index to compute. This assessment focuses on heat index because GCMs can project temperature and relative humidity, whereas the models are less able to project wind speed and cloud cover with confidence. The assessment examines heat index values starting at 85°F in light of the observed health effects described above.

#### 3.4.1 Historical observations

The occurrence of high heat index values has varied substantially by region in New York State, historically. New York City has typically experienced the most days with high heat index values: an average of 38 days per year with a heat index above 85°F and 6 days per year above 95°F, as measured at Central Park during the 1981–2010 baseline period.^3^ During that period, the maximum annual heat index value was 101°F, on average. Another study examined the average daily heat index from 1997 to 2006 at LaGuardia Airport, with an average daily maximum of 80.4 ± 10°F for May through September, 87°F for July, and 86°F for August.^29^ In contrast, Lake Placid experienced an average of only 2 days per year with a heat index above 85°F during the 1981–2010 period, and no days above 95°F.^3^

During heat waves, many regions of New York State, particularly cities, can exceed the “extreme caution” heat index threshold of 90°F, and sometimes even the “dangerous” threshold of 103°F. In July 2001, for example, Rochester achieved a record‐high heat index of 117°F even though the air temperature was only 94°F.^30^

#### 3.4.2 Projections of future change

The number of days with a heat index greater than 85°F, the number of days with a heat index greater than 95°F, and the maximum heat index are expected to increase substantially across all regions of New York State.^4^

By the 2050s, the warmest location modeled, Central Park, is projected to experience 74–93 days per year with a heat index above 85°F and 30–46 days above 95°F.^3^ By the 2080s, those numbers increase to 89–118 days above 85°F and 39–77 days above 95°F.^3^ This is the largest change across all stations modeled. Other cities projected to experience large increases in the number of high heat index days include Albany, Buffalo, and Rochester. The coldest location modeled, Lake Placid, can expect 22–53 days per year with a heat index above 85°F by the 2080s—compared with a historical baseline of 2 days per year—and 2–15 days above 95°F, versus a baseline of 0 days per year.^3^

Projections also show the annual maximum heat index increasing over time throughout New York (Figure 2‐9).^3^ During the 1981–2010 baseline period, all locations had a typical annual maximum heat index value below the 103°F “dangerous” threshold, although Central Park and Dobbs Ferry were close at 101°F and 100°F, respectively. By the 2050s, the projected range is at or above 103°F for all but three stations—and all but Lake Placid are at least 102°F. The 2080s are projected to be even warmer. Central Park and Dobbs Ferry are both projected to experience maximum heat index values between 115°F and 130°F—a range encompassing heat index values classified as “extremely dangerous.” All stations are projected to experience at least a 12°F increase in the maximum heat index by the 2080s, compared with the 1981–2010 baseline. Oswego is projected to experience the largest change.^3^

**FIGURE 2‐9:**
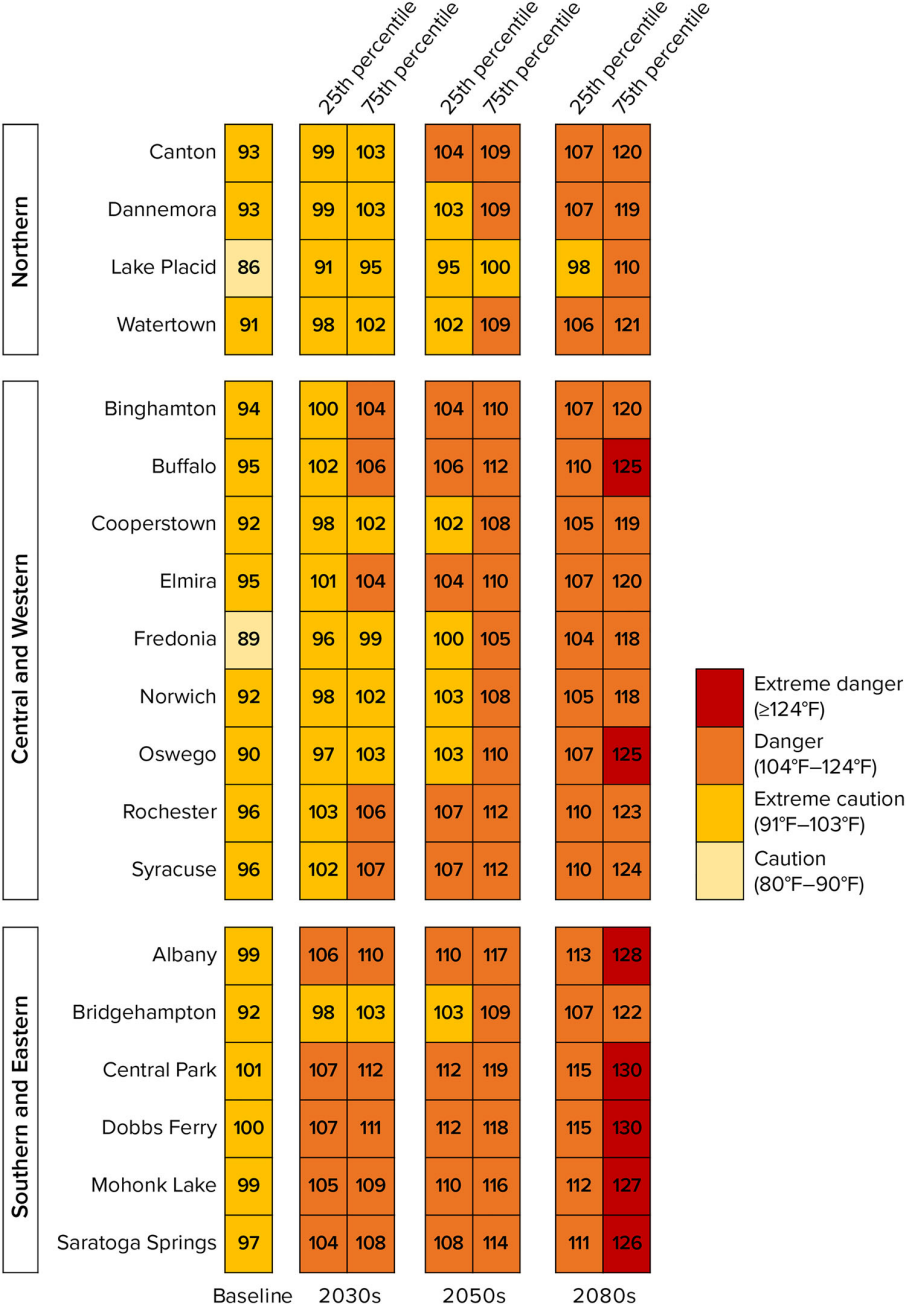
Projected annual maximum heat index in New York State during the 21st century by station. The grid shows 25th and 75th percentile modeled results from a blend of the SSP2‐4.5 and SSP5‐8.5 greenhouse gas emissions scenarios. Data from projections developed for this assessment.^3^

### 3.5 Extreme cold

Like extremely hot days, extremely cold days also occur as a natural part of day‐to‐day variation in weather. Extremely cold weather can pose a danger to the health of people who are unprepared and exposed, and it can also damage infrastructure.^31^ What is considered “extreme cold” varies regionally, as temperatures below 0°F are common during winter in some parts of New York but quite rare in others. Climate change is generally expected to lead to less frequent and less extreme cold spells as temperatures warm worldwide.^1^

#### 3.5.1 Historical observations

Temperatures in New York can drop to negative double digits during winter cold spells. The state's record low temperature of −52°F occurred in the Adirondacks, at Stillwater Reservoir in 1934 and Old Forge in 1979.^32^ Albany, Buffalo, and Rochester have all experienced record lows of −20°F or below.^18^, ^20^, ^21^ Looking at broader patterns, Lake Placid averaged 33 days per year with a minimum temperature below 0°F during the period 1981–2010, and Watertown averaged 21.^3^ Subzero temperatures have occurred less frequently elsewhere—an average of 6 days per year in Binghamton, 3 in Buffalo, and 0.2 days (1 day every 5 years) at Central Park from 1981 to 2010. Over the longer record from 1900 to 2021, Central Park experienced 37 days with a minimum temperature at or below 0°F, but only 9 of those days occurred within the past 60 years.^4^

A review of historical data shows that winter nights are becoming warmer across New York State, with very cold nights (i.e., nights with a temperature below 0°F) occurring less frequently since 1990, compared with most of the 20th century.^13^

#### 3.5.2 Projections of future change

The annual numbers of days below 0°F and days below 32°F are projected to decrease across the state (Figure 2‐10). At Lake Placid, the coldest location included in these projections, the change will be substantial: a projection of only 9–15 days per year below 0°F in the 2050s, and 2–11 in the 2080s, compared with 33 during the 1981–2010 baseline period. Lake Placid is also projected to lose 39–68 days per year below the freezing point (32°F) by the 2080s. At the other end of the spectrum, Central Park, the location with the fewest cold extremes, is also projected to experience fewer very cold days. By the 2050s, Central Park is projected to experience no days below 0°F and 31–48 days per year below 32°F, compared with a baseline of 70. By the 2080s, Central Park is projected to experience only 9–39 days per year below 32°F.

**FIGURE 2‐10:**
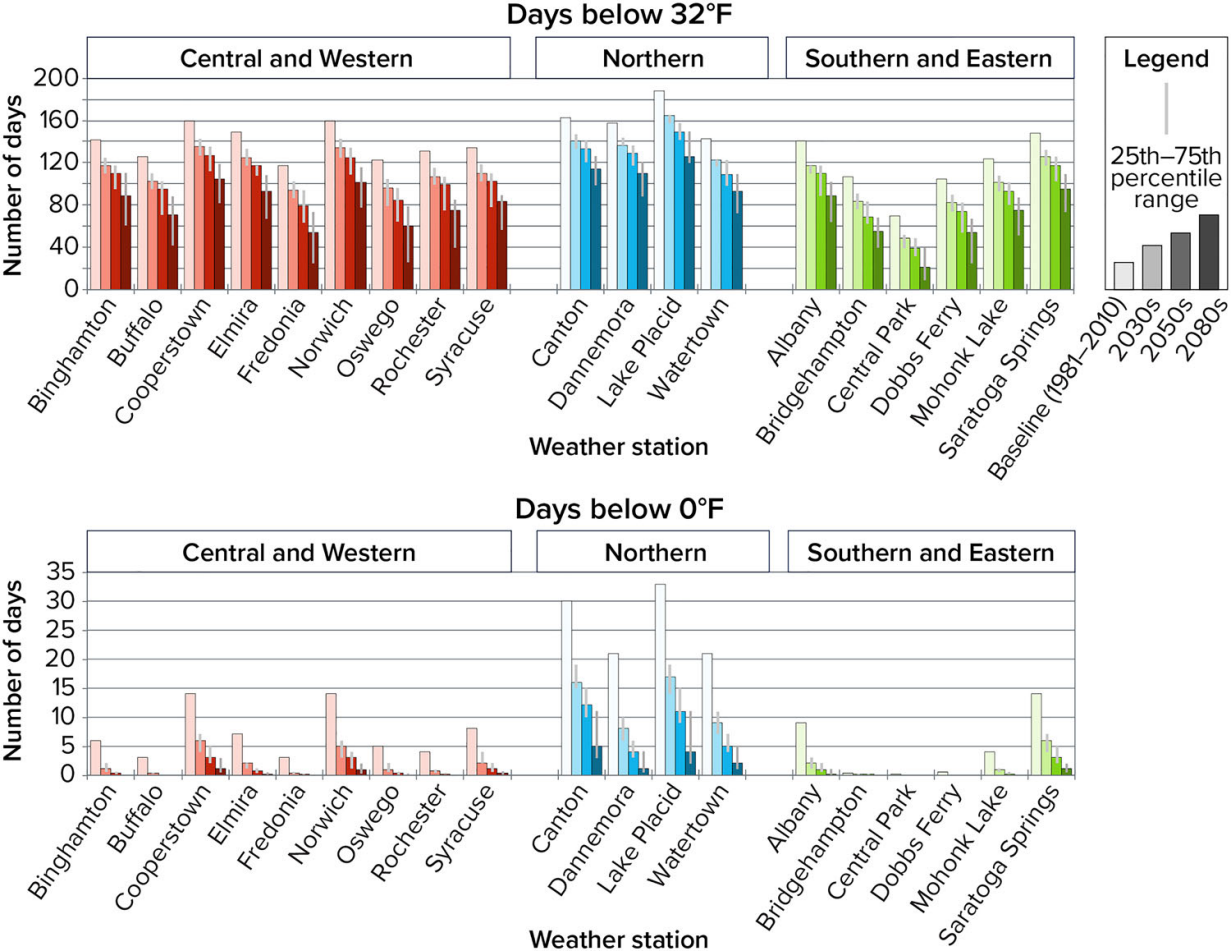
Historical and projected future number of days below 32°F and 0°F in New York State. Columns show the median (50th percentile) modeled results from a blend of the SSP2‐4.5 and SSP5‐8.5 greenhouse gas emissions scenarios, while the thinner lines show the 25th–75th percentile range from this same two‐scenario blend. Data from projections developed for this assessment.^3^

The loss of days below freezing is particularly concerning, as frozen conditions influence animal migration, control the activity and proliferation of pests (e.g., ticks), and affect hydrological cycles, the growing season, and more generally whether precipitation falls as rain or snow. The latter is of great importance to winter recreation and the local economies that depend on it. By the 2080s, the locations modeled for this assessment are projected to lose between 31 (Central Park) and 96 (Oswego) days per year below 32°F relative to the 1981–2010 baseline.^3^ This change essentially represents the frozen season shrinking by 1−3 months.

Some research has suggested that extreme cold air outbreaks connected with the polar vortex, a large area of low pressure that is typically centered on the North Pole, could become more common in places like the northeastern United States as the Arctic warms.^33^, ^34^ However, more recent research suggests that extreme cold associated with polar vortex events is unlikely to increase, as detailed in the projections methodology report.^4^

### 3.6 Heating and cooling degree days

Changes in outdoor temperature will influence the amount of energy that New Yorkers use to heat or cool their homes and other buildings. This relationship is commonly examined using the concept of “degree days.” Heating degree days and cooling degree days are computed by taking each day's daily average temperature and calculating how far it is below or above a common reference point of 65°F, respectively. Heating degree days are used to estimate energy requirements for heating, whereas cooling degree days are used to estimate energy requirements for air conditioning.

#### 3.6.1 Historical observations

With annual average temperatures well below 65°F throughout the state, New York experiences substantially more heating degree days than cooling degree days each year. From 1895 to 2022, New York experienced a long‐term average of 6429 heating degree days and 507 cooling degree days per year (Figure 2‐11). An ordinary least‐squares linear regression over the entire period of record indicates that heating degree days have decreased at a rate of about 8 degree days per year, and cooling degree days have increased at about 2 degree days per year; both results are significant to a 99% confidence level.^14^ Figure 2‐11 suggests that much of the change has been concentrated in the past few decades.

**FIGURE 2‐11:**
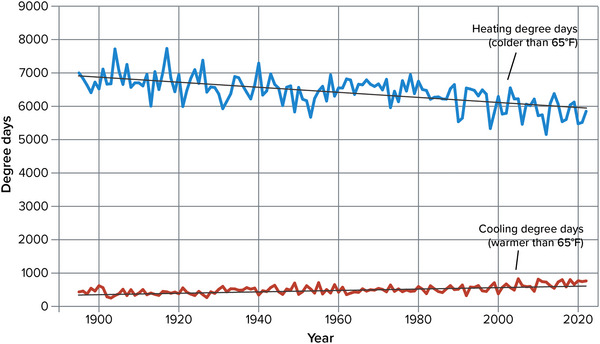
Heating and cooling degree days in New York State, 1895–2022. Daily mean temperatures and annual degree days were calculated for each NOAA climate division as part of NOAA's nClimDiv data set, then averaged across all of New York's climate divisions, weighted by each division's population. With this approach, state heating degree day and cooling degree day numbers more closely reflect the conditions that the average resident of the state would experience. Ordinary least‐squares linear trends are shown as black lines for reference; heating degree days (−7.7 degree days per year, *p* < 0.001), and cooling degree days (+2.0 degree days per year, *p* < 0.001) both have trends significant to a 99% confidence level. Data from NOAA (2023).^14^

#### 3.6.2 Projections of future change

The projections developed for this assessment show agreement across all climate models that heating degree days will decrease in New York over the course of the 21st century, and cooling degree days will increase. The direction of change is consistent for all 19 long‐term weather stations that had sufficient data to downscale degree‐day projections. The range of results across all 19 stations suggests that New Yorkers will experience 1229–3026 fewer heating degree days in the 2080s compared with the 1981–2010 baseline, a 22%−41% decrease. Conversely, New Yorkers are projected to experience 377–1626 more cooling degree days in the 2080s compared with the 1981–2010 baseline, a 69%−528% increase. (Each of the statewide ranges given here comprises the 25th percentile from the location with the least change and the 75th percentile from the location with the most change.) Figure 2‐12 shows projections by decade for six locations in different parts of the state.

**FIGURE 2‐12:**
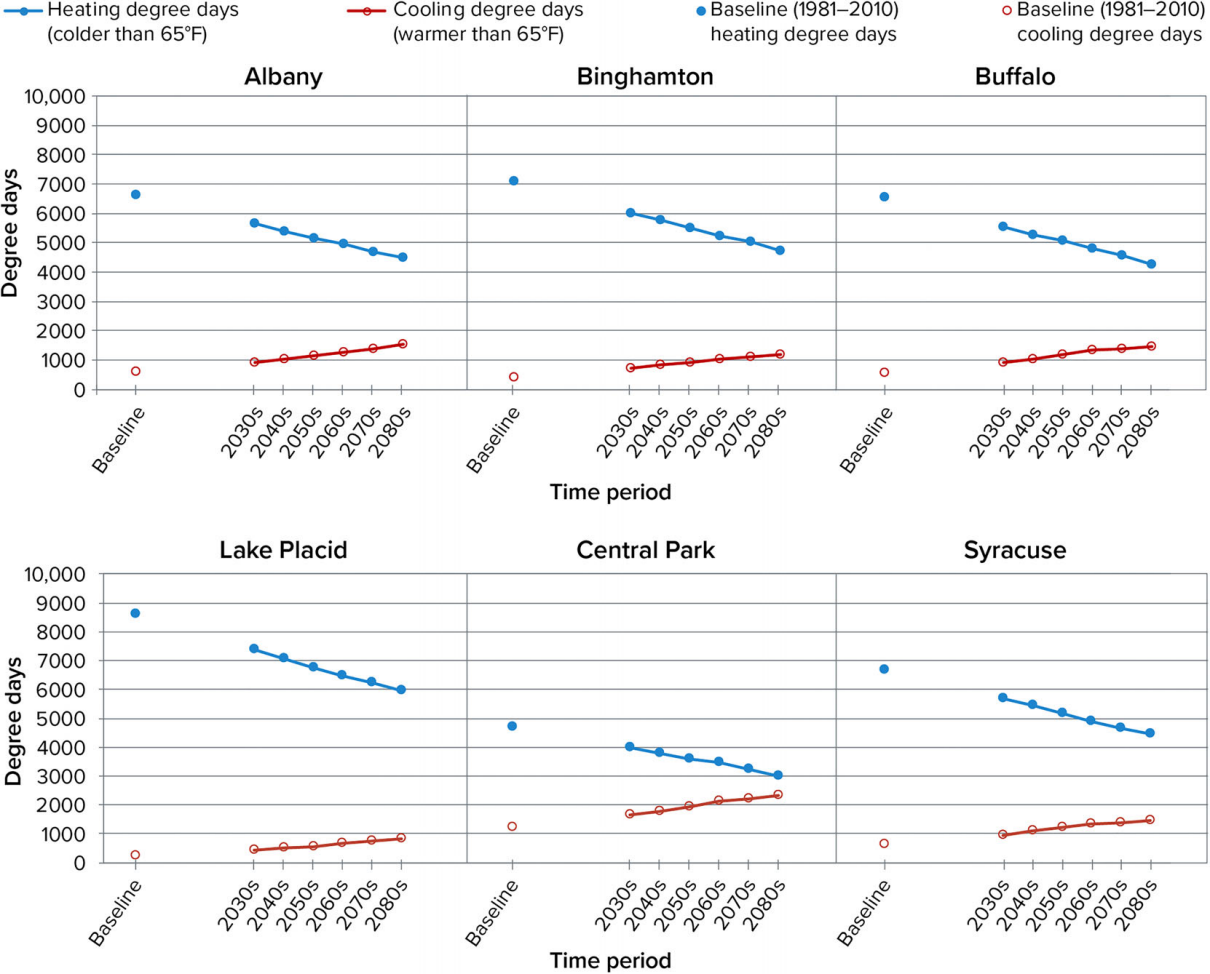
Projected heating and cooling degree days for six representative locations in New York State during the 21st century. The graphs show median (50th percentile) modeled results from a blend of the SSP2‐4.5 and SSP5‐8.5 greenhouse gas emissions scenarios. These six locations were selected to provide geographic and climatological variety within a limited space. Data from projections developed for this assessment.^3^

## 4 PRECIPITATION

### 4.1 Total annual precipitation

Rain and snow are vital to sustaining life in New York. New York is fortunate to be generally “water rich,”^35^ but there are still many human and ecological needs competing for water. Total annual precipitation—inches of rain plus snow converted to its liquid equivalent—offers a broad look at the amount of water available to meet these needs. Tracking and modeling total precipitation can also reveal the potential for excess moisture and its associated challenges, such as flooding. At a global scale, climate change will increase total precipitation as warming accelerates the water cycle, increasing evaporation that then leads to more precipitation. However, the shifting weather patterns associated with climate change will spread the additional precipitation unevenly, causing some parts of the world to receive less precipitation than they did in the past, while others receive substantially more. The northeastern United States is among the regions widely expected to receive more precipitation overall as the Earth warms.^1^

#### 4.1.1 Historical observations

From 1901 to 2022, New York received an average of 41.1 inches of precipitation per year statewide.^14^ Totals vary regionally. New York's wettest areas are those downwind of Lake Erie and Lake Ontario and those near the coast. These areas received an average of approximately 46 inches per year over the baseline period 1981–2010. The west‐central part of the state is relatively drier, receiving 35 inches per year on average over the baseline period 1981–2010.^4^

Annual precipitation across New York State increased at a rate of 0.47 inches per decade from 1901 to 2022 (Figure 2‐13). This change mirrors the trend across the entire northeastern United States over the same period, which was 0.46 inches per decade.^14^ The increase in New York is statistically significant to a 99% confidence level.^4^ Total change has amounted to 10%−20% since 1901.^36^

**FIGURE 2‐13:**
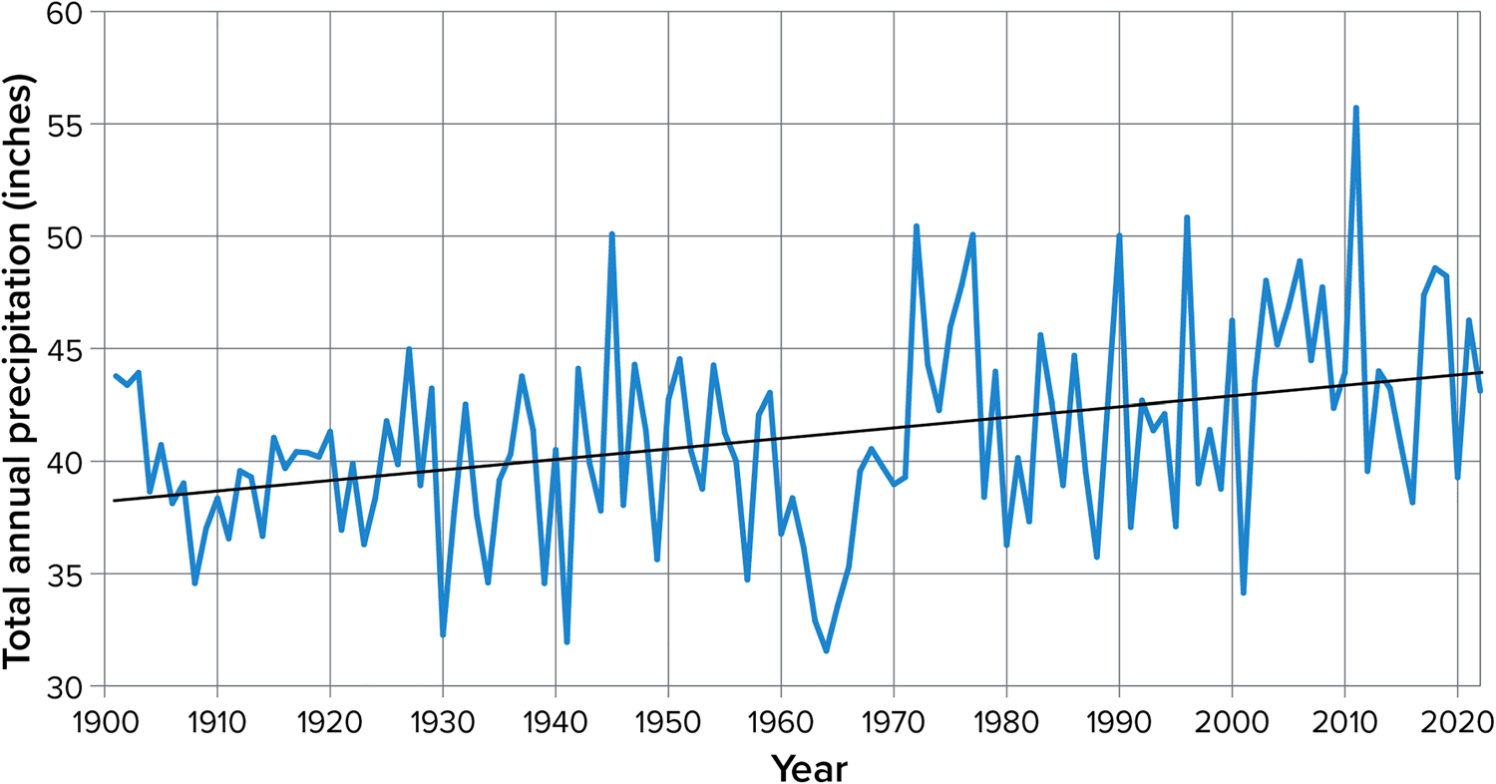
Annual total precipitation in New York State, 1901–2022. The graph shows daily precipitation summed over the entire year, calculated for each NOAA climate division as part of NOAA's nClimDiv data set, and then averaged across all of New York's climate divisions, weighted by each division's area. The black line shows the ordinary least‐squares linear trend (+0.47 inches per decade), which is significant to a 99% confidence level (*p* < 0.001). Data from NOAA (2023).^14^

Of the 27 long‐term weather stations used for this assessment, 19 stations experienced increases in annual precipitation from 1901 to 2020 that were significant at the 95% level; 15 of these increases were also significant at the 99% level. Trends range from an increase of 1.03 inches per decade observed at Oswego to a decline of 0.16 inches per decade (though not statistically significant) at Setauket on Long Island.^4^ Year‐to‐year variation in annual precipitation has also become more pronounced, as evidenced by increases in the standard deviation of annual precipitation at 24 out of 27 stations.^4^

#### 4.1.2 Projections of future change

Annual precipitation is projected to increase progressively in all New York State climate regions through the late 21st century (Figure 2‐14). Across the state, precipitation is projected to increase by approximately 1%−8% by the 2030s, 2%−12% by the 2050s, and 6%−17% by the 2080s, relative to a 1981–2010 baseline.^3^

**FIGURE 2‐14:**
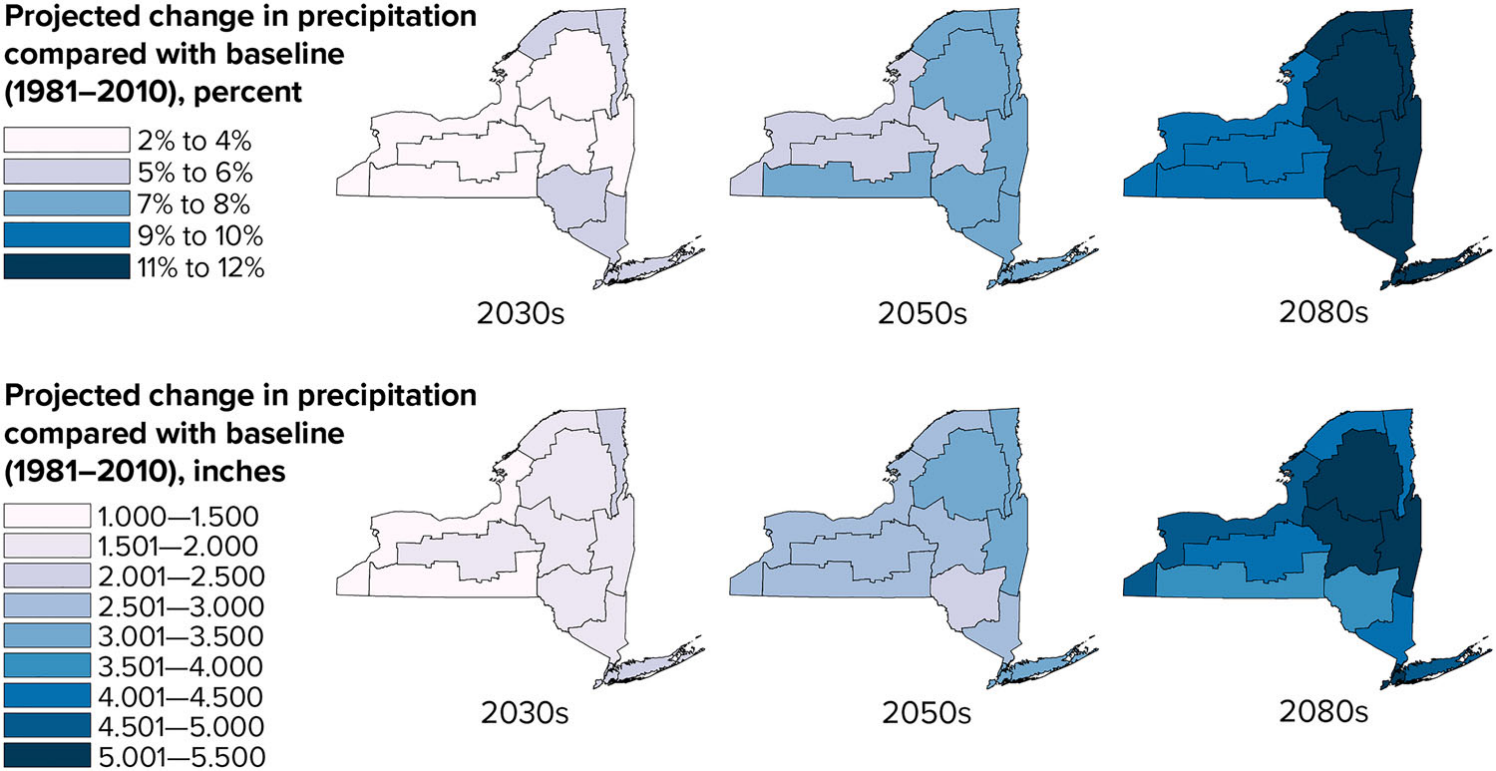
Projected annual precipitation in New York State during the 21st century, relative to the 1981–2010 baseline. The maps show median (50th percentile) modeled results from a blend of the SSP2‐4.5 and SSP5‐8.5 greenhouse gas emissions scenarios. Data from projections developed for this assessment.^3^

The largest percentage increases in annual precipitation are projected for the New York City, Catskills, and South Hudson assessment regions.^3^ For example, annual precipitation in New York City is projected to increase by 4%−11% by the 2050s and by 7%−17% by the 2080s, relative to the 1981–2010 baseline of 49.9 inches per year.^3^ New York City will continue to be the wettest assessment region, and the Central/Finger Lakes region will continue to be the driest.

### 4.2 Seasonal precipitation

The importance of precipitation as a climate variable is a matter of not just how much precipitation falls, but also when it falls. The timing of precipitation has critical implications for water availability to support base streamflow and human uses throughout the year. Precipitation in specific seasons is critical to certain sectors of the economy, such as agriculture and winter recreation. Too much precipitation in a particular season can contribute to flooding, saturated farm fields that inhibit planting,^37^ and other challenges.

#### 4.2.1 Historical observations

Precipitation in New York State is fairly evenly distributed throughout the year, with an average of 8.63 inches in winter, 10.02 inches in spring, 11.59 inches in summer, and 10.79 inches in fall over the 1901–2022 period (Figure 2‐15).^14^ Precipitation in inland New York typically peaks in intensity in the summer, while coastal areas tend to receive the most precipitation during the late spring and early fall.^38^

**FIGURE 2‐15:**
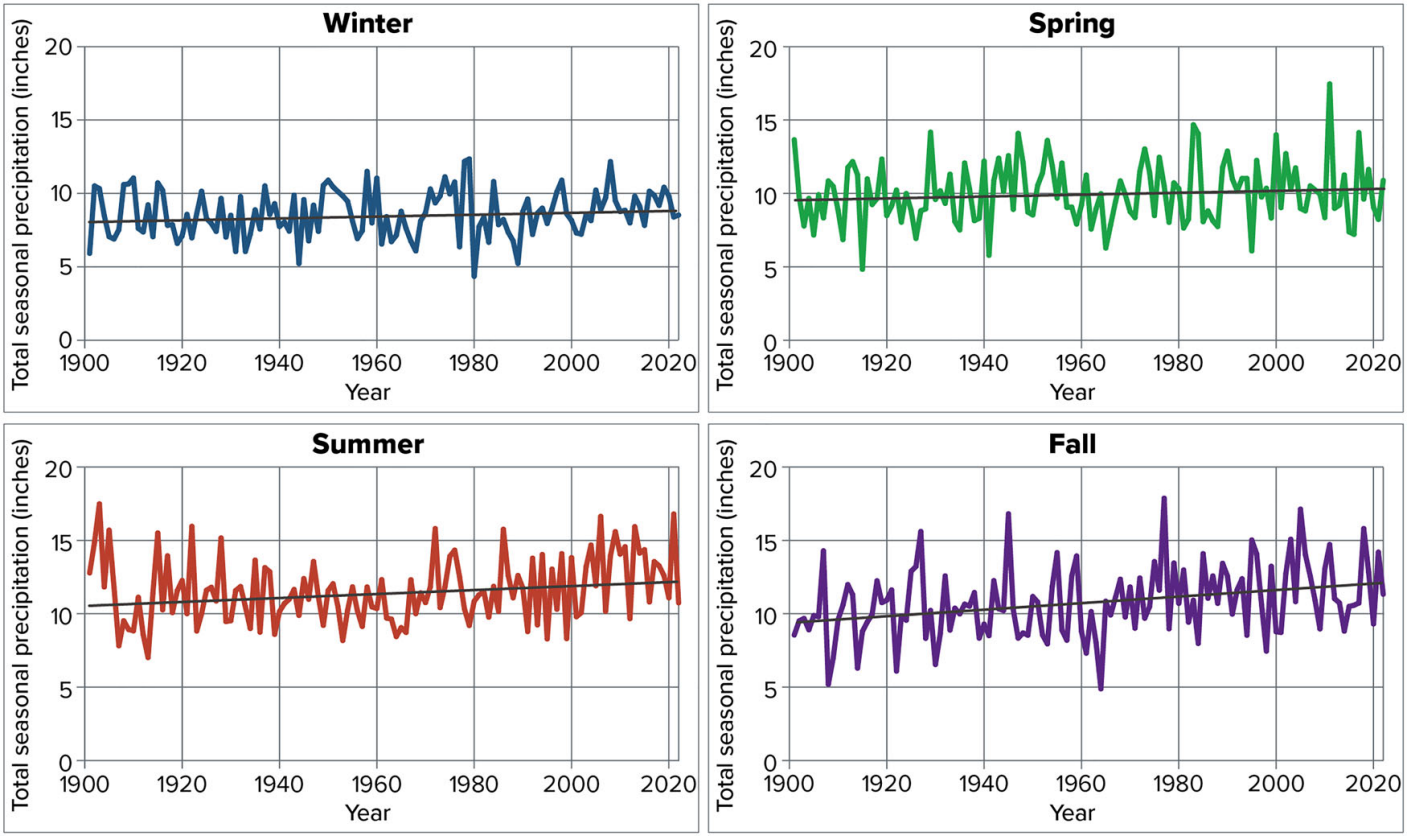
Total precipitation by season in New York State, 1901–2022. Daily precipitation has been summed over each season, calculated for each NOAA climate division as part of NOAA's nClimDiv data set, and then averaged across all of New York's climate divisions, weighted by each division's area. Seasons are defined meteorologically: spring is March–May, summer is June–August, fall is September–November, and winter is December–February (plotted here at the year when the winter season ended; e.g., December 2021–February 2022 is plotted as 2022). Ordinary least‐squares linear trends are shown as black lines for reference; summer (+0.13 inches per decade, *p* = 0.022) and fall (+0.22 inches per decade, *p* < 0.001) changes are statistically significant to a 95% confidence level, while winter and spring are not. Data from NOAA (2023).^14^

Precipitation in New York has increased in some seasons since the early 20th century. From 1901 to 2022, fall precipitation increased the most, at a rate of 0.22 inches per decade.^14^ Summer precipitation increased at a rate of 0.13 inches per decade.^14^ These long‐term average rates of change are based on ordinary least‐squares linear regression, and both are significant to a 95% confidence level. Winter and spring precipitation did not change significantly.^14^

#### 4.2.2 Projections of future change

Precipitation is expected to increase in winter and spring across all of New York's climate regions throughout the 21st century (Figure 2‐16). This finding is consistent with previous climate projections for New York.^39^ Future trends in summer and fall precipitation are less clear.

**FIGURE 2‐16:**
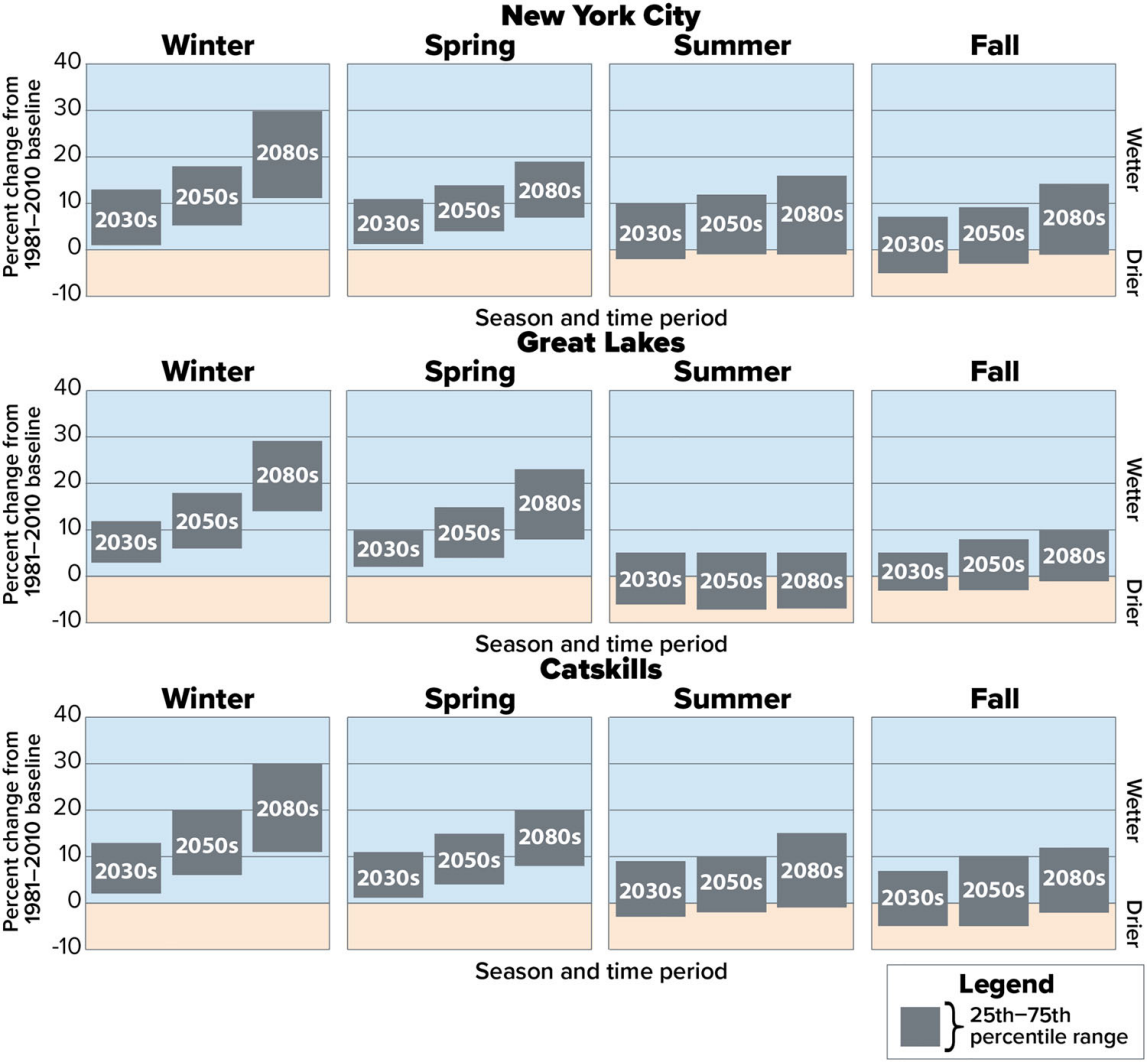
Projected changes in seasonal precipitation in New York City, the Great Lakes, and the Catskills assessment regions in the 21st century, as a percentage change from the 1981–2010 baseline. These three regions were selected to provide a geographically diverse sample of assessment regions. The gray bars show the range from the 25th to the 75th percentile using a blend of the SSP2‐4.5 and SSP5‐8.5 greenhouse gas emissions scenarios. A negative percentage change indicates that the season is projected to become drier, while a positive percentage change indicates that the season is projected to become wetter. Data from projections developed for this assessment.^3^

Total winter precipitation is projected to increase 5%−21% by the 2050s and 11%−31% by the 2080s, compared with the 1981–2010 baseline. Spring precipitation is projected to increase 3%−17% by the 2050s and 6%−23% by the 2080s. The wide ranges listed here cover from the 25th percentile of the lowest assessment region to the 75th percentile of the highest region. All regions of the state are projected to experience an increase in both winter and spring, and the projected percentage changes through the 2080s are fairly comparable across regions, with no large regional differences or outliers.^3^

Projections of summer and fall precipitation are less definitive, with some climate models projecting an increase in precipitation and some projecting a decline throughout the 21st century. Across assessment regions, total precipitation in summer is projected to change between −7% and 12% by the 2050s, and −7% to 20% by the 2080s. Precipitation in fall is projected to change −5% to 10% by the 2050s, and −2% to 14% by the 2080s.^3^

### 4.3 Heavy precipitation

Heavy precipitation refers to a large amount of rain or snow falling during a short period of time, such as a single day or even a single hour. Heavy precipitation events can lead to devastating flooding and damage to infrastructure and ecosystems as excess water overwhelms the ability of the natural landscape and the built environment to absorb it or carry it away.

Increases in the frequency and intensity of heavy precipitation have been observed worldwide and attributed to human‐induced climate change.^1^ This observation is consistent with the expectation that warmer air, warmer bodies of water, and increased evaporation will contribute to the formation of more intense storms. This trend is expected to continue.^1^

Meteorologists and climatologists characterize heavy precipitation in various ways for different needs, including different time frames and different types of thresholds: fixed (precipitation exceeding a certain number of inches) or probabilistic (return period or likelihood of occurrence based on the historical record, reflecting locational differences in what is considered “extreme”). The team for this assessment developed projections of heavy precipitation with respect to three fixed thresholds over a 1‐day period: days with more than 1, 2, and 4 inches of precipitation.

#### 4.3.1 Historical observations

According to the Northeast Regional Climate Center (NRCC), the frequency of 2‐inch precipitation events has increased since the 1950s across New York and New England.^40^ New York has experienced an above average number of 2‐inch precipitation events since 1995, with the frequency of 2‐inch precipitation events peaking from 2010 to 2014.^39^ Across New York and New England, storms previously considered once in 100‐year events (an event with a 1% chance of occurring in any given year) are also becoming more frequent, occurring nearly twice as often as expected in recent years.^40^ What constitutes a 100‐year event ranges from a 4‐ to 5.5‐inch storm in Northern and Western New York to an 8‐inch storm in New York City and on Long Island.^40^

Several of the weather stations examined elsewhere in this assessment had sufficient data to analyze whether the annual number of heavy precipitation events has changed over time. These weather stations had precipitation data as early as 1869 for Central Park, 1894 for Elmira, and the 1920s to 1950s for the other sites. Seven of the nine weather stations statistically analyzed had significant long‐term increases in the number of days per year with precipitation greater than 1 inch (*p* < 0.05).^12^ Two of the nine weather stations statistically analyzed, Oswego and Central Park, had a significant increase over time in the number of days per year with precipitation greater than 2 inches (*p* < 0.05). None of the sites analyzed statistically had a significant long‐term linear trend in the number of days per year with precipitation greater than 4 inches, which remains an exceedingly rare event (all *p* values were greater than 0.05).

While extreme rainfall is more common in coastal parts of New York that are periodically affected by tropical storms and hurricanes, extreme rainfall is not exclusive to tropical cyclones. For example, the state's 24‐h precipitation record (13.57 inches) was set at Islip, Long Island, during a nontropical storm in August 2014.^39^

Extreme events often drive interannual differences in annual precipitation. For example, the record for the most annual precipitation in New York State was set in 2011. Back‐to‐back extreme precipitation events caused by Hurricane Irene and Tropical Storm Lee are recognized as drivers that led to the precipitation record being broken. Hurricane Irene, which occurred during late August 2011, delivered 7 inches of rain to Eastern New York and more than 18 inches at certain locations in the Catskills. Tropical Storm Lee, which occurred in early September, delivered more than 12 inches to the Susquehanna River basin.^39^

Over the 1981–2010 baseline period used for this assessment's modeling, the four weather stations closest to the coast (Bridgehampton, Central Park, Dobbs Ferry, and Mohonk Lake) each experienced an average of 14 or 15 days per year with precipitation over 1 inch. All four stations also experienced 3 days per year with precipitation over 2 inches. Extreme precipitation events were much less frequent throughout the rest of the state, with stations averaging between 5 and 10 days per year with precipitation greater than 1 inch and between 0.3 and 1.0 days per year with precipitation greater than 2 inches. Most stations did not experience any precipitation events with more than 4 inches in 1 day during the baseline period. Bridgehampton experienced the most such events, at a rate of three every 10 years.^3^

#### 4.3.2 Projections of future change

Days with more than 1 inch and days with more than 2 inches of precipitation (Figure 2‐17) are projected to become more frequent at all 19 stations included in this analysis, based on the median results for the blended emissions scenarios used for this assessment.^3^ By the 2080s, days with more than 4 inches of precipitation are also projected to become more frequent at 12 of the 19 stations considered in this analysis.^3^

**FIGURE 2‐17:**
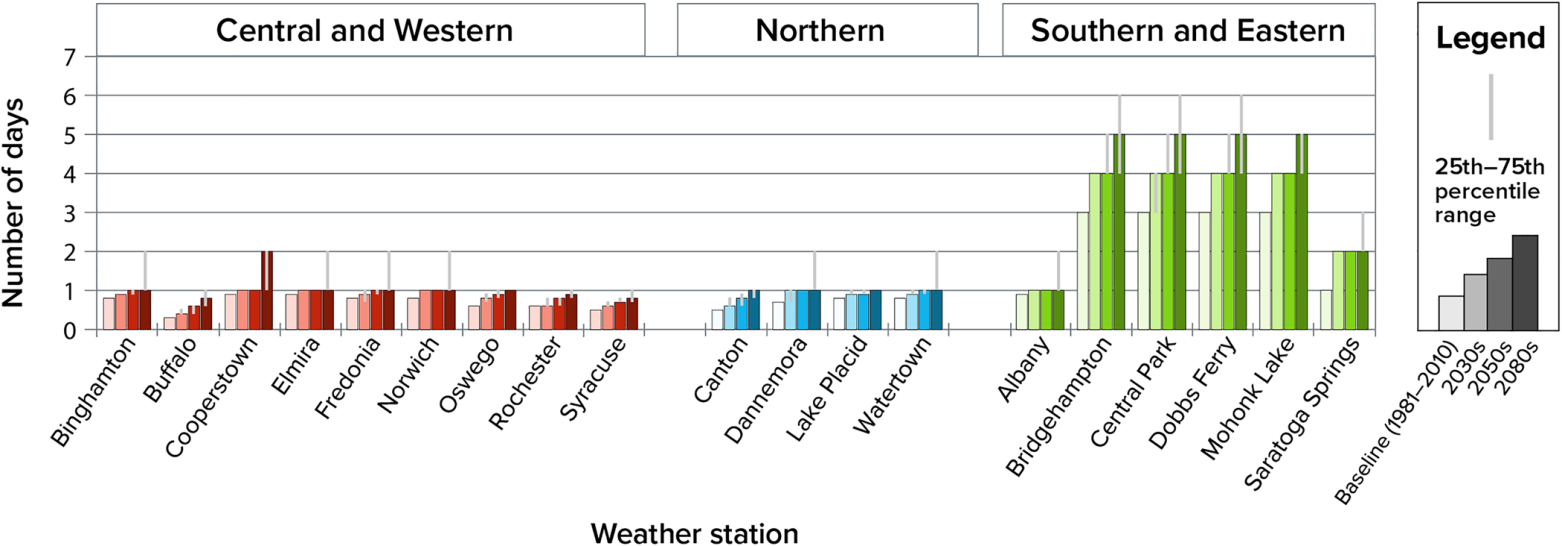
Historical and projected future number of days per year with more than 2 inches of precipitation in New York State. The shaded bars show the median (50th percentile) modeled results from a blend of the SSP2‐4.5 and SSP5‐8.5 greenhouse gas emissions scenarios. The gray bars show the range from the 25th−75th percentile modeled results. Instances with no thin gray line protruding above and/or below the top of the shaded column indicate that the 25th and/or 75th percentile values are equal to the median value. Data from projections developed for this assessment.^3^

From the 1981–2010 baseline to mid‐century (2050s), the largest increases in 2‐inch precipitation days are projected at Saratoga Springs, Bridgehampton, Central Park, Dobbs Ferry, and Mohonk Lake. Ten out of 19 stations are projected to experience 4‐inch precipitation days at least once per decade by the 2050s, increasing to 16 out of 19 stations by the 2080s. Bridgehampton and Mohonk Lake are projected to experience 4‐inch days five times per decade by the 2080s—roughly twice as often as during the baseline period.^3^

### 4.4 Snowfall and snow cover

Whether precipitation falls as rain or snow, and how long snow persists on the ground, matters for many reasons. Snow can pose challenges for infrastructure, mobility, and safety, yet it is also vital for many cherished winter activities and the local economies they sustain. Many types of ecosystems are adapted to winter snow cover, and the timing and amount of snowmelt at the end of the winter influence water availability and soil moisture later in the year. From a global perspective, snow reflects more sunlight back to space than bare ground or water, so an overall reduction in snow cover leads to more solar energy being absorbed by the Earth's surface, which further increases warming.

A warmer climate generally means that more winter precipitation could fall as rain instead of snow, and the snow that does fall will likely remain on the ground for less time as warmer air promotes more rapid melting. However, some areas could receive more snow if winter precipitation increases (Section 4.2), while the temperature remains below freezing. This is particularly likely over the next few decades in parts of New York dominated by lake‐effect snow, as Section 4.4.2 describes.

#### 4.4.1 Historical observations

Snowfall varies considerably across New York State. Long Island and New York City have historically received the least snow, at about 30 inches per year, while annual totals immediately east of Lake Erie and Lake Ontario often reach or exceed 150 inches due to lake‐effect storms.^4^

Some changes in snowfall have been observed over time:

- A review of annual snowfall totals from the 19 weather stations with long‐term data used for this assessment found that four stations had a significant linear trend over their full period of record (*p* < 0.05, indicating significance to a 95% confidence level).^12^ One—Lake Placid—has experienced a long‐term decrease in snowfall. The other three—Fredonia, Rochester, and Oswego—have experienced a long‐term increase. The latter observation is generally consistent with a 2003 study that found an increase in lake‐effect snowfall downwind from the Great Lakes since 1950.^41^ Three additional stations had results that were significant to a 95% level using one regression test but not another: Watertown (increase), Dannemora (decrease), and Central Park (decrease).^12^
- An earlier analysis of data from 1930 to 2007 published by EPA found a long‐term snowfall increase of 0.5% per year in Buffalo and a mix of smaller changes (some increasing, some decreasing) at six other locations examined.^42^ This was a screening‐level analysis that relied on linear regressions and did not report statistical significance.
- The EPA report mentioned above also examined long‐term trends in the snow‐to‐precipitation ratio, representing the proportion of total winter season precipitation that falls in the form of snow. Syracuse and Binghamton had statistically significant decreases in the snow‐to‐precipitation ratio from 1949 to 2020 (trend via Kendall's Tau method; significance to a 95% confidence level), while 10 other stations across New York did not have a statistically significant change.^42^

In terms of snow depth, several of the weather stations used elsewhere in this assessment had sufficient data to analyze whether maximum annual snow depth has changed over time, dating back to 1913 for Central Park and as early as the 1920s for other sites. There has been much year‐to‐year variability, however, and none of the sites analyzed statistically had a significant long‐term linear trend (all *p* values were greater than 0.2).^12^

Additional observations point to a broader regional pattern of declining snow cover (areal extent) and snowpack (seasonal accumulation):

- Some of the best long‐term data on snow conditions in the Northeast come from the Hubbard Brook Ecosystem Study, a long‐running research program at the Hubbard Brook Experimental Forest in the White Mountains of New Hampshire. Hubbard Brook has experienced statistically significant declines in days of snow cover, snow depth, and the amount of water held in the snowpack since the mid‐1950s.^43^
- Daily satellite imagery of the extent of the globe covered by snow shows that snow cover in North America (excluding Greenland) has held steady in winter but declined substantially during spring from 1972 to 2020.^44^ During this time, the portion of the year when snow covered the ground decreased by an average of nearly 2 weeks across the United States.^44^
- At five stream gauging sites in the Catskills region, the timing of peak snowmelt (as measured by the winter‐spring center of volume) shifted earlier by 5–10 days between 1940 and 2018.^45^

The New York State Mesonet, coordinated by the University at Albany, includes a Snow Network with 20 monitoring stations, largely in the Adirondacks, the Catskills, and the Tug Hill region.^46^ Snowpack in these regions supports winter recreation, streamflow, and drinking water supplies (particularly in the Catskills, where snowmelt feeds the water supplies for New York City and Philadelphia). Each station has automated equipment that measures and reports snow depth at 5‐min intervals and snow water equivalent at 6‐h intervals.^46^ Mesonet stations began to come online in 2015, so they have not yet collected enough years of data to reveal climatologically meaningful trends in snowpack depth or persistence. Nonetheless, continued monitoring will provide important data to characterize changes in snow accumulation in the future. Further analysis could also consider longer‐term snow depth data collected at 2‐week intervals by the New York State Cooperative Snow Survey.^47^

#### 4.4.2 Projections of future change

Snowfall, depth, extent, and water equivalent are projected to decrease across New York State.^16^ The snow season is projected to become compressed, with more precipitation falling as rain, including some lake‐effect precipitation.^48^

Using two CMIP5 models, a 2015 study^48^ produced dynamically downscaled projections of snow across the Great Lakes basin under the very high emissions scenario (at the time, denoted as RCP8.5). They projected that by 2080–2099, parts of New York mapped in their study will lose 20–67 inches (50–170 centimeters) in annual snowfall and 10–25 days per year with snowfall, compared with the period 1980–1999.^48^

Models suggest that over the next few decades, warmer water and decreased ice cover on the Great Lakes are likely to increase lake‐effect snow.^49^ As the state continues to warm, however, the additional precipitation caused by warmer lake conditions will increasingly fall as rain rather than snow.^49^ This projection is consistent with modeling indicating a reduction in the frequency of heavy lake‐effect snowstorms throughout most of the Great Lakes basin by the late 21st century.^48^

## 5 EXTREME EVENTS

This section presents information about various types of severe storms, storm surge, drought, and wildfires. Other types of extreme events are covered elsewhere in this chapter, including temperature extremes (Sections 3.3 and 3.4), heavy precipitation events (Section 4.3), coastal and inland flooding (Sections 6.2 and 7.3), and seiches (Section 7.5).

### 5.1 Severe storms

New York has suffered several memorably destructive storms in recent years, including Hurricane Irene and Tropical Storm Lee in 2011, Superstorm Sandy in 2012, the remnants of Hurricane Ida in 2021, and the historic snowstorms that hit Western New York in November and December 2022. These examples illustrate several of the types of major storms that can occur in New York, which include:

- Tropical cyclones (hurricanes and tropical storms), which generally occur between July and October and can bring heavy precipitation, high winds, and coastal storm surge as the wind pushes water toward the shore.^50^
- Extratropical cyclones (including nor'easters), which tend to occur during cooler months and can bring high winds, heavy rain or snow, flooding, and wave action along the shore that can persist through several tide cycles.^50^ Nor'easters present a range of risks to the state, but particularly to coastal areas, including extremely heavy precipitation (inland flooding), strong winds, and coastal erosion and storm‐surge‐associated flooding. In addition, nor'easters are often followed by intense cold and damaging winds as storm systems exit the region to the northeast.
- Other heavy snow and ice storms, including storms strengthened by the lake effect over Lakes Erie and Ontario.
- Thunderstorms.
- Tornadoes, derechos (intense windstorms), and other events that are rarer in New York than the other types of storms listed above but do occur on occasion.

#### 5.1.1 Historical observations

Between 1851 and 2022, 15 cyclones hit New York at hurricane strength,^51^ plus many more at tropical storm strength. The state has also experienced frequent nor'easters, lake‐effect blizzards, and other storms. Coastal flooding in New York has historically been caused by storm surge associated with tropical and extratropical cyclones, with peak historical storm surges recorded in the years 1788, 1821, 1960, and 2012.^52^

Several of the recent storms listed above broke records. The December 2022 blizzard set snowfall records in the Buffalo area, for example, while Tropical Storm Lee caused record flooding in the Binghamton area and Superstorm Sandy caused unprecedented coastal flooding and destruction in New York City and on Long Island.^53^, ^54^, ^55^ Sandy's storm surge traveled up the Hudson River all the way to Troy.^53^ Moreover, five of the top 10 largest snowstorms in New York City (as measured in Central Park) have occurred since 2010.^4^ Specific examples like these are not proof alone that severe storms have become substantially more frequent or more intense on the whole in New York State. However, the observed increases in air temperature (Section 3.1), ocean temperature (Section 6.1), and Great Lakes water temperature (Section 7.1) over the past century are indicative of conditions that favor the formation of stronger storms. Increased storm activity in general would be consistent with the observed increase in heavy precipitation events in New York (Section 4.3). The North Atlantic basin overall has experienced an increase in the aggregate intensity of tropical cyclones since the mid‐1990s, but trends in frequency and longer‐term trends in intensity are harder to discern.^56^, ^57^

Coastal storms are also becoming more destructive as a result of sea level rise. As the average sea level rises steadily at all of New York's measurement locations (Section 6.2), it creates a higher base elevation onto which storm surge is added. A 2021 study attributed about $8.1 billion of Superstorm Sandy's economic damages (13% of total damages) to the sea level rise associated with climate change.^58^

#### 5.1.2 Projections of future change

Projected changes in the frequency and severity of storms in New York State depend on the type of storm. Some types and aspects of severe storms can be projected with reasonable certainty, while other types and aspects remain uncertain.^59^, ^60^ Highlights from the literature include:

- **Heavy rain and snowstorms**: As described in Section 4.3, the projections developed for this assessment show with high confidence that precipitation in New York will increasingly fall in more intense events as the climate warms.
- **Tropical cyclones**: Historical trends and climate model projections generally support the idea that tropical cyclone hazards—specifically winds and coastal and inland flooding—will increase over New York State over the course of the 21st century.^4^ However, there are many dimensions to the study of tropical cyclones, and some are understood with more certainty than others. The scientific literature suggests that the total number of tropical cyclones on average worldwide could stay the same or perhaps decrease slightly,^61^, ^62^ but that the storms that do form will be stronger, with more intense wind and rain.^63^ The literature reports increasing confidence that intense tropical cyclones of category 3 and higher will become more frequent in the North Atlantic, and very high confidence that average rates of precipitation associated with tropical cyclones will increase.^64^ Other studies have examined potential changes in storm tracks, with implications for how many storms make landfall in New York. Some historical data and projections show maximum hurricane intensity shifting northward, which might be assumed to increase the chances of a strong hurricane reaching the state in the future.^61^ However, a 2023 study projected that the number of hurricanes making landfall along the northeastern U.S. coast (including New York) is likely to decrease over the remainder of this century, while the southeastern Atlantic coast and the Gulf coast will experience an increase.^65^ That study examined the SSP5‐8.5 very high emissions scenario using eight CMIP6 GCMs along with hurricane and stationary wave models, and it attributed the projected shifts in landfall primarily to changes in large‐scale wind patterns known as steering flow.^65^ One 2022 study looked at forward velocity—how quickly storms move along their paths—and projected an increase in the likelihood of storms approaching New York from the east at slower speeds.^63^ Slower movement along the storm track could exacerbate damage, as seen during Hurricane Harvey in 2017, which brought record‐breaking rainfall and flooding as it lingered over the Houston, Texas area for several days.
- **Extratropical cyclones**: Future changes in extratropical storms are difficult to project. Several studies have projected a decrease in the total number of extratropical cyclones over North America and the Western Atlantic, but there is a possibility of an increase in storm intensity and precipitation on the East Coast.^66^ Moreover, projections from 10 CMIP5 models suggest that East Coast extratropical cyclones could become more intense and 5%−25% wetter on the whole in the future.^66^ Whether winter nor'easters produce rain or snow will depend on air temperature, so more could be expected to deliver rain.^4^
- **Thunderstorms and lightning**: Severe convective storms (i.e., thunderstorms) are generally expected to occur more frequently because of an overall increase in atmospheric conditions that promote the development of such storms.^67^, ^68^, ^69^ This projection is for the contiguous United States as a whole, but one 2007 study examined specific cities and projected that New York City will experience more than twice as many days favorable to thunderstorm formation in 2072–2099 compared with 1962–1989.^67^ This 2007 study used the A2 emissions scenario, which comes from an older set of scenarios and involves warming close to, but not quite as high as, the SSP5‐8.5 very high emissions scenario.
  Some studies have projected that lightning strikes will increase as a function of global average temperature^70^, ^71^ and that the frequency of lightning strikes will increase in certain areas of New York State during the 21st century.^72^ However, other research suggests the possibility of a decrease.^73^ Thus, future trends in lightning remain uncertain.
- **Ice storms**: Evidence suggesting increased frequency or severity of ice storms is limited. One 2011 study modeled the possible effects of climate change on the occurrence of severe freezing rain events in eastern Canada and found a high degree of variation.^74^ They projected increases in ice storm occurrences in some regions during some seasons, and decreases in others.^74^ For portions of southern Ontario closest to New York, the total number of freezing rain events during winter could increase by 35%−80% by the period 2081–2100.^74^

Projections of other types and aspects of severe storm events, including tornadoes, hail, and derechos, are not readily available.^4^

### 5.2 Drought

The USGCRP's glossary defines drought as “An exceptional period of water shortage for existing ecosystems and the human population (due to low rainfall, high temperature and/or wind).”^75^ However, a more nuanced view recognizes a hierarchy of different types of drought, with “meteorological drought” describing a precipitation deficit (essentially the general definition given above), “agricultural drought” describing a soil moisture deficit, and “hydrological drought” describing a deficit in runoff.^76^ Drought can be measured over timescales ranging from weeks to years, and characterized using a variety of methods that range from simple precipitation‐based indices to more sophisticated indices that incorporate variables such as relative humidity, potential evapotranspiration, soil moisture, and streamflow. These indices typically define drought in relation to a particular location's “normal” or long‐term average conditions. This means that even a relatively water‐rich area such as New York can experience periods classified as drought.

New York State has not experienced drought impacts with the intensity and duration that often dominate headlines in other parts of the country, such as California and the Southwest. Nonetheless, the state has experienced—and will continue to experience—periods of drought.

#### 5.2.1 Historical observations

New York has fluctuated between periods of relative wetness and drought throughout the historical record. Figure 2‐18 shows this pattern using the Palmer Drought Severity Index (PDSI). The PDSI has been used widely for many years in part because it is relatively simple to compute from available precipitation and temperature data. Other drought indices such as the Standardized Precipitation Index and Standardized Precipitation Evapotranspiration Index (SPEI) show a similar long‐term pattern.^77^, ^78^

**FIGURE 2‐18:**
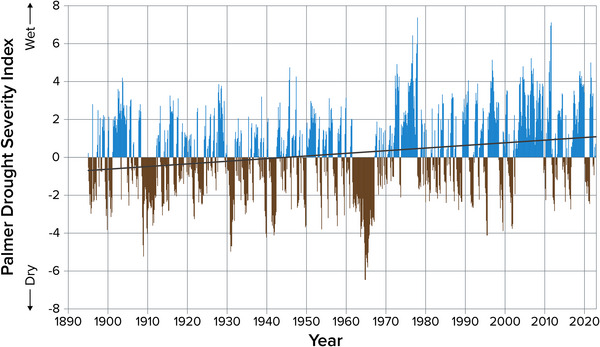
Monthly drought conditions in New York State according to the Palmer Drought Severity Index, 1895–2022. Positive values represent wetter‐than‐average conditions, while negative values represent drier‐than‐average conditions. A value between −2 and −3 indicates moderate drought, −3 to −4 is severe drought, and −4 or below indicates extreme drought. The black line shows the ordinary least‐squares linear trend (+0.013 PDSI units per year), which is significant to a 99% confidence level (*p* < 0.001). Data from NOAA (2023)^14^; index explanation from EPA (2021).^79^

Linear regression (ordinary least‐squares) of the NOAA data^14^ shown in Figure 2‐18 shows a modest, statistically significant (*p* < 0.05) increase in wetness at a rate of +0.013 PDSI units per year from 1895 to 2022. An EPA analysis of trends in 5‐year SPEI values from 1900 to 2020 also found statistically significant increases in wetness across all parts of New York except for Long Island, which had no significant change.^79^

Within the observed record, one particular multiyear drought in the 1960s stands out for its intensity and duration. This dry period is identified as the “drought of record” in many locations in New York State. Supplemental data from tree ring observations in very old trees suggest that the 1960s drought is representative of the most severe droughts in at least the last 500 years, if not longer.^80^, ^81^ Since the 1960s drought, a wetter period has ensued, sometimes referred to as an epic pluvial, with increasing trends in precipitation and minimum streamflow.^81^, ^82^, ^83^, ^84^ However, shorter periods of drought have continued to occur, including recent summer droughts that have had a particular impact on New York's agriculture sector.^37^

#### 5.2.2 Projections of future change

New projections of future drought were not developed as part of this assessment. However, the latest projections of temperature and precipitation at monthly to annual timescales shed some light on potential future changes in meteorological and agricultural drought conditions.

Projected changes depend on the timescale. The projected increases in total annual precipitation in New York (Section 4.1) suggest that the risk of extended, **multiyear droughts** will not increase.^4^ However, **shorter‐term seasonal droughts** lasting from weeks to months could increase for a few reasons:

- Changes in the distribution of precipitation toward larger events, as described in Section 4.3, could imply longer dry spells between large events. A tendency of the jet stream toward stronger or more prolonged ridges of high pressure—with their associated high temperatures, sunny skies, and lack of rainfall—would increase the risk of rapid‐onset “flash droughts.”
- Higher temperatures in the warmer months could increase potential evapotranspiration and outweigh the influence of additional precipitation.^85^ Furthermore, as described in Section 4.2, climate models disagree on whether precipitation will increase or decrease in summer and fall.
- Reduced snow cover (Section 4.4) and earlier spring snowmelt could increase drought risk in the summer as soils dry out earlier.^4^, ^86^

The possibility of increased summer droughts described here is consistent with the results of earlier analyses, including Zhao et al.^87^ and the 2014 ClimAID projections,^88^ showing that late‐summer, short‐term droughts will likely become more frequent in New York.

### 5.3 Wildfire

Wildfires are unplanned or unwanted fires that burn vegetation. While wildfires do not typically occur in New York State at the scale seen in other parts of the United States, the state does experience fires every year. These fires burn hundreds to thousands of acres per year of New York's forests, marshlands, and other ecosystem types. Wildfires can directly harm people and structures, and they also produce particulate air pollution that can pose a hazard to human health.^89^

#### 5.3.1 Historical observations

From 1919 to 2018, New York experienced an average of 912 wildfires and 13,164 acres burned per year (Figure 2‐19). Both the number and extent of fires have declined over this 100‐year period. This period has actually seen an increase in “fire weather” conditions (high temperature, low humidity, high wind) across most of the state,^90^ consistent with the observed warm‐season temperature increases described in Section 3.2. Fire prevention and suppression activities have also increased; examples include bans on brush burning, changes in forest management practices, tighter regulations on logging and railroads after a series of large fires in the Adirondacks in the early 1900s, and enhanced networks (e.g., fire towers in the Adirondacks that were staffed for many years) to detect and respond to forest fires.^91^, ^92^, ^93^

**FIGURE 2‐19:**
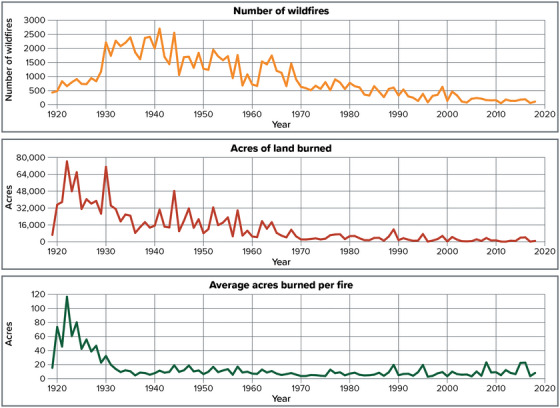
Number and extent of wildfires in New York State, 1919–2018. Data from New York State Department of Environmental Conservation (2023).^94^

The New York State Department of Environmental Protection reports that nearly all (95%) of wildfires in New York from 1993 to 2017 were started by humans, with debris burning as the most common cause.^93^ Nearly 50% of the state's wildfires occur from March 15 to May 15.^93^

New York State can also be affected by wildfires that occur in other states and Canada. For example, during June 2023, smoke from large wildfires in Canada blanketed New York, causing unhealthy air quality in much of the state.^95^, ^96^ New York City reported the worst air quality of any city worldwide on June 7, far surpassing the city's previous record‐high concentration of fine particulate matter.^97^, ^98^ A preliminary study suggests that climate change more than doubled the likelihood of the extremely warm, dry, and windy conditions that fueled these record‐setting fires.^99^ This finding is consistent with widespread agreement in the literature that observed climate change has extended the fire season and increased the risk of wildfires in North America.^100^, ^101^

#### 5.3.2 Projections of future change

Climate change is expected to increase the frequency and intensity of wildfires in many states but is not likely to greatly expand the risk of wildfires in most of New York. Even though studies have projected percentage‐wise increases in annual fire probability for the state, the baseline fire probability is so low that the absolute projected change in fire occurrence is small, even under the highest greenhouse gas emissions scenario.^102^ One projection found that even under the highest emissions scenario, end‐of‐century fire probability in most of New York remained in the lowest category, with an annual probability of occurrence less than 0.1%.^103^ These projections largely agree with a 2022 study^104^ whose baseline (1984–2018) fire probability was modeled at 0% and whose fire probability for 2080–2099 under the intermediate, high, and very high emissions scenarios remained at 0%.

However, additional studies project that the fire season in the Northeast will begin earlier in the year, peak earlier, and last longer.^105^ These changes are associated with a projected increase in weather conditions conducive to wildfire ignition and spread—a combination of temperature, moisture, and wind known as “fire weather.”^105^ Studies also project that the risk of widespread air quality impacts will increase in future decades as a result of the increased risk of large fires elsewhere in North America.^106^

## 6 OCEAN CONDITIONS

### 6.1 Sea surface temperature

Sea surface temperature, the temperature of the water at the ocean surface, is a key indicator of climate change. Because the ocean surface absorbs heat from the atmosphere, sea surface temperature is usually the warmest near the equator and coldest in the Arctic and Antarctic. Changes in sea surface temperature affect ocean circulation patterns, further influencing global climate through the transfer of heat back into the atmosphere.^107^ Sea surface temperature also affects storm formation, other aspects of weather, and marine life.

#### 6.1.1 Historical observations

Global average sea surface temperature increased from 1901 to 2020 at an average rate of 0.14°F per decade, with the largest increases starting around 1970.^107^ Nearly all locations within the world's oceans have experienced statistically significant surface warming, including the entire northeastern U.S. coast.^107^

Observed changes vary regionally. Along New York's coast, water temperatures are influenced by the speed and locations of Atlantic circulation patterns, including a weakening Atlantic Meridional Overturning Circulation, poleward movement of the Gulf Stream, and retreat of cold Labrador waters such that a higher proportion of warm water is reaching the New York coast.^108^, ^109^, ^110^ Observed sea surface temperature trends in the region have included:

- Warming of the northwest Atlantic overall by 0.67°F (0.37°C) per decade from 1982 to 2018.^111^ This rate is three times faster than the global average rate of 0.18°F per decade during a similar time frame.^16^ The rate of warming has accelerated in recent years; warming within the northeastern U.S. continental shelf region quadrupled during the period 2007–2016 compared with long‐term trends.^16^, ^50^
- Warming of about 0.54°F (0.3°C) per decade for waters relatively close to New York—the inner portion of the Mid‐Atlantic Bight—from 1982 to 2014.^112^
- Warming of about 0.54°F (0.3°C) per decade for the Central Basin of Long Island Sound from 1948 to 2012, based on monthly temperature data provided by NOAA's Milford Laboratory in Connecticut.^113^
- Increases in all four seasons within the New York Bight since the 1980s, with the greatest warming in summer and fall.^114^

Marine heat waves (multiday periods with unusually warm surface water temperatures) occurred much more frequently in the New York Bight from 2010 to 2020 than in the preceding three decades (Figure 2‐20). In 2011 and 2015, more than half the days of the year had water temperatures higher than the historical threshold for defining a marine heat wave, defined as 5 days or more exceeding the 90th percentile of historic average surface temperature.^114^

**FIGURE 2‐20:**
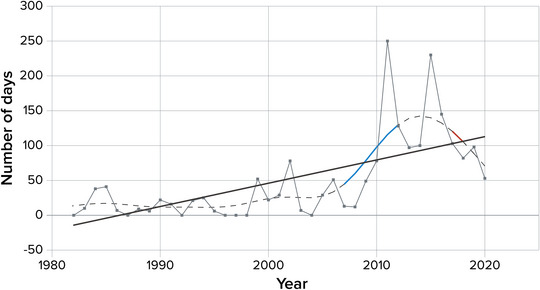
Marine heat wave days in the New York Bight, 1982–2020. Marine heat waves are defined here as events in which the surface temperature exceeds the 90th percentile of the historical average for at least 5 days. The solid gray line with circular markers shows annual data points, the dashed black line shows a nonlinear generalized additive model, and the blue and red segments show statistically significant increases and decreases, respectively, in the nonlinear model. The solid black line shows the ordinary least‐squares linear trend (+3.4 days per year), which is significant to a 99% confidence level (*p* < 0.001). Figure from Gruenburg et al.^114^

#### 6.1.2 Projections of future change

As global and regional air temperatures continue to increase, and as the oceans ultimately absorb much of the extra heat trapped by greenhouse gases, global and regional sea surface temperatures are also expected to continue rising. The Atlantic Meridional Overturning Circulation (a driver of major ocean currents) is projected to continue to weaken, corresponding with expected warming in the Northwest Atlantic,^109^ although scientists continue to debate some of the uncertainties in ocean circulation projections. Climate projections indicate that this part of the ocean will continue to experience more warming than many other parts of the world.^108^ Northeastern U.S. coastal waters have been projected to warm at a rate of up to 0.76°F (0.42°C) per decade through the period 2070–2099 under the SSP5‐8.5 very high emissions scenario.^115^

### 6.2 Sea level and coastal flooding

Global warming also raises global sea level. Two main factors drive this change: (1) water expands as it warms, and (2) a net loss of ice from ice sheets and glaciers adds to the volume of water contained in the ocean. Other contributing factors include ocean currents, circulation patterns, and gravitational effects of ice sheet loss that modify sea level regionally. Additionally, vertical land movement influences the way changing global sea level is perceived relative to the shoreline. Much of the U.S. Mid‐Atlantic coast is at particular risk because of negative vertical land movement (i.e., the land is sinking at the same time the global sea level is rising). This land subsidence is due to a combination of sediment compaction, groundwater extraction, and glacial isostatic adjustment, which is the slow process in which the Earth's crust adjusts to the long‐ago loss of Ice Age ice sheets.^116^ New York State's coastal areas are generally subsiding at a rate of 1–2 millimeters per year.^116^

Sea level rise adds substantial vulnerability to densely populated and ecologically rich coastal zones.^117^ Coastal areas such as Long Island, New York City, southern Westchester County, and the southern Hudson River Valley are at risk of rising sea levels and enhanced coastal flooding. Water levels in the Hudson are influenced by the tides—and, therefore, by sea level—all the way up to the Federal Dam at Troy.

#### 6.2.1 Historical observations

Tide gauges operated by NOAA's National Ocean Service have measured water levels along U.S. coastlines continuously for more than a century. New York has three gauges with long‐term records: one at the eastern tip of Long Island (Montauk) and two in or adjacent to New York City (The Battery at the southern tip of Manhattan and Kings Point on Long Island Sound in Nassau County).

The rate of local or relative sea level rise in New York City averaged 0.11 inches (2.90 millimeters) per year from 1856 to 2022 as measured by The Battery's tide gauge, amounting to almost a foot of sea level rise in the last 100 years (Figure 2‐21). The rate has accelerated to approximately 0.16 inches per year over the past 40 years. At Montauk, the sea level has risen relative to the shore at a rate of 0.14 inches (3.43 millimeters) per year from the start of measurement (1947) to 2022. Both sites’ long‐term rates are double the 1920–2022 global average rate, due largely to the regional subsidence caused by glacial isostatic adjustment as described above.^4^

**FIGURE 2‐21:**
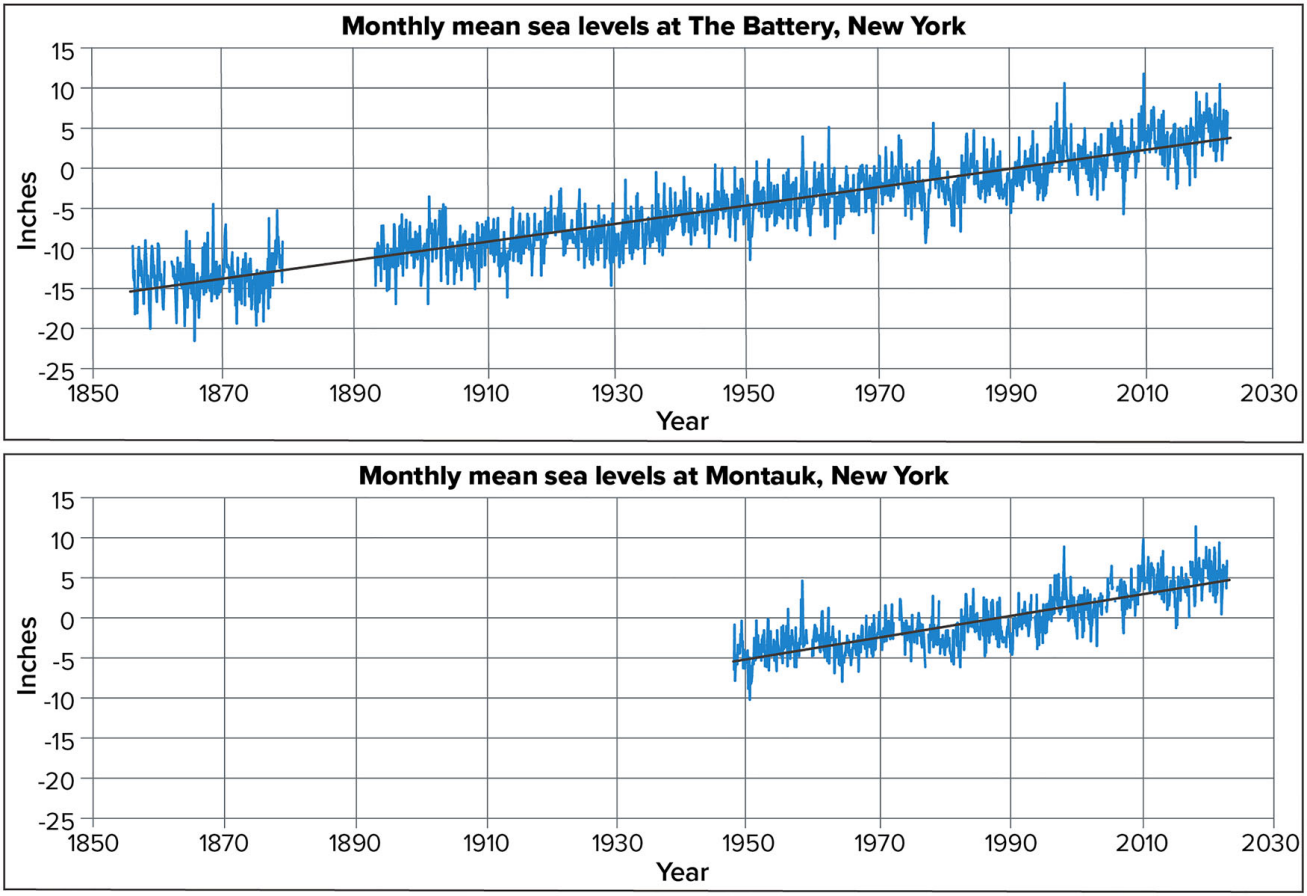
Relative sea level at two long‐term measurement sites in New York State, 1856–2022 (The Battery) and 1947–2022 (Montauk). Each graph shows sea level relative to the shore, which accounts for global sea level rise as well as local vertical land motion. Each graph shows monthly averages, adjusted to exclude regular seasonal fluctuations from coastal ocean temperatures, salinity, wind, atmospheric pressure, and ocean current. “Zero” inches represents the most recent tidal datum (official reference elevation) established for each site, so data are plotted relative to this standard height. Ordinary least‐squares linear trends are shown for reference; changes at both The Battery (+0.11 inches per year over the full period shown, *p* < 0.001) and Montauk (+0.14 inches per year, *p* < 0.001) are statistically significant to a 99% confidence level. Data from NOAA (2023).^7^

Although storm surges have historically caused more damage, high tide flooding (often referred to as “nuisance” or “sunny day” flooding) is also becoming increasingly common along New York State's tidally influenced coastline. As the sea level has risen in New York, so has the number of coastal flood days (Table 2‐4). For example, Table 2‐4 shows that floods that happened an average of twice a year at Kings Point in the 1950s and 1960s now occur an average of 10 times a year. Studies have shown that the observed increase in high tide flooding can be attributed to sea level rise: of 279 flood days recorded at Kings Point and 63 at The Battery from 1985 to 2014, 111 days at Kings Point 34 at The Battery would likely not have happened without sea level rise.^118^ Dredging and filling of wetlands has also increased flood risk in urbanized areas of New York. For example, modeling shows that most of the 15 minor flood events observed in Jamaica Bay in 2020 would not have happened without sea level rise and landscape changes since the 1870s: only one would have occurred without sea level rise, and only two would have occurred without landscape changes.^119^ In some low‐lying coastal neighborhoods such as Howard Beach in Queens, New York City, high tide floods submerge streets even before reaching the established flood threshold.^119^

BOX 3 **Community science to document changing water levels**

The MyCoast New York portal enables New Yorkers to submit and analyze photos of changing water levels, shorelines, and hazardous weather impacts across the state's varied coasts and water bodies. Photos are linked to real‐time environmental conditions to create reports that help stakeholders such as government agencies, business owners, and residents understand the changing environment and make informed decisions. Volunteers have submitted hundreds of reports of both inland and coastal flooding across New York State.^120^

#### 6.2.2 Projections of future change

Sea level is projected to rise along the New York State coastline and in the tidal Hudson throughout the 21st century and beyond. Table 2‐3 presents projections from this assessment, all computed relative to a 1995–2014 baseline. These projections show sea level rising by up to 1 foot by the 2030s, about 2–3 feet by the 2080s, and more than 4 feet by the year 2150. Under certain scenarios with a high rate of ice loss from Greenland, Antarctica, and glaciers—such as a collapse of the West Antarctic ice sheet—New York could plausibly experience sea level rise much higher than 4 feet by 2150. The ranges shown in Table 2‐3 reflect a combination of three possible scenarios, including a rapid ice melt scenario at the upper end. Looking at a rapid ice melt scenario alone (not blended with other scenarios) would lead to higher estimates. For example, the 2019 New York City Panel on Climate Change report examined an Antarctic rapid ice melt (ARIM) scenario and projected that it would lead to a sea level rise of 81 inches by the 2080s and 114 inches by the year 2100 in New York City.^4^ Local and regional factors are likely to continue to cause New York's sea level to rise more than the global average, based on projections through 2150 for the northeastern U.S. coast (Maine to Virginia).^121^

**TABLE 2‐3:** Projected sea level rise at three locations in New York State during the 21st and 22nd centuries.

Station	2030s (inches)	2050s (inches)	2080s (inches)	2100 (inches)	2150 (inches)
Montauk	8–12	15–21	26–41	32–54	50–94
The Battery	7–11	14–19	25–39	30–50	47–89
Albany (Troy Dam)	7–10	12–17	21–35	25–46	41–82

*Note*: Sea level rise in inches relative to a 1995–2014 baseline. Ranges given represent the 25th−75th percentiles of a blended set of three scenarios used by the IPCC: SSP2‐4.5 with medium confidence, SSP5‐8.5 with medium confidence, and SSP5‐8.5 with low confidence. The latter scenario reflects the plausibility of higher‐end sea level rise associated with accelerated loss of land‐based ice. Data from projections developed for this assessment.^3^

Sea level rise is expected to continue to increase the height and frequency of the state's coastal floods in future decades. While New York City currently experiences approximately 10 high tide floods per year as measured at The Battery, that number could rise to 60–85 days by the 2040s (Table 2‐4). This projection means chronic flooding could affect low‐lying coastal neighborhoods once a week or more. Regular tidal flood events will progressively affect additional low‐lying coastal areas, such as the Rockaways and other neighborhoods near Jamaica Bay (Figure 2‐22). Furthermore, the combination of sea level rise and storm surge will increase the frequency, extent, and severity of coastal flooding even if storm patterns remain the same^122^—and, as Section 5.1 explains, coastal storms are also projected to become more intense over time.

**TABLE 2‐4:** Observed and projected high tide flooding at three locations in New York State.

Station	Flood threshold (height above mean higher high water)	Flood days per year, 1950–1969	Flood days per year, 1970–1989	Flood days per year, 1990–2009	Flood days per year, 2010–2022	Projected flood days per year, 2041–2050
Montauk	20.9 inches (0.53 meters)	1.2	0.9	2.3	4.3	50–90
The Battery	22.0 inches (0.56 meters)	1.9	1.8	4.3	10.0	60–85
Kings Point	23.6 inches (0.60 meters)	3.6	3.1	5.0	8.7	50–70

*Note*: High tide flooding represents the number of days that the water level reaches a station‐specific, probabilistically derived threshold as defined by NOAA.^122^ Each number here represents an annual average over the time period shown. Projections represent the “likely decadal range” in NOAA's Annual High Tide Flooding Outlook, which extends from NOAA's “low” sea level rise scenario to NOAA's “intermediate” scenario. Scenarios are defined in Sweet et al.^121^ The SSP2‐4.5 and SSP5‐8.5 emissions scenarios used elsewhere in this assessment fall within the likely range represented by these two NOAA sea level rise scenarios.^121^ Data from NOAA (2023).^123^, ^124^

**FIGURE 2‐22:**
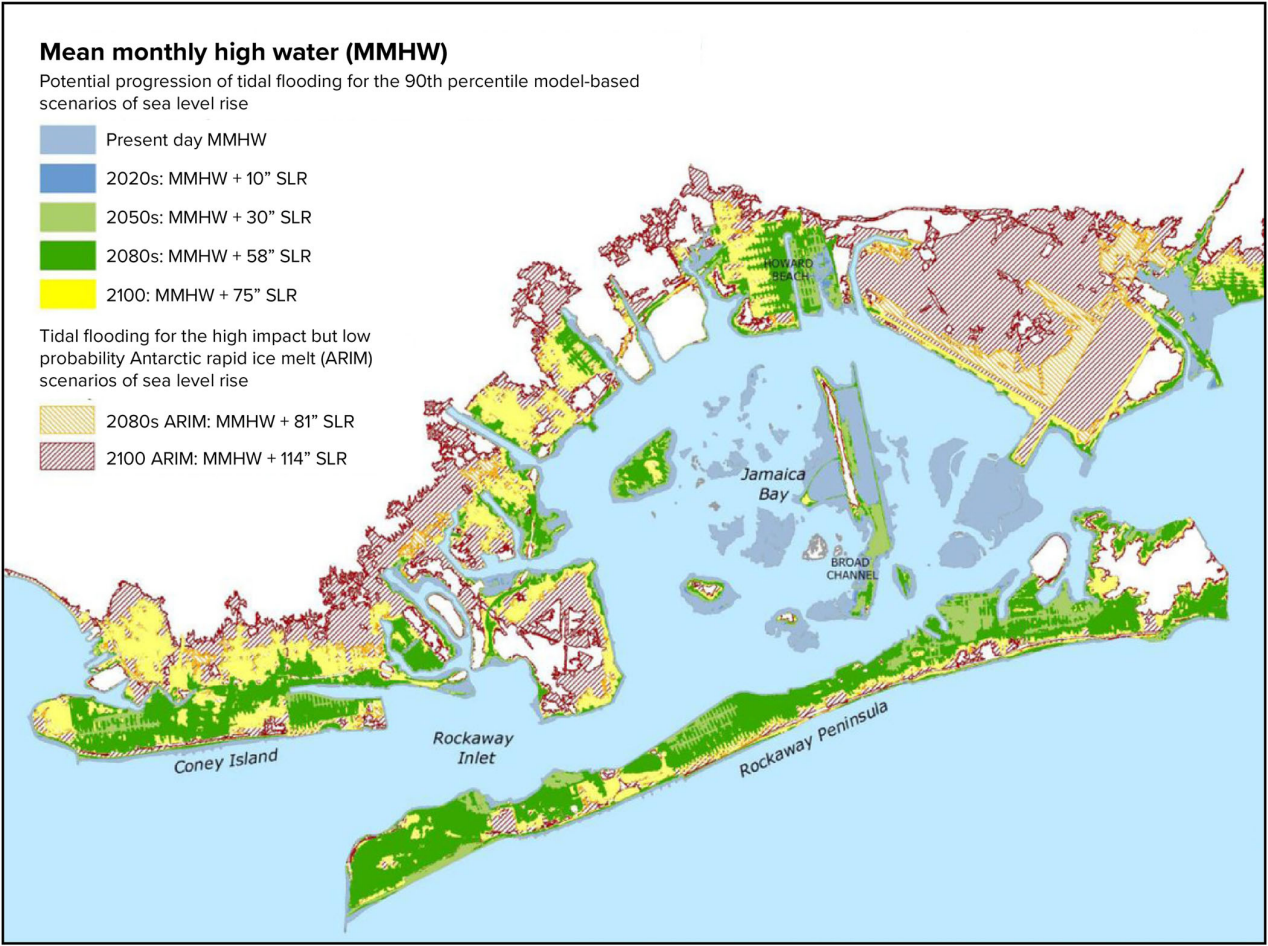
Tidal flooding projections for the Jamaica Bay area of New York City during the 21st century. This map shows mean monthly high water levels (MMHW), from present day through 2100, under 90th percentile model‐based scenarios of sea level rise (SLR) as well as under ARIM scenarios. MMHW is a measurement increasingly used to describe chronic flooding conditions and represents the average of all monthly maxima in predicted astronomical tide levels. Parts of southern Brooklyn and Queens are projected to flood based on these SLR scenarios in the decades ahead. Adapted from a figure by L. Patrick and P. Orton in Orton et al.^60^ Refer to fig. 4‐4 in Orton et al. for disclaimers regarding the interpretation and use of this map.

### 6.3 Acidification

Over the past 250 years, the world's oceans have absorbed about 28% of the carbon dioxide produced by human activities.^125^ Carbon dioxide reacts with sea water to produce carbonic acid, so an increase in dissolved carbon dioxide leads to an increase in acidity (lower pH values).^126^ This in turn changes the balance of minerals in the water and makes it more difficult for shellfish and other organisms to produce calcium carbonate, the main ingredient in their hard skeletons or shells. Thus, declining pH can make it more difficult for these animals to thrive. Such changes can affect ecosystems and the people who depend on them.^102^, ^127^ Local processes that affect acidity in New York's coastal waters also include ocean circulation patterns, natural seasonal variability, upwelling, eutrophication, and stormwater runoff from densely populated coastal urban areas.^128^, ^129^, ^130^ Acidification also occurs in freshwater systems such as the Great Lakes.^131^

#### 6.3.1 Historical observations

It is virtually certain that the ocean's surface globally has undergone increasing acidification by absorbing increasing atmospheric carbon dioxide.^132^ Within the state's coastal waters, there is evidence of increased acidification as shown by measures of surface water dissolved carbon dioxide.^133^ Surveys of surface carbonate chemistry conducted by NOAA and others show large natural seasonal and spatial variability and possible decadal changes.^128^, ^130^ To date, however, trends in acidification within northeastern U.S. coastal waters have related more to regional warming and large‐scale ocean circulation than to changes in atmospheric carbon dioxide.^134^, ^135^ Stormwater runoff is also considered to be a more immediate driver of acidification. Long Island Sound experiences daily and seasonal fluctuations in pH corresponding with low dissolved oxygen and high chlorophyll. The greatest fluctuations occur in the bottom waters of the western Sound. Acidification is also observed in Hempstead Bay and Jamaica Bay, where coastal acidity has reduced the growth and survival of fish and shellfish.^136^

In the Great Lakes, local primary productivity and historical impacts from acid deposition influence seasonal and spatial variability in pH.^137^ Streams and rivers in the eastern United States show increasing trends in alkalinity,^138^ and studies elsewhere support the intuitive conclusion that this trend could work to counteract acidification in coastal waters, at least near the mouths of large rivers.^139^

#### 6.3.2 Projections of future change

Assessments agree that the global ocean will further acidify over the 21st century, exacerbated by continued carbon uptake. Continued acidification over the coming century is projected to result in a further decline of up to 0.3 pH units in the global surface ocean by 2081–2100, relative to 2006–2015, under the very high emissions (SSP5‐8.5) scenario.^132^ The northeastern United States faces elevated risk due to weak buffering capacity, extreme precipitation events, and increased overall precipitation that will add more runoff to coastal waters. As a result, the northeastern U.S. coast is expected to experience some of the earliest impacts from ocean acidification.^129^

The Great Lakes are projected to continue to acidify at a similar rate and magnitude as the ocean as a result of increasing atmospheric carbon dioxide.^131^, ^140^

There are still sizable knowledge gaps related to the drivers of large‐scale variability of regional ocean acidification. These gaps are being addressed by the Mid‐Atlantic Coastal Acidification Network^141^ and the newly created New York Ocean Acidification Task Force initiated by the New York State Department of Environmental Conservation.^130^, ^136^ New studies are underway, including one examining the relationship between acidification and harmful algal blooms.^142^

## 7 LAKES AND RIVERS

### 7.1 Lake and stream water temperature

Climate change—particularly rising temperature and increasing precipitation—affects the thermal and hydrologic dynamics of lakes, rivers, and streams, which in turn affects their biogeochemistry, biodiversity, and ecological processes. Overall, warmer air temperatures increase the temperature of freshwater systems, with variations depending on the depth and other characteristics of lakes. Warming also influences thermal stratification (the separation of water masses by temperature), a key physical attribute of lakes that regulates numerous chemical and biological characteristics. Increased temperature leads to earlier snowmelt, which affects lake and stream water levels along with increased precipitation. New York State has a high density of lakes and streams compared with other parts of the country. Because of the diversity of geographic characteristics across the state, the impacts to freshwater systems will not be uniform statewide.

#### 7.1.1 Historical observations

Evidence indicates that fresh water bodies in New York have warmed over recent decades:

- Surface waters in Lakes Erie and Ontario warmed from 1995 to 2022; both lakes’ changes are significant to a 95% confidence level (Figure 2‐23). The observed warming has been most notable during the spring and summer months.^143^ Additional studies have noted that the northern and easternmost portions of each Great Lake, especially in near‐shore areas, have experienced the highest rates of warming.^144^ Warming surface temperature in the Great Lakes increases rates of evaporation and increases the length of the season for evaporation by delaying lake ice formation, which can contribute to lower water levels (although additional precipitation with climate change can offset this effect).^145^ Refer to Section 7.3 for more discussion on ice cover and Section 7.4 for water levels.
- One study of 231 lakes across northeastern North America, including 15 in New York State, found that overall the lakes had increasing near‐surface temperature and thermal stratification strength from 1975 to 2014.^146^
- Warming surface waters and stable deep‐water temperatures have led to increases in the temperature difference through the water column of many lakes in New York State and throughout the temperate zone worldwide.^147^ These increases in the temperature difference increase the density difference between water column layers and hence increase the strength of stratification. Additionally, the seasonal duration of stratification is increasing by 3.7 days per decade, as lakes worldwide are stratifying earlier than in past decades and seasonal summer stratification is lasting longer into the fall.^148^
- Stream water temperature shows warming trends in the Mid‐Atlantic region, including statistically significant warming from 1960 to 2010 at several stream gauging sites in Pennsylvania, near the New York border.^149^ Many gauging sites within New York also have several decades of temperature measurements available from the U.S. Geological Survey. However, a detailed analysis of warming trends would need to ensure methodological comparability over time and presumably avoid waterways where the water temperature is influenced substantially by hydromodifications or industrial thermal discharges. In New York State, elevation, riparian forest cover, landscape slope, and growing degree days have been found to be the strongest predictors of water temperature in streams.^150^ The statistical association with growing degree days—a measure based on air temperature—supports the assertion that warmer air drives warming in streams.

**FIGURE 2‐23:**
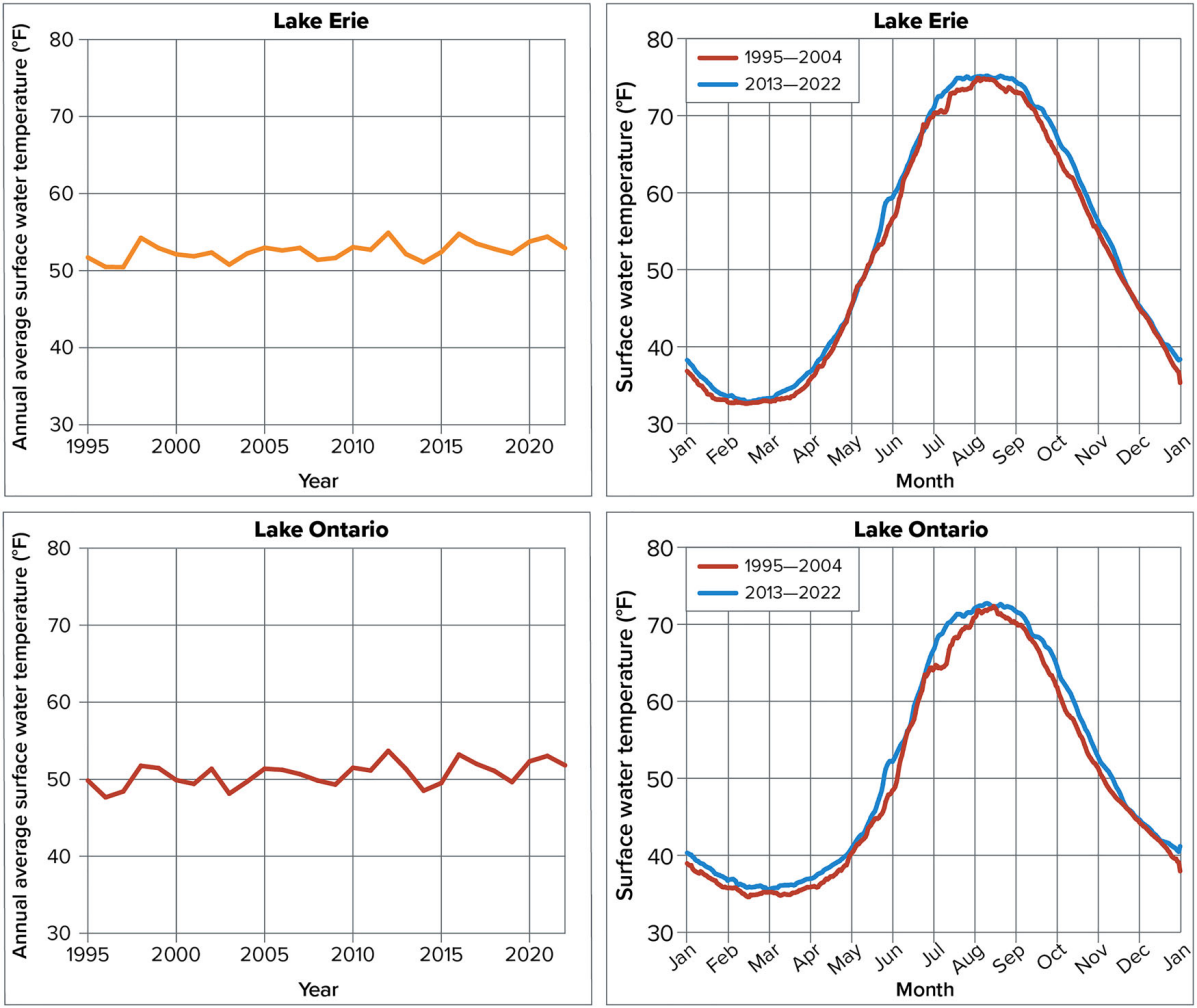
Annual and daily average surface water temperatures of Lake Erie and Lake Ontario, 1995–2022. The graphs on the left show the average annual surface temperature from 1995 to 2022. Ordinary least‐squares linear trends are shown for reference as black lines; Lake Erie (+0.68°F per decade, *p* = 0.012) and Lake Ontario (+0.91°F per decade, *p* = 0.010) changes are statistically significant to a 95% confidence level. The graphs on the right compare daily average temperatures from 1995 to 2004 with daily average temperatures from 2013 to 2022. Data from NOAA Great Lakes Environmental Research Laboratory (2023),^151^ expanding on previous analysis by EPA (2021).^143^

Lakes in the northeastern United States could be changing faster than those in other mid‐latitude regions of the world due to regionally warmer and wetter conditions, but local characteristics contribute to variability in thermal trends.^146^ Nonetheless, the patterns observed in New York are consistent with global trends. In a worldwide study of lakes, surface water temperatures increased at a median rate of 0.61°F (0.34°C) per decade over the period 1985–2009.^152^ Other more recent assessments provide similar trend estimates.^147^, ^153^ Recent global investigations have highlighted an increase in the intensity and duration of aquatic heat waves, which are periods with much greater than normal water temperatures, in some lakes.^154^ However, global evidence indicates that deep‐water temperatures have not consistently warmed, and in some cases have even cooled.^147^, ^153^

Some uncertainties remain. While individual lake temperatures across the state are regularly monitored by government agencies, academic organizations, and individuals via programs such as the Citizens Statewide Lake Assessment Program and the Adirondacks Lakes Survey, there is a lack of comprehensive analysis of these data. As noted above, time‐series analysis of temperature data for streams is also limited.

#### 7.1.2 Projections of future change

Lake warming trends are expected to continue worldwide through the 21st century. Additionally, lakes are projected to experience increasingly severe heat waves in future decades.^154^ For example, under the very high emissions scenario (SSP5‐8.5), lake heat waves are projected to increase in duration worldwide from an average of 8 days per year (over the period 1970–1999) to about 96 days by the end of the 21st century.^154^ Under the same scenario, the annual duration of stratification in Northern Hemisphere lakes is projected to increase by 33 days by 2099.^155^ Within New York, one study of a large, relatively shallow lake (Oneida Lake—the largest lake entirely within New York) projected that by 2100 under a relatively high emissions scenario, water temperatures could increase by 6.7°F (3.70°C) at 2 meters depth and 6.1°F (3.37°C) at 10 meters depth, and the number of consecutive days of stratification could increase by 61 days.^156^

Surface temperatures are projected to increase across all five Great Lakes, but with strong seasonal and spatial variability. The largest increases are projected for Lakes Superior and Ontario.^157^ The strongest warming is projected in spring and extends into summer, due to earlier and more intense stratification.^157^

For rivers and streams, modeling studies show that air temperature strongly predicts water temperature, which is consistent with observed relationships.^149^, ^150^ This finding would suggest continued warming of New York's rivers and streams as air temperatures rise in the decades ahead. Increased streamflow associated with increased precipitation could offset some of this warming effect, however.^149^

### 7.2 Inland flooding

Inland flooding occurs in two forms:

- **Fluvial**: Flooding due to a river, stream, or lake overflowing its banks onto adjacent land. Sustained heavy precipitation and rapid snowmelt are among the conditions that can lead to fluvial flooding.
- **Pluvial**: Flooding as a direct result of intense rainfall that floods an area more quickly than the water can be absorbed or conveyed away by the landscape or by built infrastructure. This category can include flash floods, cases where heavy rain overwhelms an urban stormwater system, and cases where rain falls on a landscape where the ground is already saturated.

Both types of inland flooding are caused by heavy precipitation, so an increase in heavy storms in a changing climate (Section 4.3) will raise the risk of floods.

#### 7.2.1 Historical observations

The U.S. Geological Survey maintains thousands of stream gauges that measure stream water level and discharge across the United States, including many in New York State. From 1965 to 2015, floods measured at stream gauges generally became larger and more frequent in rivers and streams across large parts of the Northeast, including at all 10 stream gauges with adequate long‐term data in New York.^158^ However, only one of the New York sites had a statistically significant increase (Poisson regression with Mann−Kendall test, significant to a 95% confidence level), and it was only for flood magnitude, not frequency.^158^

Recent flood events have severely affected the state, including a 2003 flood in Binghamton,^159^ Hurricane Irene and Tropical Storm Lee in 2011, a storm in August 2014 that broke precipitation records on Long Island,^39^ the remnants of Hurricane Ida in the New York City area in September 2021, and July 2023 flash flooding in the Hudson Valley and the Finger Lakes region.^160^ These events were associated with extreme rates of precipitation.

Floods can be difficult to quantify and track thoroughly over time, as many flood events are not recorded—especially rapidly occurring pluvial floods, which are not measured by stream gauges on rivers. In the absence of systematic data collection on floods themselves, comprehensive data on the monetary damages caused by floods can offer a useful proxy for tracking. Financial damages data show that flooding occurs in every county in New York State, but the risk varies widely, with the largest historical damages occurring in the Susquehanna and Delaware watersheds (Figure 2‐24).

**FIGURE 2‐24:**
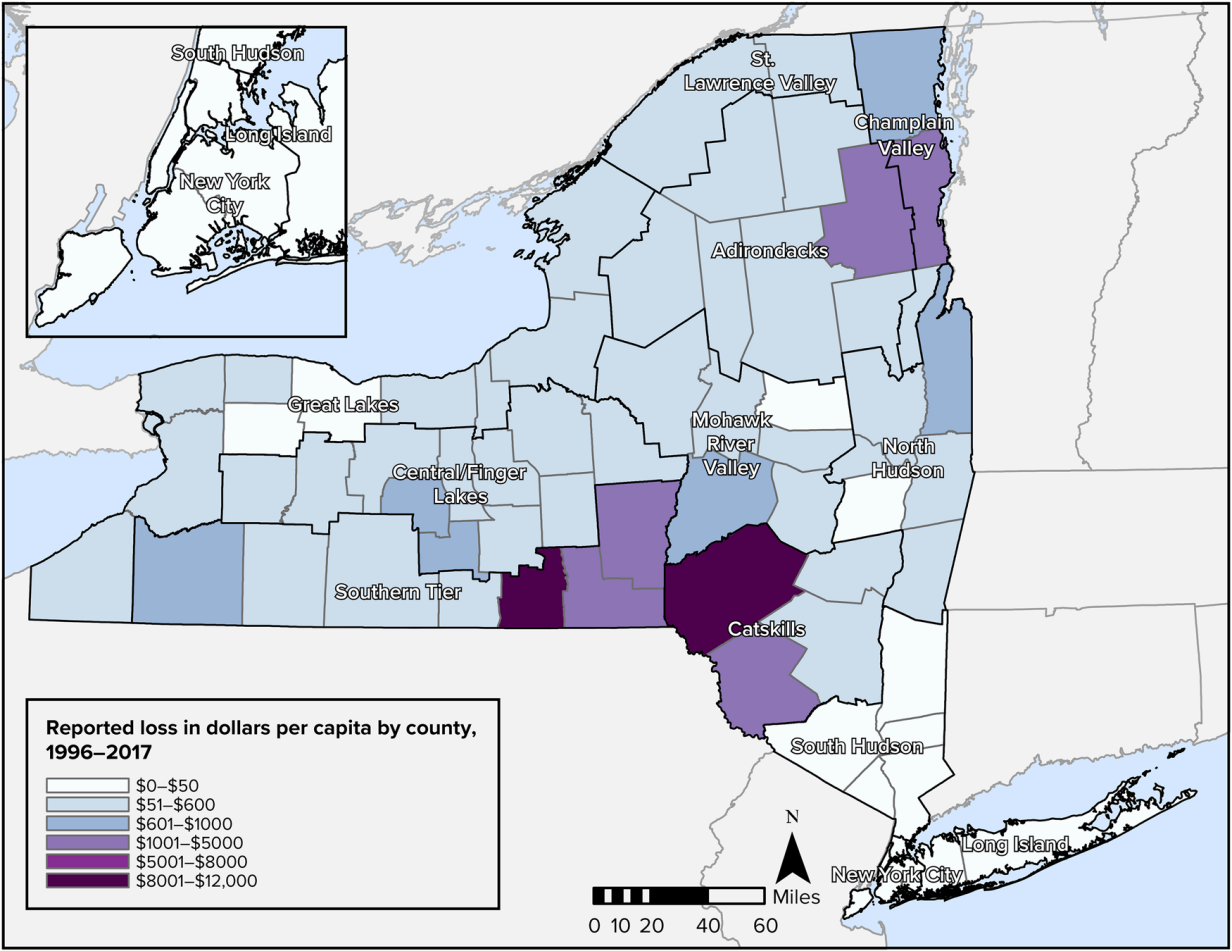
Financial damages from flooding in New York State per capita by county, 1996–2017. This data set does not include damages associated with hurricanes or coastal flooding, nor does it capture inland flooding events since 2017, some of which have been notable. Flood damages from the New York State Division of Homeland Security and Emergency Services (2019),^161^ normalized using population estimates from the New York State Department of Labor (2023)^162^ that have been averaged from 1996 to 2017 by county.

#### 7.2.2 Projections of future change

This assessment did not attempt to quantify future flood risks for specific water bodies in New York, but other studies have reached conclusions relevant to the state:

- Across most regions of the United States, including the Northeast, projected damages from river flooding could more than double with warming around 5.4°F (3°C), which is expected to be reached around the 2050s (Section 3.1).^163^
- EPA's Climate Change Impacts and Risk Analysis program used the First Street Foundation's widely cited flooding risk data and model to generate projections of inland flood risk and damages by census block group and tract across the contiguous 48 states.^164^ This analysis projected that warming of 3.6°F (2.0°C) above the 2001–2020 average will be associated with increased inland flood damages in much of Eastern New York but decreased damages in much of the western part of the state. With 7.2°F (4.0°C) warming, most (though not all) of the state is projected to experience increased inland flood damages.
- Extreme river flows are projected to continue increasing in the northeastern United States in the winter months, associated with wetter weather in the winter. Extreme inland flooding is projected to increase in intensity and extent.^165^
- In the Great Lakes, compound impacts on water level caused by lake seiche (Section 7.5) and high river flow could result in a higher probability of flooding along the shore.^166^

The Water Resources chapter provides more detail on projections of streamflow in general, noting a likely increase in annual discharge along with a shift toward earlier winter/spring peak flows.

### 7.3 Lake ice cover

Ice cover records represent some of the longest‐running observations of the effects of climate change in New York. Climate influences the timing and extent of ice cover on lakes through air temperature, cloud cover, wind, and other conditions within the watershed such as rain and snowmelt. Shorter periods of ice cover on lakes are a sign that the climate is warming. Ice cover duration and extent can affect recreational activities, commercial shipping, and the rate of evaporation—which in turn feeds lake‐effect precipitation downwind of large lakes such as Erie and Ontario.

#### 7.3.1 Historical observations

Many lakes with long‐term records have displayed a clear and substantial decline in ice cover.^167^, ^168^, ^169^ In New York, several lakes have ice cover records documenting the onset, duration, and breakup of winter ice cover for more than a century. The following trends are all significant to a 95% confidence level:

- Mirror Lake (Lake Placid): Ice‐on (freeze) date has shifted later by 11 days since 1903; thaw date has shifted 6 days earlier since 1905.^170^
- Lower Saint Regis Lake (Adirondacks): Thaw date has shifted earlier by 7 days since 1909.^171^
- Otsego Lake (Cooperstown): Freeze date has shifted later by 11 days since 1849.^170^
- Lake George: Thaw date has shifted earlier by 7 days since 1905.^170^
- Lake Champlain has an ice cover record going back to the early 1800s and has exhibited an increase in the frequency of ice‐free winters in recent decades.^172^

These trends are based on a variety of published analyses. Other analyses for the same lakes may calculate a different number of days for each shift, depending on the timeframe, the source data, and the regression and gap‐handling methods used. However, there is widespread agreement on the direction of change.

Increases in winter temperature have also affected ice cover on the Great Lakes.^39^ The duration of ice cover on Lake Ontario has decreased at a rate of 0.87 days per year since the start of systematic measurement in 1973 (linear regression, *p* = 0.005).^173^ Trends vary spatially, with the most significant decrease in the duration of ice cover happening near the shoreline of each lake (Figure 2‐25).^173^ Various studies have also reported decreases in maximum seasonal ice coverage in the Great Lakes since 1973,^50^, ^174^ although a recent analysis of these data found that changes in maximum ice‐covered area were not statistically significant to a 95% confidence level for Lake Erie and Lake Ontario from 1973 to 2020.^173^ Nonetheless, there is some evidence that seasons with lower‐than‐usual ice cover are becoming more common. For example, while Lake Erie has a history of freezing almost completely, there have been 6 years on record when Lake Erie was mostly ice‐free, and all of those occurred since 1998.^39^

**FIGURE 2‐25:**
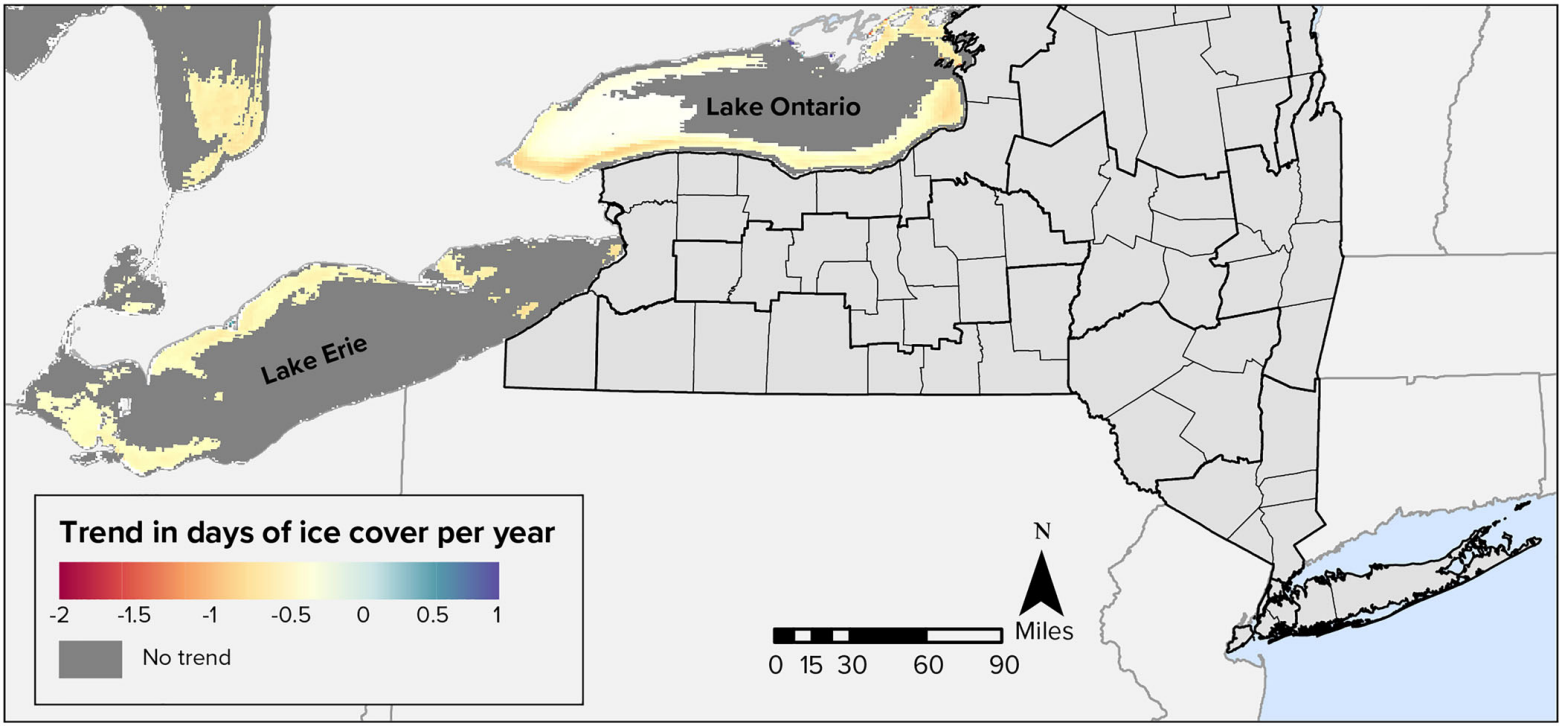
Average annual rate of change in ice cover duration in Lake Erie and Lake Ontario, 1973–2019. The map shows the average rate of change in ice cover duration from 1973 to 2019 for each grid cell where the ordinary least‐squares linear regression trend is significant to a 90% level. All other pixels are gray. A pixel is considered “ice covered” each day that it is at least 10% covered by ice. Data from NOAA Great Lakes Environmental Research Laboratory, analyzed by EPA.^173^

#### 7.3.2 Projections of future change

Ice losses are projected to continue in future decades as winters warm. By 2100, the Great Lakes are projected to have ice covering only 3%−15% of the lake surface during the peak winter month under the very high emissions scenario, and 10%−40% under the intermediate emissions scenario.^157^ Ice duration is projected to decrease by up to 60 days in waters near the shore of the Great Lakes.^157^ Among smaller lakes in New York, Lower Saint Regis Lake in the Adirondacks is expected to lose an additional 7–21 days of ice cover by 2100.^171^

As noted in Section 4.4, reduced ice cover on large lakes, especially the Great Lakes, is expected to contribute to more lake‐effect precipitation over land. This is because ice cover greatly reduces heat flux over lakes, and it has also been shown to cause other changes, such as lower wind speeds, that reduce evaporation.^175^ Removing the constraint of ice cover leads to more evaporation and subsequent precipitation. Studies indicate the possibility of increased lake‐effect snowfall over the next several decades, but more rain instead of snow by later in the century as temperatures continue to rise.^41^, ^48^, ^49^

### 7.4 Lake levels

New York has approximately 580 miles of continuous shoreline along Lake Erie, the Niagara River, Lake Ontario, and the St. Lawrence River. This shoreline supports four major ports that handle 1.1 million tons of inbound and outbound cargo and generate $78 million in business revenue annually.^176^ However, commerce facilitated by the Great Lakes–St. Lawrence Seaway in New York is vulnerable to fluctuations in lake levels. For example, every inch of decrease in lake levels can reduce freighter cargo capacity by 100 tons or more.^177^ Fluctuations in lake levels also can have substantial consequences for ecological health, recreation, tourism, and housing. For example, high lake levels can lead to erosion, flooding, and property damage, as other chapters of this assessment discuss in more detail.

Several factors influence Great Lakes water levels on various timescales. Over hourly timescales, wind can cause surges that raise water levels on one side of the lake, followed by oscillations known as seiches (Section 7.5).^178^ Lake levels are also influenced by seasonal variation. Water levels tend to be higher in the spring and early summer due to snowmelt, rainstorms, and cooler water temperatures leading to less evaporation, whereas water levels tend to be lower in the fall and early winter.^178^ Fluctuations in lake levels over multiyear time scales are caused by a confluence of factors, including long‐term patterns in air and water temperatures, precipitation, runoff, evapotranspiration, and ice duration throughout the Great Lakes basin and beyond.^179^ Water levels in the St. Lawrence River–Lake Ontario system are additionally influenced by the International Joint Commission's regulation of Lake Ontario's outflow at the Moses‐Saunders Power Dam,^180^ although this influence is limited.^178^, ^179^

#### 7.4.1 Historical observations

Water levels in Lake Erie and Lake Ontario have fluctuated since 1860, with 4–5 feet of difference between the lowest and highest recorded annual averages (Figure 2‐26). Following highs recorded during the 1980s and into the 1990s, lake levels subsequently declined, with 1998–2013 characterized by below‐average lake levels. From 2015 to 2020, the Great Lakes experienced some higher‐than‐average, sometimes record‐breaking lake levels attributed to above‐average precipitation and high ice cover.^181^ For example, in 2017 and 2019, Lake Ontario experienced record flooding, prompting New York's governor to declare a state of emergency in counties along the lake in both instances.^182^, ^183^, ^184^ One survey found that this flooding resulted in an average loss of $95,000 per home among affected households.^185^

**FIGURE 2‐26:**
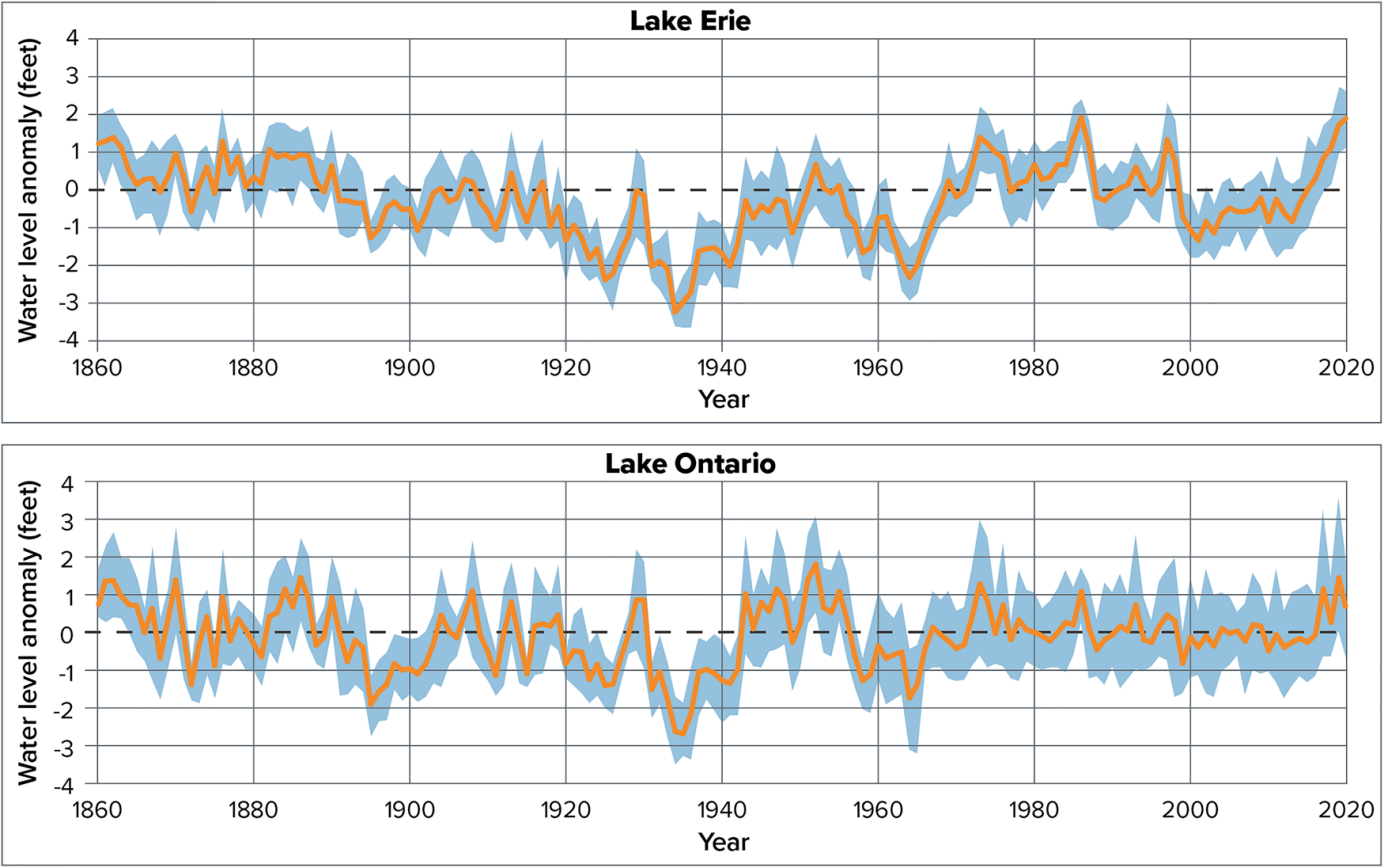
Annual average water level in New York's Great Lakes, 1860–2020. For each year, the shaded band shows the range of monthly average water levels, and the line in the middle shows the annual average. The graph uses the 1981–2010 average as a baseline for depicting change. Data from NOAA Great Lakes Environmental Research Laboratory, analyzed by EPA.^143^

#### 7.4.2 Projections of future change

Projections of future lake levels are limited due to the difficulty of simulating land–air–lake processes and the high spatial resolution needed to generate accurate estimates.^179^, ^181^ However, there is general agreement across models that annual and multiyear variability in Great Lakes water levels will increase. Models suggest there will be “prolonged periods of both high and low levels,” meaning that wide shifts similar to those recently observed in Lake Ontario, from lower‐than‐average levels in 2012 to record highs in 2017, will become more common.^181^ Lake level variability will most likely be driven by periods of drought and extreme precipitation events.^179^ At a seasonal scale, high water levels from late winter through early summer could be enhanced due to anticipated increases in springtime precipitation and runoff, particularly for Lake Ontario.^186^

Although models suggest an increase in variability, there is no scientific consensus as to whether lake levels will increase or decrease overall.^181^ Some older modeling studies predicted a net decline in Great Lakes water levels, while updated models have predicted levels to remain steady or to increase slightly.^145^, ^181^, ^187^ For example, in a 2005 study, all five Great Lakes were projected to experience a water level decrease on average by 2050, including declines of 32.7 inches (0.83 meters) in Lake Erie and 20.9 inches (0.53 meters) in Lake Ontario.^188^ In contrast, more recent projections published by the Great Lakes Integrated Sciences and Assessments suggest that the 2036–2065 average lake levels will be higher than the 1961–2000 average lake levels for Lake Ontario and Lake Erie.^186^, ^189^ Another study in 2022 projected increased water levels, with Lake Erie increasing by about 11 inches (0.28 meters) by 2040–2049 relative to a 2010–2019 baseline.^190^ (This study and some others did not model Lake Ontario because of the different considerations required to account for that lake's regulated outflows.) Using a different method, a 2022 study projected increases of 2.4–3.5 inches (6–9 centimeters) in average Lake Erie water levels through 2050, along with increased month‐to‐month variability and seasonal shifts.^187^ Due to model limitations and the nature of lake level variability, projected trends and extremes in lake levels should be interpreted with caution.

### 7.5 Seiches

Seiches are standing waves that form when strong winds or changes in atmospheric pressure push water toward one end of a body of water. Upon release, water rebounds and begins to oscillate, with oscillations typically having a period of 3 or more hours.^191^ Substantial seiches occur within the Great Lakes, although the magnitude and frequency of these events do not follow a repetitive, spatially uniform pattern like ocean tides. Seiches typically cause the largest water level increases in bays and at the ends of lakes. One recent modeling study quantified the potential contribution of seiches to erosion and flooding at the eastern end of Lake Erie.^192^ Still, the ecological role that seiches play within Great Lakes bays and wetlands remains relatively understudied.^193^

#### 7.5.1 Historical observations

Only limited historical data exist about seiches, as most records of seiches are biased toward extreme events and do not capture smaller, more common day‐to‐day water level fluctuations driven by seiches. One 2006 study found that seiches cause daily water levels to fluctuate an average of approximately 1.6 inches on Lake Ontario and more than 7.9 inches on Lake Erie.^193^ Seiches tend to be more powerful in Lake Erie than in the other Great Lakes because of Lake Erie's orientation, size, shallow depth, and shape. On Lake Erie in particular, extreme seiches can hinder commercial shipping, damage infrastructure, and result in temporary disruptions of drinking water supplies.^194^ For example, in 2008, strong winds caused waves as high as 12–16 feet and led to flooding in some areas of Buffalo.^191^

#### 7.5.2 Projections of future change

Seiches are difficult to predict.^145^ Because of their transient nature and the limited amount of historical data available, seiches are often not considered during discussions of climate change's impact on Great Lakes water levels.^195^ Some researchers have suggested that increased storm frequency and length will produce more and stronger seiches on the Great Lakes,^196^ and others have noted that ice cover suppresses seiche motion, so reduced ice cover could lead to larger seiches and more associated damage during winter.^192^ There is an ongoing need to learn more about seiches and how they could change in a warmer future.^197^

## 8 COMPOUND EVENTS

The extreme events and elevated risks described in Sections 3, 4, 5, 6, 7 can become more severe and damaging when multiple stressors combine or co‐occur. These combinations, referred to as compound events, have occurred historically in New York State. As many of the individual risks increase with climate change, it follows that the risk of increasingly severe combinations will also grow.

Compound events fall into four main categories, all relevant to the state:

- **Preconditioned events** occur when one or more hazards cause an impact, or lead to an amplified impact, only because of a pre‐existing, climate‐driven condition. Examples include heavy rainfall on top of snow and false spring (when cold conditions return after unseasonable warmth).^37^
- **Multivariable events** refer to the co‐occurrence of multiple climate hazards in the same geographic area. Examples include the combined effects of heat and humidity (refer to Section 3.4 regarding the heat index), compound flooding events in coastal storms (e.g., heavy rain, strong winds, storm surge), and the vulnerability of communities along the tidal Hudson River to flooding from a combination of high streamflows and tidal flooding exacerbated by sea level rise.^198^ As an example of the combined effects of heat and humidity, a 2017 study found that under the very high emissions scenario, by about 2070, the northeastern United States could experience approximately 30 days per year where the heat and humidity combined are higher than the level that historically occurred only once per year (i.e., wet‐bulb temperature relative to the 1985–2005 average annual maximum).^199^
- **Temporally compounding events** refer to a succession of hazards that affect a given geographic area, creating or amplifying an impact when compared with a single hazard. Examples include a heat wave after a coastal storm has knocked out power,^200^ a cold snap after a destructive storm (as occurred with Superstorm Sandy in 2012), and back‐to‐back nor'easters associated with persistent troughs in the jet stream. It remains unclear whether events associated with a “stuck” jet stream are becoming more frequent and intense with climate change, given large natural variability and inconclusive results from modeling experiments.
- **Spatially compounding events** occur when multiple connected locations are affected by the same or different hazards within a limited time window, causing an impact. For example, the entire Northeast could experience heavy precipitation over the same period, leading to large increases in riverine flood risk in New York State and beyond. More widespread heat waves associated with climate change could limit energy supplies for New York as neighboring states and provinces face their own high demand for cooling, as broader analyses have suggested.^201^ Other examples could include disrupted supply chains or simultaneous crop failures that affect food prices and food security.^202^

Some authors have considered cases in which compounding goes beyond climate hazards to include other aspects of human and natural systems,^203^ such as the risk of power failures and poor air quality, both of which are correlated with extreme heat. Other chapters of this assessment explore these types of compounding impacts.

## 9 TRACEABLE ACCOUNTS

Traceable accounts examine each key finding in depth. They provide citations that support each assertion and present the authors’ assessment of confidence in each finding.

### 9.1 Key Finding 1

**Average and maximum temperatures have increased in New York State since the early 20th century and are projected to continue to rise throughout the 21st century**. The state has warmed more rapidly than the national average, and winter is warming more rapidly than other seasons. Heat waves are expected to occur more often and become more intense, posing greater risks for human health, built infrastructure, ecosystems, and other sectors. New York City is projected to remain the warmest part of the state; northern regions will continue to be relatively cooler while still experiencing large increases in temperature and extreme heat.

#### 9.1.1 Description of evidence base

Warming of the climate system is widely established as a fundamental component of climate change—a fact supported by a vast body of scientific literature and acknowledged with the highest possible confidence by the USGCRP and IPCC's authoritative national and global assessments.^1^, ^2^ Evidence of observed warming in New York State comes from long‐term, quality‐controlled weather stations that have been carefully screened and selected for use in climatology. Within this data set, stations analyzed for this assessment show warming from 1901 to 2020 in every region of the state, and 24 out of 27 stations had a significant (*p* < 0.01) linear trend in annual mean temperature over this time period.^4^ When averaged statewide, the mean temperature increased at a rate of 0.21°F per decade from 1901 to 2022—a trend that is highly significant (*p* < 0.001) and larger than the contiguous 48 states’ average rate of 0.17°F per decade.^14^ Analysis of seasonal temperature observations from the same long‐term, quality‐controlled data set shows highly significant warming for New York State on the whole in all four seasons over the period 1901–2022 (linear trends with *p* < 0.01 for spring and < 0.001 for the other three seasons).^14^ Among these highly significant trends, winter's regression slope was about twice that of any other season.^14^

The projections developed for this assessment show strong agreement that mean temperatures, extreme temperatures, high heat‐index days (a combination of heat and humidity), and the frequency of multiday heat waves will increase in all regions of New York State during the 21st century. These projections use what are widely acknowledged to be the best climate models currently available (the CMIP6 suite), which reflect the state of the science in global climate modeling and have been used in the most recent IPCC global assessment^1^ and the Fifth National Climate Assessment.^2^ The direction of change is consistent across every available CMIP6 climate model under both emissions scenarios that bound the likely range used for this assessment (SSP2‐4.5 and SSP5‐8.5).

The assertion that more extreme heat raises risks for human health, built infrastructure, ecosystems, and other sectors is backed up by empirical studies and modeled relationships presented throughout this assessment's other chapters.

New York City's projected 50th percentile annual mean temperature for the 2080s, 62.6°F, is higher than that of any other region in the state, and the lower end of New York City's likely range for the 2080s (60.8°F) is equal to the upper bound of the next‐warmest region's likely range (South Hudson). These findings suggest a strong likelihood that New York City will remain the hottest part of the state. The projections show wide agreement that New York's northernmost regions will remain the coolest on average, but all regions are projected to warm by a similar amount (5–11°F) from the 1981–2010 baseline to the 2080s. The projections cited here for New York State align with projections of increased mean and extreme temperatures reported in national and global assessments.^1^, ^2^

#### 9.1.2 New information and remaining uncertainties

The width of the projected ranges for future mean temperatures given in Section 3.1.2 results in part from the fundamental uncertainty about which greenhouse gas emissions trajectory the world will follow most closely. This uncertainty makes it difficult to predict the magnitude of future change with high precision, even for a variable as well studied and closely related to greenhouse gas concentrations as temperature. However, the overall direction of change in New York State (i.e., increasing mean and extreme temperatures) is projected to be the same regardless of where actual greenhouse gas emissions fall within the likely range.

Although observed trends show greater warming in winter than in other seasons, there is too much uncertainty in the climate models to conclude that this trend will continue.

Some studies suggest that extremely high daily temperatures and multiday heat wave frequency, duration, and intensity have already increased in New York State. Multiple studies using data from long‐term, quality‐controlled weather stations have found increases in extreme heat in New York State using a variety of definitions and thresholds (e.g., heat waves using absolute vs. local percentile‐based thresholds, and based on either maximum or minimum daily temperatures).^12^, ^13^, ^23^ Using certain definitions, however, certain weather stations in New York State have not experienced statistically significant changes in these phenomena. Thus, this key finding does not claim an observed increase in extreme heat. Still, no stations experienced significant decreases in extreme heat in the studies analyzed for this assessment.

#### 9.1.3 Assessment of confidence based on evidence

There is **very high** confidence in all portions of this finding, given the preponderance of observed evidence from widespread long‐term monitoring, agreement among all models and climate scenarios regarding the direction of future change in mean and extreme temperatures, and consistency with highly confident findings from authoritative national and global assessments.

### 9.2 Key Finding 2

**New York State has experienced increases in total precipitation and heavy precipitation events, and these trends will continue through the end of this century**. Heavy rainstorms that lead to flooding are projected to become more frequent across the state. Precipitation is expected to increase the most in winter. Lake‐effect snowfall is projected to increase over the next few decades, but as temperatures continue to rise, more winter precipitation near the Great Lakes will fall as rain. Elsewhere in the state, snowfall and snowpack are likely to decrease with warmer winter temperatures.

#### 9.2.1 Description of evidence base

Evidence of observed increases in total precipitation in New York State comes from long‐term, quality‐controlled weather stations that have been carefully screened and selected for use in climatology. Within this data set, 19 of the 27 stations analyzed for this assessment had statistically significant increases in total annual precipitation from 1901 to 2020 (*p* < 0.05), and none of the others experienced a significant decrease.^4^ The assessment team's separate analysis of data aggregated statewide found a highly significant (*p* < 0.001) increase in annual total precipitation from 1901 to 2022, at an average long‐term linear rate of 0.47 inches per decade.^14^ For comparison, the long‐term statewide average over this time frame was 41.1 inches per year.^14^ Along the linear line of best fit, calculations show an estimated change from 1901 to 2022 that equates to a 13% increase.

Similarly, multiple analyses of long‐term, quality‐controlled weather station data show increases in the observed frequency of 1‐ and 2‐inch precipitation events across New York. For days with at least 1 inch, analysis by the assessment team found significant (*p* < 0.05) increases in frequency over the entire period of record available at seven out of nine individual weather stations that were used for this assessment and had data back to at least the 1950s (and in some cases, much earlier).^12^ Analysis of the same stations found two sites with significant increases in days with at least 2 inches.^12^ Looking at the period since the 1950s and using additional data, the NRCC found increases in the frequency of 2‐inch precipitation events and the occurrence of storms previously considered once in 100‐year events across New York and New England.^40^ Evidence that severe rainstorms can lead to flooding is intuitive but also documented widely in the literature, case studies, and popular media.

The projections developed for this assessment show strong agreement that total precipitation will increase progressively through the 21st century in every region of New York State. By the 2080s, precipitation is projected to be 6%−17% higher than it was during the 1981–2010 baseline. This assessment's projections also show days with 1 and 2 inches of precipitation becoming more frequent at all 19 stations modeled, and they show the most extreme events modeled (4 or more inches in a day) becoming more frequent at 12 of the 19 stations by the 2080s. As noted in the traceable account for Key Finding 1, these projections use the most advanced GCMs currently available (the CMIP6 suite), which reflect the state of the science in global climate modeling and have been used in the most recent IPCC global assessment^1^ and the Fifth National Climate Assessment.^2^ The direction of change is consistent across every available CMIP6 climate model under both emissions scenarios that bound the likely range used for this assessment (SSP2‐4.5 and SSP5‐8.5). The projections presented here are consistent with a long series of projections showing increased total and heavy precipitation in the northeastern United States over the 21st century, including projections in the Fifth National Climate Assessment's “Northeast” chapter.^204^ From a seasonal perspective, the Fourth National Climate Assessment took a closer look and projected that the largest increases in precipitation in the Northeast will occur in winter and spring.^16^ This assessment's newer, New York‐focused modeling yielded similar results, with all models agreeing that winter and spring will experience increasingly more precipitation compared with the 1981–2010 baseline. The models do not agree on the direction of change for summer and fall. This assessment's modeled percentage increases are higher for winter than for spring, though the likely ranges (as defined for this assessment) overlap.

Projections of future snowfall come from multiple modeling studies documented in the scientific literature.^16^, ^48^, ^49^ The Fourth National Climate Assessment cited several studies in concluding that the Northeast overall will receive less early‐season snow, an increasing share of winter precipitation falling as rain, and a shorter snow season.^16^ Earlier modeling by Kunkel et al. showed a mechanism by which lake‐effect snow could be the exception to this trend, with increases projected over the next few decades as a result of warmer water and decreased ice cover on the Great Lakes.^49^ Further research has corroborated this connection, noting several ways in which ice cover reduces evaporation^175^ and projecting ongoing declines in Great Lakes ice cover.^157^ However, Kunkel et al. projected more lake‐effect rain instead of snow later in the 21st century.^49^ This conclusion is supported by newer downscaled modeling tailored to the Great Lakes region, which projected a reduced frequency of heavy lake‐effect snowstorms throughout most of the Great Lakes basin by the late 21st century.^48^ This finding is consistent with the Fourth National Climate Assessment, which concluded about the Great Lakes in general: “As the warming in the Midwest continues, reductions in lake ice may increase the frequency of lake‐effect snows until winters become so warm that snowfall events shift to rain.”^205^ The same recent study that modeled lake‐effect snow for the Great Lakes basin also projected that New York as a whole will receive 20 to more than 50 fewer inches of snowfall per year (a loss reported in the original source as 50 to more than 130 centimeters) in 2080–2099, compared with 1980–1999.^48^

The projection of reduced snowpack comes from the Fourth National Climate Assessment, which assessed the literature and found wide agreement that reduced snowfall and warmer winter temperatures overall in the Northeast during the 21st century will also correspond with reduced depth and water equivalent of snow on the ground.^16^

#### 9.2.2 New information and remaining uncertainties

The width of the ranges given for each of the projections cited here results in part from the fundamental uncertainty about which greenhouse gas emissions trajectory the world will follow most closely. As with temperature, this uncertainty makes it difficult to predict the magnitude of future changes in precipitation with high precision. However, the overall direction of change toward more total precipitation and more frequent heavy storms is projected to be the same regardless of where actual greenhouse gas emissions fall within the likely range.

Although the climate models show confidence in projected increases in winter and spring precipitation, the models have too much disagreement and uncertainty to conclude whether precipitation will increase or decrease during summer and fall. Due to resolution constraints, the models cannot simulate many of the relevant storm types that deliver precipitation to New York, including hurricanes, mesoscale convective systems, or even frontal systems.

Future trends in lake‐effect snowfall are somewhat uncertain because seasonal warming drives two opposing effects: it adds moisture to the atmosphere and increases storm formation (warmer air and water, less ice cover to constrain evaporation) but it can also warm the air above the freezing point and cause more precipitation to fall as rain instead of snow. In simple terms, whether lake‐effect snowfall will increase or decrease in New York will depend on which of these effects dominates. Modeling suggests that lake‐effect snowfall will increase in the nearer term but decrease in the longer term as air temperatures become increasingly too warm for snow, but the magnitude of change and the timing of the switch from increase to decrease remains uncertain.

#### 9.2.3 Assessment of confidence based on evidence

There is **very high** confidence in the observed and projected changes in total annual precipitation and heavy precipitation events, given the preponderance of observed evidence from widespread long‐term monitoring, agreement among all models and climate scenarios regarding the direction of future change in these variables, and consistency with highly confident findings from authoritative national assessments.

There is **high** confidence in the projection that precipitation will increase the most in winter. While the results for any given model, scenario, and assessment region largely show winter precipitation increasing more dramatically than the next closest season—spring—the aggregation across models and scenarios yields wide “likely ranges” that largely overlap between the two seasons.

Overall, the portions of this key finding that relate to snowfall can be given **high** confidence. As reflected in authoritative national assessments and other literature, models offer widespread agreement that winter temperatures in New York will warm, an increasing share of winter precipitation will fall as rain, the snow season will become shorter, and there will be less snow accumulating and persisting on the ground. There is also strong agreement that conditions known to promote lake‐effect precipitation—warmer air and water, reduced ice cover—will become more prevalent over time. However, the way these effects will translate into total snowfall can only be assessed with high (not very high) confidence because of likely regional differences and inherent uncertainties in the relative strength of opposing effects. Specifically, there is a question of when and where the increase in winter precipitation driven by climate change (especially by the warming of large lakes) will actually result in more snowfall, which depends on the extent to which temperatures warm above freezing at a given time and place. That said, multiple studies using different models have concurred that lake‐effect snowfall in New York will likely increase in the near term before declining in the longer term.

### 9.3 Key Finding 3

**Climate change is creating conditions that will increase the frequency and severity of many types of extreme events**. Several types of storms are expected to become more intense, with heavier rainfall, stronger winds, and higher storm surge along the coast driven by sea level rise. Short‐term summer droughts could increase due to changing precipitation patterns and increased temperatures. Wildfires are unlikely to become much more common within New York State due to climate change, but air quality impacts from large fires elsewhere in North America could increase in the future.

#### 9.3.1 Description of evidence base

The first part of this key finding refers to climate conditions that influence extreme events. The conditions in question are largely fundamental climate variables discussed throughout this chapter, with observed changes supported by long‐term, quality‐controlled measurement programs. Projections of ongoing change are supported by downscaled modeling conducted for this assessment using the best climate models currently available (the CMIP6 suite). These models reflect the state of the science in global climate modeling and have been used in the most recent IPCC global assessment^1^ and the Fifth National Climate Assessment.^2^ The following evidence supports the assertion that climate conditions that influence extreme events are changing in New York:

- **Air temperatures** influence storm formation and intensity, drought, and wildfire risk, all through widely established and reasonably intuitive mechanisms. In simple terms, higher air temperatures increase evaporation, feed storms with more moisture and energy, and increase drying on the ground through both evapotranspiration and earlier snowmelt that can leave less moisture on the ground by summer. As discussed in the traceable account for Key Finding 1, analysis of quality‐controlled long‐term weather station records shows a highly significant increase in average annual air temperatures from 1901 to 2022 statewide^14^ and significant increases from 1901 to 2020 at 24 out of 27 individual weather stations examined as part of this assessment. Similarly, the projections developed for this assessment using state‐of‐the‐art GCMs show strong agreement that annual mean temperatures, mean temperatures in all seasons, and extreme temperatures will continue to increase in all regions of New York. These results are consistent with the body of evidence summarized in authoritative global and national climate assessments.^1^, ^2^
- **Water temperatures** in large water bodies like the Atlantic Ocean and the Great Lakes influence storm formation and intensity. Warmer waters feed storms with more moisture (evaporation) and energy.
  As noted below in the traceable account for Key Finding 4 on New York's coastal waters, studies based on satellite and in situ data show consistent agreement that sea surface temperatures have warmed significantly at many spatial scales relevant to New York.^107^, ^111^, ^112^, ^113^, ^114^ Sea surface temperature has increased worldwide and throughout the Atlantic since 1901,^107^ which is relevant to New York because the state experiences tropical cyclones that form over warmer waters far to the south. Modeled projections from the literature agree that sea surface temperature will continue to warm through the 21st century, both worldwide and in waters near New York, under multiple climate scenarios.^108^, ^115^
  Surface waters of Lakes Erie and Ontario warmed significantly (*p* < 0.05) from 1995 to 2022, based on a comprehensive satellite‐based data set developed and maintained by NOAA,^151^ with analysis following an approach published by EPA.^143^ The Fourth and Fifth National Climate Assessments cite multiple additional studies that concur that the Great Lakes have warmed.^205^, ^206^ Projections from a regional climate model optimized for the Great Lakes show surface water temperatures continuing to increase.^157^ This finding is consistent with widespread projections of continued warming of air, water, and land at the Earth's surface.^1^, ^2^
- **Precipitation** influences drought and wildfire risk. Seasonal deficits can be important, especially when combined with rising temperatures that increase evapotranspiration. As documented in the traceable account for Key Finding 2, analysis of records from long‐term, quality‐controlled weather stations shows that total annual precipitation increased to a statistically significant degree across much of New York State from 1901 to 2020. Weather station data show significant increases statewide from 1901 to 2022 in summer and fall precipitation.^14^ Projections developed for this assessment using state‐of‐the‐art CMIP6 GCMs show strong agreement across models and climate scenarios that total annual precipitation will increase progressively through the 21st century in every region of New York State. The direction of change is consistent across every available CMIP6 climate model under both emissions scenarios that bound the likely range used for this assessment (SSP2‐4.5 and SSP5‐8.5). The projections presented here are consistent with a long series of projections showing increased total precipitation in the northeastern United States over the 21st century, including projections in the Fourth National Climate Assessment's “Northeast” chapter.^16^ From a seasonal perspective, the Fourth National Climate Assessment projected that the largest increases in precipitation in the Northeast will occur in winter and spring.^16^ This assessment's newer, New York‐focused modeling yielded similar results, with all models agreeing that winter and spring will bring increasingly more precipitation compared with the 1981–2010 baseline.
- **Sea level** influences storm surge. As sea level rises relative to the shore, it provides a higher base elevation onto which storm surge is added, leading to storm surge that can reach farther inland and cause more destruction. This effect is exacerbated as storms themselves become more intense under the warming conditions described above, with stronger winds driving higher surge. Evidence of observed change comes from tide gauges operated by NOAA, which have measured sea level relative to the shore since 1856 in New York City (The Battery) and since 1947 at Montauk. Both locations have experienced a steady and highly statistically significant (*p* < 0.001) increase in relative sea level over time.^7^ Projections come from modeling conducted for this assessment, which projected further sea level rise along the New York State coastline and in the tidal Hudson throughout the 21st century and beyond under both scenarios analyzed. These locally downscaled projections are consistent with NOAA's official projections updated in 2022^122^ and further supported by the scientific community's recognition that much future sea level has already been “locked in” as a result of continuing deep‐ocean warming and ice‐sheet melt driven by global warming that has already taken place.^1^, ^2^ The observations and projections described here are consistent with widespread reporting of observed and projected global and regional sea level rise in the global and national assessment literature, with the IPCC's Sixth Assessment categorizing observed sea level rise as “high confidence” and continued rise through the 21st century as “virtually certain.”^1^, ^2^
- Projections of **“fire weather”** indices under the very high emissions scenario (SSP5‐8.5), using regional climate models for the northeastern and Great Lakes states, show modest but significant increases through the year 2100 in weather conditions that are conducive to wildfire ignition and spread.^105^

Projected increases in storm intensity are consistent with the expected influence of warmer air and water. They come from several sources:

- Heavy precipitation projections developed for this assessment using the best available suite of GCMs, showing that days with 1 or 2 inches of rainfall are projected to become more frequent at all 19 stations included in this analysis, and that by the 2080s, days with at least 4 inches are also projected to become more frequent at 12 of the 19 stations.
- Agreement in the literature that tropical cyclones in the North Atlantic will become stronger on average over time, with more intense wind and rain.^62^, ^63^, ^64^ The Fourth National Climate Assessment echoed this conclusion.^61^
- Projections from 10 CMIP5 models suggesting that East Coast extratropical cyclones could become more intense and 5%−25% wetter in the future.^66^

The projected increase in storm surge height, and, therefore, in destructive potential, reflects expert judgment based on the fact that its two primary physical drivers—sea level and coastal storm intensity—have both been projected with confidence to increase, as described above. This association is supported by literature.^122^ Attribution studies provide corroborating evidence that rising sea level due to climate change increased the flooding associated with a large coastal storm.^58^

The finding that short‐term summer droughts could increase in frequency or intensity in the future reflects expert judgment in considering the relative contributions of temperature and precipitation—the two fundamental drivers of drought that can be modeled with the most confidence. Projections developed for this assessment indicate that temperatures will increase throughout New York in all seasons (refer to the traceable account for Key Finding 1). Literature has connected this warming to earlier snowmelt in the Northeast, which can lead to more drying of soils by summer.^86^ In contrast, there is disagreement among the CMIP6 GCMs used for this assessment as to whether summer precipitation will increase or decrease in New York during the 21st century. Taken together, this information suggests a reasonable possibility that the factors that increase drought (warmer air leading to more evapotranspiration; earlier snowmelt leading to more drying by summer) will outweigh those that decrease it (additional precipitation).

Projections related to wildfire come from a collection of independent studies published in the scientific literature, three of which focused on New York and the Northeast. Two of these studies examined wildfire risk in New York and reached essentially the same conclusion: that the state's probability of fire occurrence will remain close to zero, even with a percentage‐wise increase over time.^103^, ^104^ A third noted that the length of the fire season in the Northeast will begin earlier in the year, peak earlier, and last longer.^105^ Many other studies have examined wildfires more broadly over North America (e.g., Burke et al.)^106^ and noted that the increased probability of large fires elsewhere could increase the risk of air quality impacts far from the source. The Fifth National Climate Assessment^207^, ^208^ and the USGCRP's 2016 Climate and Health Assessment^209^ both support these conclusions, as they emphasize large projected increases in wildfire occurrence in certain regions (e.g., the West, Alaska) and describe a corresponding deterioration of air quality as a problem that can extend far downwind.

#### 9.3.2 New information and remaining uncertainties

Among factors that contribute to extreme events, wind is an important component of “fire weather.” Projections of future changes in wind speeds remain generally uncertain. Another uncertain factor is lightning, which ignites many wildfires. Projections in the literature disagree as to whether the frequency of lightning strikes will increase or decrease in a warming climate.^70^, ^71^, ^72^, ^73^

Storm formation processes also continue to have some uncertainty. Such processes can be complex, and they remain the subject of ongoing study. Projections of some types of severe storm events, including tornadoes, hail, and derechos, are not readily available.^4^ Ice storm projections are inconclusive.^74^ While some other storm types have reasonably confident projections of increased intensity, there remain uncertainties regarding frequency. Scientific literature suggests that the total number of tropical cyclones in the North Atlantic could stay the same or perhaps decrease slightly^61^, ^62^ and that fewer hurricanes could make landfall along the northeastern U.S. coast.^65^ There is also some question as to whether extratropical cyclones such as nor'easters will become more frequent.^66^

Projections of increased late‐summer, short‐term droughts acknowledge the uncertainty in the underlying precipitation projections. While there is very high confidence that temperatures will increase in all seasons (refer to the traceable account for Key Finding 1), and high confidence in earlier snowmelt (refer to the traceable account for Key Finding 2), other contributing factors are less certain. As the traceable account for Key Finding 2 notes, the GCMs used for this assessment's modeling disagree on whether total summer precipitation in New York State will increase. Even if summer precipitation does increase, it is not certain whether the increase will be sufficient to offset increased evapotranspiration due to warmer air. Direct modeling of future drought index values remains challenging given that results can vary widely depending on one's choice of drought index,^210^ and given the known limitations of relying on temperature‐based proxies for soil moisture.^211^

Wildfires are not well represented by climate models, as their occurrence and magnitude depend on a confluence of factors—some environmental, some human. Within New York State, Figure 2‐19 and associated historical records show readily how much wildfire frequency and extent can be influenced by fire prevention and suppression activities. Modeling and analysis, therefore, tend to focus more on projecting specific conditions (i.e., warm, dry “fire weather”) that have a documented relationship to wildfire activity. Application of coarse spatial models at the local forest scale is uncertain,^102^ suggesting an opportunity for more focused investigations that consider variation in factors that affect wildfire risk, such as soil moisture and wind.

#### 9.3.3 Assessment of confidence based on evidence

This key finding starts with underlying conditions because that is where confidence is arguably the highest. There is **very high** confidence that core climate conditions that influence extreme events have changed in New York and are continuing to change. Observed changes in air temperature, ocean and Great Lakes surface water temperature, total and seasonal precipitation, and sea level are supported by a preponderance of observed evidence from widespread long‐term monitoring. There is agreement among authoritative national and global assessments, other literature, and this assessment's modeling regarding the direction of future change in mean and extreme temperatures; annual, winter, and spring precipitation; and sea level. Other assessments and studies also agree that ocean and Great Lakes surface water temperatures will continue to increase.

Storms are one type of extreme event that can be influenced by these conditions. Storm formation is complex and depends on many factors, so it cannot be projected with as much confidence as a more fundamental variable such as temperature. Nonetheless, there is **high** confidence that several types of storms will become more intense during the 21st century. Although models disagree about projected changes in the frequency of storms, the literature suggests broad agreement among models and researchers that climate conditions will favor the formation of tropical and extratropical cyclones with more intense wind and rain, as well as more heavy rain and snowstorms in general.

Despite the complexity associated with storm intensity, there is **very high** confidence that storm surge will increase in height and, therefore, in destructive potential. The physical mechanisms at play are simple and well‐established. Increased storm intensity (high confidence) would lead to a more destructive storm surge on its own, as would increased sea level (very high confidence). The fact that both factors are projected to increase simultaneously leaves little room to doubt that storm surge will also increase in severity.

There is **medium** confidence that short‐term summer droughts will increase in likelihood or severity. This assessment acknowledges that while there is relatively high confidence in projections of certain drivers of drought, such as warmer summer temperatures and drying associated with earlier snowmelt, climate models disagree on the direction of change in summer precipitation.

There is **medium** confidence that wildfires are unlikely to become much more common within New York State. Two studies cited in this assessment reached this conclusion independently, but wildfires are difficult to model, and more definitive assessments on this matter are limited. Moreover, the events of 2023, including record‐setting fires throughout eastern Canada (Ontario, Quebec, Nova Scotia), suggest at least empirically that the relatively wetter parts of North America are not immune to substantial wildfire activity. Nonetheless, there is **very high** confidence that air quality impacts from larger fires elsewhere in North America could increase in the future, as there is widespread agreement in the literature that fires elsewhere in areas such as the western United States and Canada are likely to increase in extent and severity, along with empirical evidence that larger fires generally lead to higher particulate matter concentrations downwind—including as far away as New York State.

### 9.4 Key Finding 4

**Sea surface temperature, sea level, and coastal flooding are increasing along New York State's coast**. Sea surface temperatures are rising more rapidly in the state than the global average. Sea level along New York's coastline has risen almost 1 foot in the past century and is projected to increase by another 1–2 feet by mid‐century, making chronic flooding more common in low‐lying coastal neighborhoods. Ocean water is also becoming more acidic as it absorbs excess carbon dioxide from the atmosphere, although stormwater runoff currently has a larger effect on acidity in New York's coastal waters.

#### 9.4.1 Description of evidence base

Studies based on satellite and in situ data show consistent agreement that sea surface temperatures have warmed significantly at many spatial scales relevant to New York.^107^, ^111^, ^112^, ^113^, ^114^ Several of these studies focused on the period from 1982 forward, finding linear regression slopes at least three times the global average slope during a similar time frame.^16^, ^212^ Modeled projections from the literature agree that sea surface temperature will continue to warm through the 21st century, both worldwide and in waters near New York, under multiple climate scenarios.^108^, ^115^

Evidence of observed change in sea level comes from tide gauges operated by NOAA, which have measured sea level relative to the shore elevation since 1856 in New York City (The Battery) and since 1947 at Montauk. Both locations have experienced a steady and highly statistically significant (*p* < 0.001) increase in sea level over time.^7^ The assertion that this rise has amounted to nearly a foot in the last century is based on the linear regression line of best fit for each site. The Battery's long‐term rate of change from 1856 to 2022 is an average of 0.11 inches (2.90 millimeters) per year, or 11 inches per 100 years. The actual total change over the most recent 100 years is potentially more than 11 inches, given evidence of nonlinearity (specifically, recent acceleration) noted in New York^7^ and worldwide.^1^ From this perspective, “almost 1 foot” is a conservative statement. Montauk's regression slope of 0.14 inches (3.43 millimeters) per year from 1947 to 2022 indicates an increase of 10.5 inches over 75 years, but one cannot assume that the same rate of change preceded 1947, so the evidence justifies “almost 1 foot” but not more.

Projections of future sea level come from modeling conducted for this assessment, which projected further sea level rise along the New York State coastline and in the tidal Hudson throughout the 21st century and beyond under all scenarios analyzed. The assertion of a further 1‐ to 2‐foot increase by mid‐century is based on ranges of 15–21 inches at Montauk, 14–19 inches at The Battery, and 12–17 inches at Albany in the 2050s, relative to a 1995–2014 baseline. The ranges given here represent the 25th−75th percentiles of a blended set of three scenarios used by the IPCC: SSP2‐4.5 with medium confidence, SSP5‐8.5 with medium confidence, and SSP5‐8.5 with low confidence—the latter reflecting the potential for low‐probability but high‐impact events associated with rapid ice loss. These locally downscaled projections are consistent with NOAA's official projections updated in 2022^121^ and are based on CMIP6 models and the projections developed for the IPCC's Sixth Assessment, along with the latest advances in process understanding, improved and lengthened observational records, and improved ice‐sheet modeling. Projected increases also reflect the scientific community's recognition that much future sea level has already been “locked in” as a result of continuing deep‐ocean warming and ice‐sheet melt driven by global warming that has already taken place.^1^ The observations and projections described here are consistent with widespread reporting of observed and projected global and regional sea level rise in the global and national assessment literature, with the IPCC's Sixth Assessment categorizing observed sea level rise as “high confidence” and continued rise through the 21st century as “virtually certain.”^1^, ^2^

Evidence of increased coastal flooding comes from measurements collected at three long‐term tide gauge stations—The Battery, Kings Point, and Montauk—and analyzed from 1950 forward. This approach represents an objective way to characterize coastal flooding trends, as it relies on precise measurements recorded every 6 min and compared against a fixed “nuisance flooding” threshold that NOAA has established for each location based on its historical record.^122^ The resulting numbers characterize the total number of days per year when each location experienced water levels above the threshold, meaning they can include major storm surge flooding as well as “sunny day” high‐tide flooding exacerbated by sea level rise. At all three locations, such events occurred two to five times more often during the period 2010–2022 as they did during 1950–1969, in terms of average annual frequency.^123^, ^124^ It is also possible to find observations from specific neighborhoods, anecdotal evidence, and descriptions of the areal extent or impact of coastal flooding, but the assertion of a change over time here is based on the systematic long‐term collection of data at tide gauges.

Projections of more frequent coastal flooding, including chronic flooding in low‐lying neighborhoods, are based on a combination of modeling and mapping. Modeling comes from NOAA, whose most recent authoritative projections of future coastal flooding are tied to their 2022 sea level rise projections, modeled for the same tide gauge locations.^124^ Projections of increased flooding along with sea level rise are both intuitive and consistent with national assessments,^213^ particularly considering that New York's coastal lands are known to be subsiding, which exacerbates the problem.^116^ Projections of more frequent chronic flooding in low‐lying neighborhoods in the future reflect the magnitude of NOAA's projections (50–90 days per year across the three New York sites by 2041–2050, reflecting the range from NOAA's low to intermediate scenarios),^124^ digital elevation mapping of low‐lying areas such as has been reported by the New York City Panel on Climate Change,^59^, ^60^ and anecdotal reports that some of New York's coastal neighborhoods experience flooding even before an official flood threshold is reached.^119^

The assertion that ocean water is becoming more acidic as it absorbs excess carbon dioxide from the atmosphere is a global statement based on observed data and scientific studies assessed by authoritative bodies including the IPCC and USGCRP.^1^, ^2^ Evidence that stormwater runoff currently has a larger effect than atmospheric carbon dioxide on acidity in New York's coastal waters comes from a literature review compiled by the New York State Department of Environmental Conservation, which cited a set of association studies.^136^

#### 9.4.2 New information and remaining uncertainties

Projections of future sea level rise continue to improve as scientists advance their understanding of dynamic, nonlinear processes, particularly those that relate to ice sheets and glaciers and the potential for rapid ice loss in the future. The ARIM scenario developed for the 2019 New York City Panel on Climate Change report represents such a high‐impact, low‐probability future event.^117^ Several recent studies (e.g., Slangen et al.^212^) confirm the plausibility of high‐end sea level rise scenarios resulting from rapid ice melt. This assessment attempted to account for this possibility by including an “SSP5‐8.5‐low confidence” scenario when generating the ranges shown in Table 2‐3, but doing so leads to relatively wide ranges, particularly for the later part of the time frame. Moreover, some plausible outcomes lie beyond the upper bounds presented in Table 2‐3.

Systematic observed measurements of acidity are relatively limited in New York's coastal waters, and acidity (e.g., pH) was not among the variables modeled for this assessment. The mechanism by which increased carbon dioxide concentrations in the atmosphere drive acidification is unquestioned basic science, but other factors also contribute to acidity in specific coastal areas. The interplay of these factors and the ability to model their relative contributions could benefit from further study.

#### 9.4.3 Assessment of confidence based on evidence

There is **very high** confidence that sea surface temperature, sea level, and coastal flooding have increased historically and will continue to increase during the 21st century as described in this key finding. This assessment of confidence reflects the strength of evidence, with complete agreement across observational data sets and models regarding the direction of change, wide concurrence in the scientific literature (including previous assessments), and alignment with well‐established scientific mechanisms.

There is **very high** confidence that the world's oceans have and will continue to become more acidic as carbon dioxide concentrations increase in the atmosphere. This is another fundamental scientific relationship, with supporting observations established throughout the literature. Because of limited data and because other factors can affect acidity on a local scale, however, there is not enough evidence presented here to assess carbon dioxide‐driven acidification specifically in New York. The assertion that stormwater runoff has a larger influence than carbon dioxide on acidification in New York's coastal waters is presented with **medium** confidence, as it is based on observed patterns and documented correlations between acidity and nitrogen for specific water bodies, but not a formal attribution study.

### 9.5 Key Finding 5

**New York State's lakes and rivers have experienced increased water temperature, fluctuating water levels, and decreased ice cover, and these changes are expected to intensify in a warmer, wetter future**. Lakes are projected to experience more severe summer heat waves and decreased winter ice cover as temperatures rise in the coming decades. The Great Lakes could experience greater year‐to‐year variability in water levels, driven by periods of drought and extreme precipitation. Flood intensity and damages are expected to increase with extreme rainfall and broader changes in streamflow.

#### 9.5.1 Description of evidence base

Evidence of observed change in various attributes of New York's lakes and rivers comes from a variety of measurement programs:

- For **surface water temperature**, lines of evidence include satellite‐based measurement of surface water temperatures in the Great Lakes^151^ (Lakes Erie and Ontario warmed from 1995 to 2022—significant to a 95% confidence level following methods published by EPA^143^) and a standardized analysis of in situ data for the Northeast that included 15 smaller lakes in New York (14 of 15 experienced surface warming since 1975 or 1985, depending on when data collection started, and the overall trend across lakes in the region was significant to a 95% level).^146^ These results are consistent with continental and global‐scale studies that have documented widespread warming of lakes at temperate latitudes.^147^, ^153^ An increase in stream temperatures is inferred from measured data in neighboring states^149^ and statistical relationships with air temperature.^150^
- **Water levels** are based on long‐term gauging records for the Great Lakes, measured consistently since 1860. The data show fluctuations over time, with some record highs since 2015, but there is still much variation in the data set.^143^ Thus, the assertion in this key finding is more a general recognition that water levels have fluctuated over time, and should not be taken to indicate a definitive long‐term trend for both of New York's Great Lakes or for any other specific lakes in New York.
- Statistically significant changes in **ice cover** in lakes have been detected by several independent long‐term monitoring efforts, ranging from comprehensive daily and weekly ice mapping of the Great Lakes since 1973 (observations from ships, shore, aircraft, and satellites)^173^ to more than a century of shoreline observer records for Lake Champlain, Otsego Lake, Lake George, and additional smaller lakes in the Adirondacks.^170^, ^171^, ^172^ All significant changes in ice cover extent or duration have been decreases.

Evidence that many of these changes are expected to intensify comes from a variety of projections published in the literature. For example, evidence of future warming in New York's lakes comes from regionally downscaled climate modeling. Results include surface warming of all Great Lakes under two climate scenarios^157^; warming of Oneida Lake—the largest lake entirely within New York—under two climate scenarios^156^; and corroborating evidence from global studies and the prior work they cite.^154^ River and stream water temperatures could be expected to increase, too, but this assertion relies on association with air temperature (a predictive variable) and not direct modeling of water temperatures.

The projected increase in severe summer heat waves in lakes is based on a global modeling analysis.^154^ This analysis shows a large change, from an average of 8 heat wave days per year (over the period 1970–1999) to about 96 days by the end of this century. Applicability of this finding to New York is based on the assessment team's judgment, given (1) the strong projections of continued warming in New York's lakes (which would likely shift the entire temperature distribution, including the tails, just as the shifting distribution of air temperatures makes extreme events more common); (2) that lakes in the northeastern United States have warmed as quickly as or more quickly than those in other mid‐latitude regions of the world^146^; and (3) projections of increased duration of warm‐season stratification in New York lakes,^156^, ^157^ which exacerbates heat waves as it traps heat in the upper portion of the water column.^217^

Projections of decreased winter ice cover on New York's lakes come from regionally downscaled climate modeling for the Great Lakes and for smaller lakes such as Lower Saint Regis Lake in the Adirondacks.^157^, ^171^ These models show wide agreement that lakes will experience reduced ice cover as winter temperatures increase under multiple climate scenarios (SSP2‐4.5 and SSP5‐8.5 for the Great Lakes; SSP1‐2.6 and SSP5‐8.5 for Lower Saint Regis Lake). As noted in the traceable account for Key Finding 1, there is very high confidence that winter air temperatures will increase in New York.

Climate modeling is inconclusive as to whether Great Lakes water levels will increase or decrease over the decades ahead. Therefore, this key finding avoids suggesting a direction of change and instead reports what many models generally agree upon, which is that annual and multiyear variability in Great Lakes water levels will increase. Models suggest that “prolonged periods of both high and low levels” will become more common.^181^ The mechanism for such an increase in variability is conceivable given the general tendency of climate change to exacerbate extremes, including periods of relative drought and extreme precipitation events.^179^ Seasonal differences in projected precipitation and runoff could increase variability at a seasonal scale.^186^, ^189^

The assertion that flood intensity and damages are expected to increase with extreme rainfall reflects general agreement among a variety of modeling studies. These studies include projections for winter river flows and inland flooding in the northeastern United States,^165^ a combination of lake seiche and high river flows along Great Lakes shorelines,^166^ a doubling of projected financial damages from flooding across most regions of the United States associated with warming that this assessment projects to occur by mid‐century,^163^ and downscaled projections of inland flood damages that show increases for parts of New York.^164^ However, the latter study projects decreases for other parts of New York. Projected increases in fluvial flooding align with projections of increased annual stream discharge and higher winter streamflows in particular.^35^

#### 9.5.2 New information and remaining uncertainties

Observed trends in certain parameters for New York's lakes and rivers remain inconclusive or have limited data. For example, while individual lake temperatures across New York State are regularly monitored by government agencies, academic organizations, and individuals via programs such as the Citizens Statewide Lake Assessment Program, there is a lack of comprehensive analysis of these data. As noted above, time‐series analysis of temperature data for relatively unmodified streams is also limited—a possible opportunity for further study. Increases in inland flooding have been noted anecdotally, but statistical analysis is limited. In one analysis based on stream gauge data, only one of 10 New York sites had a statistically significant increase in flood magnitude—and none in frequency—from 1965 to 2015.^158^

Projections of stream temperatures have some uncertainty as they may be inferred from projections of air temperature. Increased streamflow associated with increased precipitation could offset some of this warming effect.^149^

Projections of future water levels in the Great Lakes are limited due to the difficulty of simulating land–air–lake processes and the high spatial resolution needed to generate accurate estimates.^179^, ^181^ Thus, there is no scientific consensus as to whether lake levels will increase or decrease overall.^181^ The more recent evidence discussed in Section 7.4 suggests a tendency toward higher water levels through the middle of this century, but that evidence still comes with appreciable uncertainties, along with the caveat that Lake Ontario's water levels are also influenced by downstream hydromodifications (namely, the Moses‐Saunders dam) that regulate outflow from the lake. More generally, it could take several more years to achieve standardized approaches to model Great Lakes processes, including ice cover.

#### 9.5.3 Assessment of confidence based on evidence

There is **very high** confidence that lakes in New York have warmed, will continue to warm in the decades ahead, and will experience more severe summer heat waves. Observed changes are supported by a preponderance of evidence showing statistically significant surface warming across large and small lakes throughout New York. Future projections reflect agreement across multiple studies and scenarios. These findings align with widely reported regional and global trends, and their fundamental connection to air temperature (which is also increasing with very high confidence, as discussed in Key Finding 1) is well established.

There is **medium** confidence that stream temperatures in New York have increased and will increase in the future. While an association with rising air temperatures has been established scientifically, analysis of direct observations and projections for stream temperatures in New York is limited, and the extent to which increased streamflow could offset the warming effect of air temperature is uncertain.

There is **very high** confidence that winter ice cover in New York lakes has declined and will continue to do so in the decades ahead. Although observed ice cover trends are indeterminate for certain portions of the Great Lakes when characterized in certain ways (e.g., maximum annual ice cover extent), most evidence points to declining ice cover duration across lakes in many parts of the state, including much of Lake Ontario. This direction of change is consistent across many independent monitoring efforts, some dating back more than a century. Projections of further loss reflect complete agreement across climate models, and their fundamental connection to air temperature (which is also increasing with very high confidence) is well established.

There is **medium** confidence that water levels in the Great Lakes will increase in variability. This conclusion comes from recent downscaled modeling, and it comes with a plausible explanation given the tendency of climate change to exacerbate extremes in wetness and drought. However, the modeling of Great Lakes water levels is admittedly more uncertain than the modeling for other phenomena discussed here, with models notably disagreeing on the overall direction of change. Thus, it would seem prudent to exercise some caution in interpreting any modeling related to Great Lakes water levels, and this portion of the finding is qualified with the word “could.”

There is **high** confidence that flood intensity and damages will increase during the 21st century. Such a conclusion is arguably intuitive when coupled with this assessment's projection of more frequent and intense heavy precipitation events (Key Finding 2; very high confidence). It is also supported by multiple modeling studies and other authoritative assessment literature. However, at least one study projects decreasing damages for sizable portions of New York,^164^ particularly with temperature changes anticipated for the first half of this century.

## AUTHOR CONTRIBUTIONS

C.L.: Drafting, revising, and editing the manuscript; review and general supervision. D.B.: Developing methodology; generating data. K.G.: Drafting the manuscript. R.H.: Developing methodology; generating data. K.J.: Drafting the manuscript. N.O.: Drafting the manuscript; creating and compiling figures. S.S.: Drafting the manuscript; creating and compiling figures.

## COMPETING INTERESTS

The authors declare no competing interests.

### PEER REVIEW

The peer review history for this article is available at https://publons.com/publon/10.1111/nyas.15240.
